# Correction: Active or Passive Exposure to Tobacco Smoking and Allergic Rhinitis, Allergic Dermatitis, and Food Allergy in Adults and Children: A Systematic Review and Meta-Analysis

**DOI:** 10.1371/journal.pmed.1001939

**Published:** 2016-02-02

**Authors:** The PLOS Medicine Staff

## Notice of Republication

This article was republished on October 19, 2015, to correct editor comments that were erroneously included in the main text. The publisher apologizes for the errors. Please download this article again to view the correct version. The originally published, uncorrected article and the republished, corrected article are provided here for reference.

In addition, the authors wish to make a correction: The original article duplicated Fig 2 under the caption for Fig 7. The authors have provided the correct version of Fig 7, which can be found here.

**Fig 7:**
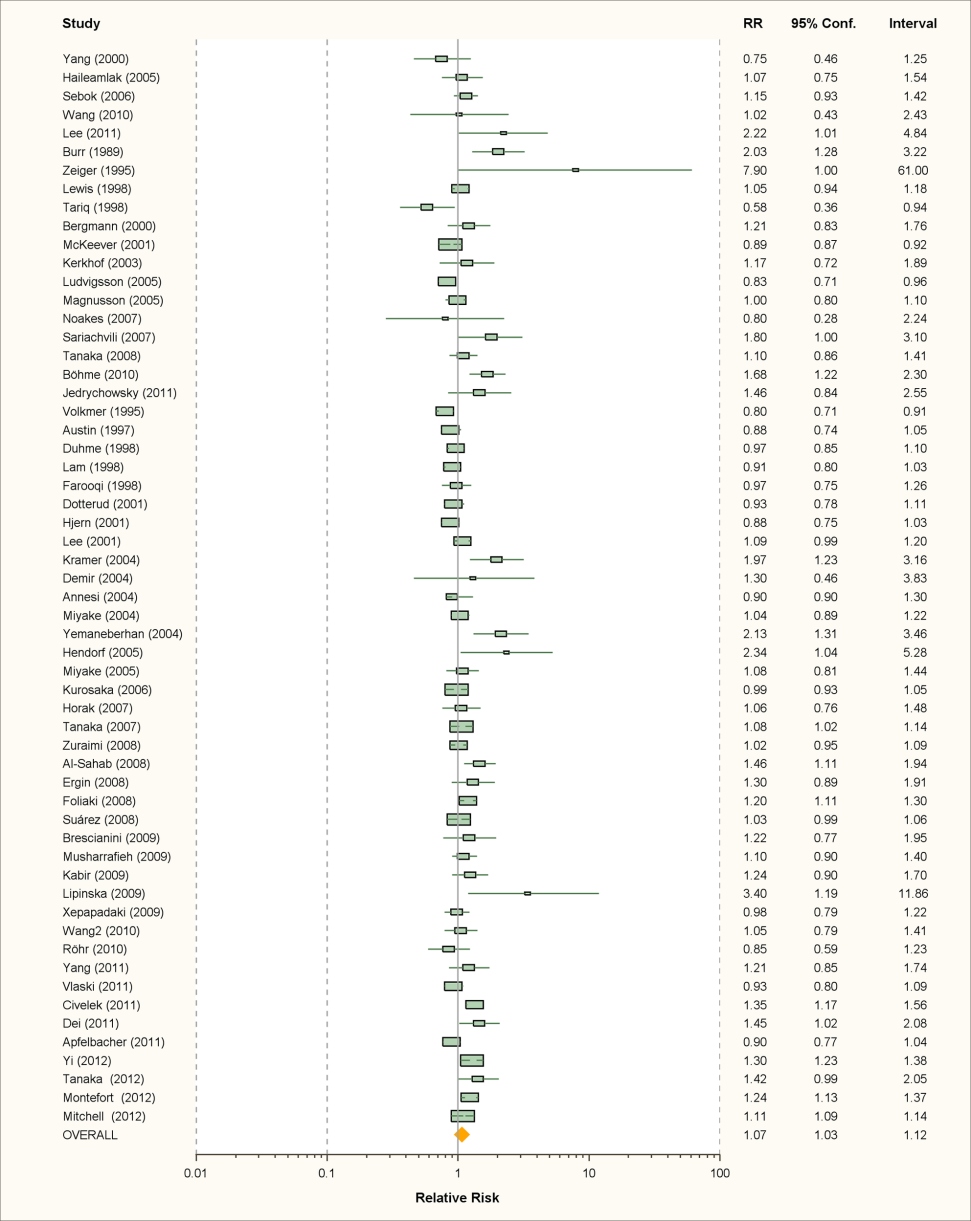
Study-specific and random effects pooled relative risks of passive smoking and allergic dermatitis.

## Supporting Information

S1 File Originally published, uncorrected article. (PDF)

S2 File Republished, corrected article. (PDF)
