# Supplementary material for: Active or Passive Exposure to Tobacco Smoking and Allergic Rhinitis, Allergic Dermatitis, and Food Allergy in Adults and Children: A Systematic Review and Meta-Analysis
Source: PLoS Med. 2014 Mar 11;11(3):e1001611. doi: 10.1371/journal.pmed.1001611 (PMC3949681; doi:10.1371/journal.pmed.1001611)
Supplement: Table S1 — Quality scoring of allergic rhinitis, dermatitis, and food allergies studies. (DOC) [file pmed.1001611.s001.doc]

**Quality scoring of allergic rhinitis studies**

**CRITERIA:**

**1.**

**FOR SMOKING DEFINITION:**

**Duration** – duration of smoking considered

“or”

**Nº** - number of cigarettes smoked (usually per day/per year)

**2.**

**Determination of allergic rhinitis well defined, with one of these:**

-SKP “or”

- IgE “or”

- Medical diagnosis

**3.**

Adjusted for: - Sex, Age and Any other.

**4.**

Participation rate ≥ 80%

**5.**

Clearly defined target population

**Abbreviations:**

**AS – active smoking**

**SHS – second hand smoking**

**SHS in Utero – mothers smoking during pregnancy**

**SHS + in Utero** – second hand smoking + mothers smoking during pregnancy

| Nº | **Author (Year)** | **OR calculated** | **1. Smoking well defined**  - Duration “or”  -Nº | **2. AR diagnosis well defined**  -SKP “or”  - IgE “or”  - Medical diagnosis | **3. Adjusted for:**  - Sex  - Age  - Any other | **4. Participation rate ≥80%** | **5. Clearly defined target population** | **TOTAL**  **(MAX 5)** |
| --- | --- | --- | --- | --- | --- | --- | --- | --- |
|  | **I. Case-control studies** | | | | | | | |
| 1 | Ozasa K 1995 | AS  SHS | -Duration=0  -Nº=1  **1** | -SPT =0  - IgE=1  - Being positive of eosinophils in nasal discharge or positive to nasal provocation test..=1  **1** | - sex=0  - age=1  -any other=0  **0** | Unknown  **0** | Simply a convenience sample as for instance patients of a consultation  **0** | **2** |
| 2 | Cakir E 2010 | AS  SHS | -Duration=0  -Nº=1  **1** | -SPT =0  - IgE=0 (ISAAC)  **0** | - sex=1  - age=1  -any other=1  **1** | Not specified  **0** | Working adolescents  **0** | **2** |
| **3** | Lin SY 2011 | SHS | -Duration=1  -Nº=0  **1** | -SPT=0  - IgE=0  **0** | - sex=1  - age=1  -any other=1  **1** | 35%  **0** | Cohort of nonsmokers in Washington country  **1** | **3** |
| **4** | Miyake Y 2011 | AS  Women only | -Duration=0  -Nº=0  **0** | -SPT=0  - IgE=0  **0** | - sex=1  - age=0  -any other=0  **0** | 84,9%  **1** | All pregnant women in the hospitals  **1** | **2** |
|  | **II. Cohort studies** | | | | | | | |
| Nº | **Author (Year)** | **OR calculated** | **1. Smoking well defined**  - Duration “or”  -Nº | **2. AR diagnosis well defined**  -SKP “or”  - IgE “or”  - Medical diagnosis | **3. Adjusted for:**  - Sex  - Age  - Any other | **4. Participation rate ≥80%** | **5. Clearly defined target population** | **TOTAL**  **(MAX 5)** |
| 1 | Wright AL (1994) | SHS | -Duration=0  -Nº=1  **1** | -SPT =1  - IgE=1  **1** | - sex=0  - age=0  -any other=0  **0** | 77%  **0** | From Tucson Children Resp. survey  **1** | **3** |
| 2 | Annesi-Maesano (1997) | AS  (men only) | -Duration=0  -Nº=1  **1** | -SPT =0  - IgE=0  **0** | - sex=0  - age=1  -any other=0  **0** | 65%  **0** | All police officers  **0** | **1** |
| 3 | Lewis SA (1998) | SHS  SHS in Utero | -Duration=0  -Nº=1  **1** | -SPT =0  - IgE=0  **0** | - sex=1  - age=1  -any other=1  **1** | 69% (5-10years)  54% (16years)  **0** | British Birth Cohort: all children  **1** | **3** |
| 4 | Shaheen SO (1999) | AS  SHS in Utero | -Duration=0  -Nº=1  **1** | -SPT =0  - IgE=0  **0** | - sex=1  - age=1  -any other=1  **1** | 66%  **0** | British Birth Cohort: all children  **1** | **3** |
| 5 | Bergmann 2000 | SHS | -Duration=0  -Nº=0  **0** | -SPT =0  - IgE=1  **1** | - sex=1  - age=1  -any other=1  **1** | 17.3%  (67.0%after follow-up)  **0** | Germany Birth Cohort  **1** | **3** |
| 6 | Tariq 2000 | SHS  SHS in utero | -Duration=0  -Nº=0  **0** | -SPT =0  - IgE=0  **0** | - sex=0  - age=1  -any other=0  **0** | 79.3%  0 | All newborns in certain period  1 | **1** |
| 7 | McKeever TM (2001) | SHS | -Duration=0  -Nº=1  **1** | -SPT =0  - IgE=0  **0** | - sex=1  - age=1  -any other=1  **1** | Not specified  0 | Historical Birth Cohort of West Midland  **1** | **3** |
| 8 | Magnusson LL (2005) | SHS  SHS in Utero  SHS + in Utero | -Duration=0  -Nº=1  **1** | -SPT =0  - IgE=0  **0** | - sex=1  - age=1  -any other=1  **1** | 74%  **0** | All pregnant women during period  **1** | **2** |
| 9 | Johansson AK  (2008) | SHS  SHS in Utero | -Duration=0  -Nº=0  **0** | -SPT =0  - IgE=0  **0** | - sex=0  - age=1  -any other=1  **0** | 51,9 %  **0** | - All babies in South west Sweden  **1** | **1** |
| 10 | Nagata C (2008) | AS | -Duration=1  -Nº=1  **1** | -SPT =0  - IgE=0  **0** | - sex=1  - age=1  -any other=1  **1** | 82%  **1** | From population based cohort study  **1** | **4** |
| Nº | **Author (Year)** | **OR calculated** | **1. Smoking well defined**  - Duration “or”  -Nº | **2. AR diagnosis well defined**  -SKP “or”  - IgE “or”  - Medical diagnosis | **3. Adjusted for:**  - Sex  - Age  - Any other | **4. Participation rate ≥80%** | **5. Clearly defined target population** | **TOTAL**  **(MAX 5)** |
| 11 | Bendtsen P (2008) | AS  (female only) | -Duration=0  -Nº=1  **1** | -SPT =0  - IgE=0  **0** | - sex=1  - age=1  -any other=1  **1** | 87%  **1** | randomly sampled general female population  **1** | **4** |
| 12 | Keil T (2009) | SHS + in Utero | -Duration=0  -Nº=0  - **Cotinine level** in blood serum  1 | -SPT =0  - IgE=0 (ISAAC)  **0** | - sex=1  - age=1  -any other=1  **1** | 73%  **0** | Germany Birth Cohort  **1** | **3** |
| 13 | Codispoti CD (2010) | SHS | -Duration=0  -Nº=1  **1** | -SPT =1  - IgE=0  -(Examination)  **1** | - sex=0  - age=1  -any other=1  **0** | Not specified  0 | CCAAPS cohort  **1** | **3** |
|  |  | | | | | | | |
|  | **III. Cross-Sectional Studies** | | | | | | | |
| Nº | **Author (Year)** | **OR calculated** | **1. Smoking well defined**  - Duration “or”  -Nº | **2. AR diagnosis well defined**  -SKP “or”  - IgE “or”  - Medical diagnosis | **3. Adjusted for:**  - Sex  - Age  - Any other | **4. Participation rate ≥80%** | **5. Clearly defined target population** | **TOTAL**  **(MAX 5)** |
| **1** | Bakke P (1990) | AS | -Duration=0  -Nº=0  0 | I phase:  -SPT=0  - IgE=0  0 | - sex=1  - age=1  -any other=1  1 | I-90%  II-86%  1 | Population comprised  1 | **3** |
| **2** | Leuenberger P (1994) | SHS | -Duration=0  -Nº=1  1 | -SPT=0  - IgE=1  1 | - sex=0  - age=0  -any other=0  0 | 80%  1 | (SPALDIA) A random selection of adults from 8 representative cities of Switzerland  1 | **4** |
| **3** | Ng TP (1994) | AS | -Duration=0  -Nº=1  1 | -SPT=1  - IgE=0  1 | - sex=0  - age=1  -any other=1  0 | 72.8%  0 | A stratified two-stage cluster disproportionate sampling  1 | **3** |
| **4** | Moyes CD (1995) | SHS | -Duration=0  -Nº=0  0 | -SPT=0  - IgE=0  0 | - sex=0  - age=0  -any other=0  0 | 85%  1 | All schools  1 | **2** |
| Nº | **Author (Year)** | **OR calculated** | **1. Smoking well defined**  - Duration “or”  -Nº | **2. AR diagnosis well defined**  -SKP “or”  - IgE “or”  - Medical diagnosis | **3. Adjusted for:**  - Sex  - Age  - Any other | **4. Participation rate ≥80%** | **5. Clearly defined target population** | **TOTAL**  **(MAX 5)** |
| **5** | Wutrich B (1996) | AS | -Duration=0  -Nº=0  0 | -SPT=1  - IgE=1  1 | - sex=1  - age=1  -any other=1  1 | Not specified  0 | (SPALDIA) Subjects from the 8 study areas  0 | **2** |
| **6** | Min Y –G (1997) | AS | -Duration=0  -Nº=0  0 | -SPT=1  - IgE=0  1 | - sex=0  - age=1  -any other=0  0 | 90,2%  1 | Multi-stage cluster-stratified randomly selected subjects from 60 districts throughout the country  1 | **3** |
| **7** | Siracusa A (1997) | AS | -Duration=0  -Nº=0  0 | -SPT=0  - IgE=0  0 | - sex=1  - age=1  -any other=1  1 | 61%  0 | A randomized sample from the National Health Service list of all Perugia area  1 | **2** |
| **8** | Austin JB (1997) | SHS  SHS in Utero | -Duration=0  -Nº=0  0 | -SPT=0  - IgE=0  0 | - sex=0  - age=1  -any other=0  0 | 85%  1 | Children attending secondary schools (Ref 3)  1 | **2** |
| **9** | Farooqi IS (1998) | SHS | -Duration=0  -Nº=0  0 | -SPT=0?  - IgE=0?  0 | - sex=0  - age=0  -any other=0  0 | 36,7%  0 | Oxford shire general practice cohort  0 | **0** |
| **10** | Lam TH (1998) | AS  SHS | -Duration=0  -Nº=1  1 | -SPT=0  - IgE=0  0 | - sex=1  - age=1  -any other=1  1 | 96%  1 | Randomly selected classes from randomly selected schools  1 | **4** |
| **11** | Ponsonby A-L (1998) | SHS | -Duration=0  -Nº=0  0 | -SPT=0  - IgE=0  0 | - sex=0  - age=0  -any other=0  0 | 89%  1 | All schools, home learning & distance education organizations  0 | **1** |
| **12** | Montefort S (1998) | AS  SHS | -Duration=0  -Nº=0  0 | -SPT=0  - IgE=0  0 | - sex=1  - age=1  -any other=1  1 | 88,7%  1 | randomly selected schools of Malta and Gozo  0 | **2** |
| **13** | Duhme H (1998) | AS  SHS | -Duration=1  -Nº=0  1 | -SPT=0  - IgE=0  0 | - sex=1  - age=1  -any other=0  0 | 81,2% & 85,9%  1 | All schools in each region, with all children target  1 | **3** |
| **14** | Burr ML (1999) | AS  SHS | -Duration=0  -Nº=0  0 | -SPT=0  - IgE=0  0 | - sex=1  - age=1  -any other=1  1 | 79,3%  0 | ISAAC.  Randomly selected schools of each region  1 | **2** |
| Nº | **Author (Year)** | **OR calculated** | **1. Smoking well defined**  - Duration “or”  -Nº | **2. AR diagnosis well defined**  -SKP “or”  - IgE “or”  - Medical diagnosis | **3. Adjusted for:**  - Sex  - Age  - Any other | **4. Participation rate ≥80%** | **5. Clearly defined target population** | **TOTAL**  **(MAX 5)** |
| **15** | Dotterud 1999 | AS | -Duration=0  -Nº=0  0 | -SPT=0  - IgE=0  0 | - sex=1  - age=1  -any other=1  1 | Nickel(Russia)-93,6%  1 | 0 | **1** |
| **16** | Keles N (1999) | AS  SHS | -Duration=0  -Nº=0  0 | -SPT=0  - IgE=0  0 | - sex=1  - age=1  -any other=1  1 | Not specified  0 | 2 schools from different polluted areas  1 | **2** |
| **17** | Plaschke PP (2000) | AS | -Duration=0  -Nº=1  1 | -SPT=0  - IgE=0  0 | - sex=1  - age=1  -any other=1  1 | 86%  1 | ECRH: random sample of the general population from 3 areas of Sweden  1 | **4** |
| **18** | Zacharasiewicz A (2000) | SHS  SHS in Utero | -Duration=0  -Nº=1  1 | -SPT=0  - IgE=0  0 | - sex=1  - age=1  -any other=1  1 | 63,7%  0 | 1 | **3** |
| **19** | Upton MN (2000) | AS | -Duration=0  -Nº=0  0 | -SPT=1  - IgE=0  1 | - sex=0  - age=1  -any other=0  0 | 78%  0 | All residents of 2 cities of west Scotland  1 | **2** |
| **20** | Ozdemir N (2000) | AS | -Duration=0  -Nº=0  0 | -SPT=0  - IgE=0  0 | - sex=0  - age=1  -any other=0  0 | 94,5%  1 | Newly enrolled university freshman  0 | **1** |
| **21 a** | Hjern A (2001a) | SHS | -Duration=0  -Nº=1  1 | -SPT=0  - IgE=0  0 | - sex=1  - age=1  -any other=1  1 | 80%  1 | Simple random sample  1 | **4** |
| **21 b** | Hjern A (2001b) | AS | -Duration=0  -Nº=1  1 | -SPT=0  - IgE=0  0 | - sex=1  - age=1  -any other=1  1 | 80%  1 | Simple random sample  1 | **4** |
| **22** | Janson C (2001) | SHS | -Duration=1  -Nº=0  1 | -SPT=0  - IgE=0  0 | - sex=1  - age=1  -any other=1  1 | Not specified  0 | Randomly selected persons of the same sex and age in the different centers (36 centers in 16 countries)  1 | **3** |
| **23** | Simpson BM (2001) | AS | -Duration=0  -Nº=0  0 | -SPT=0  - IgE=1  1 | - sex=1  - age=0  -any other=1  0 | Not specified  0 | All pregnant women attending two hospitals  1 | **2** |
| Nº | **Author (Year)** | **OR calculated** | **1. Smoking well defined**  - Duration “or”  -Nº | **2. AR diagnosis well defined**  -SKP “or”  - IgE “or”  - Medical diagnosis | **3. Adjusted for:**  - Sex  - Age  - Any other | **4. Participation rate ≥80%** | **5. Clearly defined target population** | **TOTAL**  **(MAX 5)** |
| **24** | Dotterud LK (2001) | AS | -Duration=0  -Nº=0  0 | -SPT=0  - IgE=0  0 | - sex=1  - age=1  -any other=1  1 | Nickel(Russia)-93,6%  1 | 0 | **2** |
| **25** | Kalyoncu (2001) | AS,  SHS | -Duration=0  -Nº=0  0 | -SPT=0  - IgE=0  0 | - sex=1  - age=1  -any other=1  1 | 85.8%  1 | 1 | **3** |
| **26** | Lee SII (2001) | SHS | -Duration=0  -Nº=0  0 | -SPT=0  - IgE=0  0 | - sex=1  - age=1  -any other=1  1 | 90.8%  1 | Random sample of elementary and middle school  1 | **3** |
| **27** | Stazi M-A (2002) | SHS | -Duration=0  -Nº=0  -(Carbon Monohydrate assessment=1)  1 | -SPT=0  - IgE=0  0 | - sex=1  - age=1  -any other=0  0 | 95%  1 | Sample from the vaccination center of a rural zone  0 | **2** |
| **28** | Peroni DG (2003) | SHS | -Duration=0  -Nº=0  0 | -SPT=1  - IgE=0  1 | - sex=1  - age=1  -any other=1  1 | 92%  1 | Randomly selected 18 nursery schools in Verone (Italy)  1 | **4** |
| **29** | Barraza Villarreal A (2003) | SHS  SHS in Utero | -Duration=0  -Nº=0  0 | -SPT=0  - IgE=0  0 | - sex=0  - age=0  -any other=0(not always)  0 | 92%  1 | The city divided into 3 areas according to pollution – schools  1 | **2** |
| **30** | Monteil MA (2004) | SHS | -Duration=0  -Nº=0  0 | -SPT=0  - IgE=0  0 | - sex=0  - age=0  -any other=0  0 | 50,2% & 47,5%  0 | Randomly selected 20% cities  1 | **1** |
| **31** | Lee S-L (2004) | SHS | -Duration=0  -Nº=0  0 | -SPT=0  - IgE=0  0 | - sex=1  - age=1  -any other=1  1 | 95%  1 | Random selected schools in Hong Kong  1 | **3** |
| **32** | Kramer U(2004) | SHS | -Duration=0  -Nº=0  -Cotinine spot in the urine sample=1  1 | -SPT=1  - IgE=0  1 | - sex=1  - age=1  -any other=1  1 | 68%  0 | Children registered for admission to school in one year  1 | **4** |
| **33** | Demir AU (2004) | SHS | -Duration=0  -Nº=0  0 | -SPT=0  - IgE=0  0 | - sex=0  - age=0  -any other=0  0 | 85%  1 | 1 school  0 | **1** |
| Nº | **Author (Year)** | **OR calculated** | **1. Smoking well defined**  - Duration “or”  -Nº | **2. AR diagnosis well defined**  -SKP “or”  - IgE “or”  - Medical diagnosis | **3. Adjusted for:**  - Sex  - Age  - Any other | **4. Participation rate ≥80%** | **5. Clearly defined target population** | **TOTAL**  **(MAX 5)** |
| **34** | Miyake Y (2004) | SHS | -Duration=0  -Nº=0  0 | -SPT=0  - IgE=0  0 | - sex=1  - age=1  -any other=1  1 | 61%  0 | All schools in the region  1 | **2** |
| **35** | A-Maesano I (2004) | AS  SHS | -Duration=0  -Nº=0  0 | -SPT=0  - IgE=0  0 | - sex=1  - age=0  -any other=1  0 | 82%  1 | School children from five different centers  1 | **2** |
| **36** | De S (2005) | SHS | -Duration=0  -Nº=0  0 | -SPT=0  - IgE=0  - Med.diagn.=1  0 | - sex=0  - age=0  -any other=0  0 | Not specified  0 | Children from outpatient department  0 | **0** |
| **37** | Topp R (2005) | SHS | -Duration=0  -Nº=1  1 | -SPT=0  - IgE=0  0 | - sex=1  - age=1  -any other=1  1 | 84,7%  1 | The data from German National Health Interview and Examination Survey  1 | **4** |
| **38** | Maziak W (2005) | SHS | -Duration=1  -Nº=1  1 | -SPT=0  - IgE=0  0 | - sex=1  - age=1  -any other=1  1 | 86%  1 | Stratified cluster sampling, with randomly selection  1 | **4** |
| **39** | Miyake Y (2005) | AS  SHS  (women) | -Duration=1  -Nº=1  1 | -SPT=0  - IgE=0  0 | - sex=1  - age= 1  -any other=1  1 | 17,2%  0 | Women, who become pregnant in one city during 3 years  1 | **3** |
| **40** | Bugiani M (2005) | AS | -Duration=0  -Nº=0  0 | -SPT=0  - IgE=0  0 | - sex=0  - age=0  -any other=0  0 | 75%  0 | Random sample of adult general population of 5 cities in Italy  1 | **1** |
| **41** | Obihara CC (2005) | SHS in Utero | -Duration=0  -Nº=0  0 | -SPT=0  - IgE=0  0 | - sex=1  - age=1  -any other=1  1 | 88%  1 | 15% of randomly selected houses in the area  1 | **3** |
| **42** | Strumylaite 2005 | SHS | -Duration=0  -Nº=0  0 | -SPT=0  - IgE=0  0 | - sex=0  - age=1  -any other=0  0 | 58.6%-69.2%  0 | 20 kindergarten of Kaunas city  0 | **0** |
| **43** | Lund VJ (2006) | AS  SHS | -Duration=0  -Nº=0  0 | -SPT=0  - IgE=0  0 | - sex=1  - age=1  -any other=0  0 | Not specified  0 | 1 | **1** |
| Nº | **Author (Year)** | **OR calculated** | **1. Smoking well defined**  - Duration “or”  -Nº | **2. AR diagnosis well defined**  -SKP “or”  - IgE “or”  - Medical diagnosis | **3. Adjusted for:**  - Sex  - Age  - Any other | **4. Participation rate ≥80%** | **5. Clearly defined target population** | **TOTAL**  **(MAX 5)** |
| **44** | Kurosaka F (2006) | SHS | -Duration=0  -Nº=0  0 | -SPT=0  - IgE=0  0 | - sex=1  - age=0  -any other=1  0 | 99% & 99,3%  1 | 58 primary schools of Himeji (Japan)  1 | **2** |
| **45** | Sakar 2006 | AS | -Duration=0  -Nº=0  0 | -SPT=0  - IgE=0  0 | - sex=0  - age=0  -any other=0  0 | 88.8%  1 | Household records of the 9 health centers of Manisa city  1 | 2 |
| **46** | Sai Yin Ho (2007) | SHS | -Duration=1  -Nº=0  1 | -SPT=0  - IgE=0  0 | - sex=1  - age=1  -any other=1  1 | 64,7% & 61,2% (stage II-64,9%)  0 | Randomly selected telephone Nº  1 | **3** |
| **47** | Horak E (2007) | SHS  SHS in Utero | -Duration=0  -Nº=0  0 | -SPT=0  - IgE=0  0 | - sex=1  - age=1  -any other=1  1 | 42%  0 | Randomly selected 100 kinder gardens (out of 435 in Tyrol(Austria)  1 | **2** |
| **48** | Ebbert JO (2007) | SHS | -Duration=1  -Nº=0  1 | -SPT=0  - IgE=0  0 | - sex=0  - age=0  -any other=0  0 | 6,7%  0 | Subjects from member database of Flight Attendants  1 | **2** |
| **49** | Tanaka K (2007) | SHS | -Duration=0  -Nº=1  1 | -SPT=1  - IgE=1  1 | - sex=1  - age=1  -any other=1  1 | 60,3%  0 | All schools  1 | **4** |
| **50** | Zuraimi MS (2007) | SHS | -Duration=0  -Nº=1  1 | -SPT=0  - IgE=0  0 | - sex=1  - age=1  -any other=1  1 | 70%  0 | Randomly selected child care centers(18%) in Singapore  1 | **3** |
| **51** | Foliaki S (2008) | SHS | -Duration=0  -Nº=0  0 | -SPT=0  - IgE=0  0 | - sex=1  - age=1  -any other=1  1 | (Results of 8 distinct countries)  0 | 8 Pacific countries  0 | **1** |
| **52** | Gomez R (2008) | AS | -Duration=0  -Nº=0  0 | -SPT=0  - IgE=0  0 | - sex=0  - age=0  -any other=0  0 | Not specified  0 | 1 | **1** |
| **53** | Kabir Z (2009) | SHS | -Duration=0  -Nº=0  0 | -SPT=0  - IgE=0  0 | - sex=1  - age=0  -any other=1  0 | 90%  1 | Stratified random sampling of schools  1 | **2** |
| Nº | **Author (Year)** | **OR calculated** | **1. Smoking well defined**  - Duration “or”  -Nº | **2. AR diagnosis well defined**  -SKP “or”  - IgE “or”  - Medical diagnosis | **3. Adjusted for:**  - Sex  - Age  - Any other | **4. Participation rate ≥80%** | **5. Clearly defined target population** | **TOTAL**  **(MAX 5)** |
| **54** | Brescianini S (2009) | SHS | -Duration=0  -Nº=0  0 | -SPT=0  - IgE=0  0 | - sex=1  - age=1  -any other=1  1 | Not specified  0 | Children from 3 schools  0 | **1** |
| **55** | Musharrafieh U (2009) | SHS | -Duration=0  -Nº=0  0 | -SPT=0  - IgE=0  0 | - sex=1  - age=1  -any other=1  1 | Not specified  0 | Random sample of 55 schools and only 13 agreed to participate  0 | **1** |
| **56** | Gonzalez-Diaz SN (2010) | SHS | -Duration=0  -Nº=0  0 | -SPT=0  - IgE=0  0 | - sex=0  - age=0  -any other=0  0 | 92%  1 | All schools in three cities of Mexico  1 | **2** |
| **57** | Bedolla-Barajas M (2010) | SHS | -Duration=0  -Nº=0  0 | -SPT=0  - IgE=0  0 | - sex=0  - age=0  -any other=0  0 | 6,5%  0 | Muestreo probabilístico estratificado y por conglomerados  1 | **1** |
| **58** | Wang H-Y (2010) | SHS | -Duration=0  -Nº=0  0 | -SPT=0  - IgE=0  0 | - sex=1  - age=1  -any other=1  1 | 97,6%  1 | Randomly selected schools from each region  1 | **3** |
| **59** | Vlaski E (2010) | SHS | -Duration=0  -Nº=0  0 | -SPT=0  - IgE=0  0 | - sex=1  - age=1  -any other=1  1 | 90.9%  1 | 17 randomly selected schools in the region  1 | **3** |
| **60** | Virkkula P (2011) | SHS | -Duration=0  -Nº=0  0 | -SPT=0  - IgE=1  1 | - sex=0  - age=1  -any other=0  0 | 71,8%  0 | A random sample from the children register, which were divided into snorers and non-snorers as a controls  1 | **2** |
| **61** | Hakansson K (2011) | AS | -Duration=0  -Nº=1  1 | -SPT=0  - IgE=1  1 | - sex=1  - age=1  -any other=0  0 | 80%  1 | Random sample of subjects invited to health examination  1 | **4** |
| **62** | Chen 2012 | SHS in Utero | -Duration=0  -Nº=0  0 | -SPT=0  - IgE=0  0 | - sex=0  - age=0  -any other=0  0 | 74.5%  (48.5%)  0 | 3 elementary and 2 middle schools  1 | 1 |
| **63** | Peñaranda 2012 | SHS (6-7years)  AS (13-14years) | -Duration=0  -Nº=0  0 | -SPT=0  - IgE=0  0 | - sex=0  - age=1  -any other=1  0 | 89.5% & 98.7%  1 | Randomly selected 74 (for 6-7years) and 48 (for 13-14 years) schools  1 | 2 |
| Nº | **Author (Year)** | **OR calculated** | **1. Smoking well defined**  - Duration “or”  -Nº | **2. AR diagnosis well defined**  -SKP “or”  - IgE “or”  - Medical diagnosis | **3. Adjusted for:**  - Sex  - Age  - Any other | **4. Participation rate ≥80%** | **5. Clearly defined target population** | **TOTAL**  **(MAX 5)** |
| **64** | Tanaka K, 2012  ISAAC q | AS  Women only | -Duration=1  -Nº=1  1 | -SPT=0  - IgE=0  0 | - sex=1  - age=1  -any other=1  1 | 99.2%  1 | 423  obstetric hospitals  1 | **4** |
| **65** | Montefort (2012)  ISAAC III | AS  SHS | -Duration=1  -Nº=1  1 | -SPT=0  - IgE=0  0 | - sex=0  - age=1  -any other=0  0 | 80% (5-8year)  90% (13-15year)  1 | All state primary schools in Malta and Gozo were randomly  Chosen(44+18 schools)  1 | **3** |
| **66** | Mitchell 2012  ISAACIII | SHS | -Duration=0  -Nº=1  1 | -SPT=0  - IgE=0  0 | - sex=1  - age=1  -any other=1  1 | Not specified  0 | Random sample of schools  1 | **3** |

**Quality scoring of dermatitis studies**

**CRITERIA:**

**1.**

**FOR SMOKING DEFINITION:**

**Duration** – duration of smoking considered

“or”

**Nº** - number of cigarettes smoked (usually per day/per year)

**2.**

**Determination of ATOPIC DERMATITIS:**

**A= 1** – A physician had diagnosed dermatitis or cases were from dermatology consults, with actual signs of the dermatitis.

B= 0 – Asking only by Questionnaire: physician had ever diagnosed this condition?

C= 0 – Self reported AD in a questionnaire.

**3.**

Adjusted for: - Sex, Age and Any other.

**4.**

Participation rate ≥ 80%

**5.**

Clearly defined target population

**Abbreviations:**

AS – active smoking

SHS – second hand smoking

SHS in Utero – mothers smoking during pregnancy

**SHS + in Utero – second hand smoking + mothers smoking during pregnancy**

| Nº | | **Author (Year)** | **OR calculated** | | | **1. Smoking well defined**  - Duration “or”  -Nº | | | **2. DERM. diagnosis well defined**  -A diagnosis (1)  - B,C auto diagnosis)0) | | | **3. Adjusted for:**  - Sex  - Age  - Any other | | | **4. Participation rate ≥80%** | | | **5. Clearly defined target population** | | | | **TOTAL**  **(MAX 5)** | | |
| --- | --- | --- | --- | --- | --- | --- | --- | --- | --- | --- | --- | --- | --- | --- | --- | --- | --- | --- | --- | --- | --- | --- | --- | --- |
|  | **I. Case-control studies** | | | |  | | |  | | |  | | |  | | |  | | |  |  | | |  |
| 1 | | Mills 1994 | AS | | | -Duration=1  -Nº=1  1 | | | -A  1 | | | - sex=0  - age=0  -any other=0  0 | | | 85%  1 | | | 1 | | | | **4** | | |
| 2 | | Yang 2000 | SHS | | | -Duration=0  -Nº=0  0 | | | - B  0 | | | - sex=1  - age=1  -any other=1  1 | | | 95%  1 | | | 1 | | | | **3** | | |
| 4 | | Purvis 2005 | SHS in utero | | | -Duration=0  -Nº=0  0 | | | - A  1 | | | - sex=0  - age=1  -any other=0  0 | | | 63,2%  0 | | | 1 | | | | **2** | | |
| 5 | | Haileamlak 2005  ISAACq | SHS | | | -Duration=0  -Nº=0  0 | | | - C (I & II)  0 | | | - sex=0  - age=1  -any other=0  0 | | | 65.9%  0 | | | 1 | | | | **1** | | |
| 3 | | Sebok 2006 | SHS | | | -Duration=0  -Nº=0  0 | | | - A  1 | | | - sex=1  - age=1  -any other=1  1 | | | 95%  1 | | | 0 | | | | **3** | | |
| 6 | | Wang 2010 ISAACq | SHS | | | -Duration=0  -Nº=0  0 | | | B & C – self reported (ISAAC)  0 | | | - sex=0  - age=0  -any other=0  0 | | | 28.9%  0 | | | 1 | | | | **1** | | |
| 7 | | Cakir 2010  ISAACq | AS | | | -Duration=0  -Nº=1  1 | | | -C  0 | | | - sex=1  - age=1  -any other=1  1 | | | Not specified  0 | | | Working adolescents  0 | | | | **2** | | |
| 8 | | Lee CH 2011 | AS  SHS | | | -Duration=1  -Nº=1  1 | | | -A  1 | | | - sex=1  - age=1  -any other=0  0 | | | 85%(cases)  88%(controls)  1 | | | Cases from 1 medical center  0 | | | | **3** | | |
| 9 | | Miyake 2012 (AD) | AS | | | -Duration=0  -Nº=0  0 | | | -C  0 | | | - sex=1  - age=0  -any other=0  0 | | | 72.3%  0 | | | 1 | | | | **1** | | |
|  | | **II. Cohort studies** | | |  | |  | | |  | | |  | | |  | | |  | | | |  | |
| Nº | | **Author (Year)** | **OR calculated** | | | **1. Smoking well defined**  - Duration “or”  -Nº | | | **2. DERM. diagnosis well defined**  -A diagnosis (1)  - B,C auto diagnosis)0) | | | **3. Adjusted for:**  - Sex  - Age  - Any other | | | **4. Participation rate ≥80%** | | | **5. Clearly defined target population** | | | | **TOTAL**  **(MAX 5)** | | |
| 1 | | Burr 1989 | SHS | | | -Duration=0  -Nº=0  0 | | | A – examined by a pediatrician  1 | | | - sex=0  - age=0  -any other=0  0 | | | 88%  1 | | | 1 | | | | **3** | | |
| 2 | | Zeiger 1995 | SHS | | | -Duration=0  -Nº=0  0 | | | - A  1 | | | - sex=0  - age=1  -any other=0  0 | | | 57%  0 | | | 0 | | | | **1** | | |
| 3 | | Olesen 1997 | SHS in Utero | | | -Duration=0  -Nº=0  0 | | | - B (II study)  0 | | | - sex=1  - age=1  -any other=1  1 | | | 92%(IIstudy)  1 | | | All infants born during certain period in certain zone  1 | | | | **3** | | |
| 4 | | Lewis 1998 | SHS  SHS in Utero | | | -Duration=0  -Nº=1  1 | | | - C  0 | | | - sex=1  - age=1  -any other=1  1 | | | 69% (5-10years)  54% (16years)  0 | | | British Birth Cohort: all children  1 | | | | **3** | | |
| 5 | | Tariq 1998 | SHS | | | -Duration=0  -Nº=0  0 | | | - C  0 | | | - sex=0  - age=1  -any other=0  0 | | | 83,6%  1 | | | All infants during certain period  1 | | | | **2** | | |
| 6 | | Shaheen 1999 | AS  SHS in Utero | | | -Duration=0  -Nº=1  1 | | | -C  0 | | | - sex=1  - age=1  -any other=1  1 | | | 66,4%  0 | | | British Birth Cohort (26-year old respondents)  1 | | | | **3** | | |
| 7 | | Bergmann 2000 | SHS | | | -Duration=0  -Nº=0  0 | | | - A (child)  1 | | | - sex=1  - age=1  -any other=1  1 | | | 17.3%  (67.0%after follow-up)  0 | | | Germany Birth Cohort  1 | | | | **3** | | |
| 8 | | McKeever 2001 | SHS | | | -Duration=0  -Nº=0  0 | | | - A  1 | | | - sex=1  - age=1  -any other=1  1 | | | Not specified  0 | | | Historical Birth Cohort of West Midland  1 | | | | **3** | | |
| 9 | | Bergmann 2002 | SHS in Utero | | | -Duration=0  -Nº=0  0 | | | - A  1 | | | - sex=1  - age=1  -any other=1  1 | | | 17.3%  (71.5%after follow-up)  0 | | | 1 | | | | **3** | | |
| Nº | | **Author (Year)** | **OR calculated** | | | **1. Smoking well defined**  - Duration “or”  -Nº | | | **2. DERM. diagnosis well defined**  -A diagnosis (1)  - B,C auto diagnosis)0) | | | **3. Adjusted for:**  - Sex  - Age  - Any other | | | **4. Participation rate ≥80%** | | | **5. Clearly defined target population** | | | | **TOTAL**  **(MAX 5)** | | |
| 10 | | Kerkhof 2003 | SHS | | | -Duration=1  -Nº=0  1 | | | - A  1 | | | - sex=1  - age=1  -any other=1  1 | | | 35%  0 | | | 1 | | | | **4** | | |
| 11 | | Ludvigsson 2005 | SHS | | | -Duration=0  -Nº=0  0 | | | - C  0 | | | - sex=0  - age=1  -any other=1  0 | | | 40,5%(78,6%)  0 | | | 1 | | | | **1** | | |
| 12 | | Magnusson 2005 | SHS  SHS in Utero  SHS + in Utero | | | -Duration=0  -Nº=1  1 | | | - B  - C  0 | | | - sex=1  - age=1  -any other=1  1 | | | 56,7%  0 | | | All pregnant women in the two Danish cities in certain period  1 | | | | **3** | | |
| 13 | | Linneberg 2006 | SHS in utero | | | -Duration=1  -Nº=0  1 | | | - B or/+  - C  0 | | | - sex=1  - age=1  -any other=1  1 | | | 67.0%  0 | | | 1 | | | | **3** | | |
| 14 | | Lerbaek 2007 | AS | | | -Duration=1  -Nº=1  1 | | | - C  0 | | | - sex=0  - age=0  -any other=0  0 | | | 81,7%  1 | | | 1 | | | | **3** | | |
| 15 | | Noakes 2007 | SHS + SHS in utero | | | -Duration=0  -Nº=0  0 | | | - A  1 | | | - sex=0  - age=1  -any other=0  0 | | | 67,2 %  0 | | | 1 | | | | **2** | | |
| 16 | | Sariachvili 2007 | SHS &(+) SHS in utero | | | -Duration=0  -Nº=0  0 | | | - C  0 | | | - sex=1  - age=1  -any other=1  1 | | | 47,5%  0 | | | 1 | | | | **2** | | |
| 17 | | Tanaka 2008 ISAACq | SHS &  SHS in utero | | | -Duration=0  -Nº=0  0 | | | - C  0 | | | - sex=1  - age=1  -any other=1  1 | | | 76,1%  0 | | | 1 | | | | **2** | | |
| 18 | | Böhme 2010 | SHS & SHS in utero | | | -Duration=1  -Nº=1  1 | | | - B or/&  - C  0 | | | - sex=1  - age=1  -any other=1  1 | | | 61,3 %  0 | | | 1 | | | | **3** | | |
| 19 | | Jedrychowski 2011 | SHS (+SHS in utero) | | | -Duration=0  -Nº=1  1 | | | - A  1 | | | - sex=0  - age=1  -any other=0  0 | | | 92,9%  1 | | | 1 | | | | **4** | | |
|  | | **III. Cross-Sectional Studies** | | | | | | | | | | | | | | | | | | | | | | |
| Nº | | **Author (Year)** | **OR calculated** | | | **1. Smoking well defined**  - Duration “or”  -Nº | | | **2. DERM. diagnosis well defined**  -A diagnosis (1)  - B,C auto diagnosis)0) | | | **3. Adjusted for:**  - Sex  - Age  - Any other | | | **4. Participation rate ≥80%** | | | **5. Clearly defined target population** | | | | **TOTAL**  **(MAX 5)** | | |
| **1** | | Edman 1988 | | AS | | -Duration=0  -Nº=0  0 | | | - A  1 | | | - sex=0  - age=0  -any other=0  0 | | | Not specified  0 | | | 0 | | | | **1** | | |
| **2** | | Bakke 1990 | | AS | | -Duration=0  -Nº=1  1 | | | - C(I)4992 &  - A(II)512  0 | | | - sex=1  - age=1  -any other=1  1 | | | 76.9%  0 | | | 1 | | | | **3** | | |
| **3** | | Volkmer 1995 | | SHS | | -Duration=0  -Nº=0  0 | | | - C  0 | | | - sex=0  - age=0  -any other=1  0 | | | 73%  0 | | | 1 | | | | **1** | | |
| **4** | | Austin 1997 | | SHS | | -Duration=0  -Nº=0  0 | | | - C  0 | | | - sex=0  - age=0  -any other=0  0 | | | 85.3%  1 | | | 1 | | | | **2** | | |
| **5** | | Liss 1997 | | AS | | -Duration=0  -Nº=0  0 | | | - A (SPT latex allergy)&  - C (latex allergy)  1 | | | - sex=0  - age=0  -any other=0  0 | | | 64.3%  0 | | | 1 | | | | **2** | | |
| **6** | | Schafer 1997 | | SHS in Utero & during lactation | | -Duration=0  -Nº=0  0 | | | - B  0 | | | - sex=0  - age=1  -any other=0  0 | | | 71.4%  0 | | | 1 | | | | **1** | | |
| **7** | | Duhme 1998  ISAAC | | AS  SHS | | -Duration=1  -Nº=0  1 | | | - C  0 | | | - sex=1  - age=1  -any other=0  0 | | | 83.5%  1 | | | All schools in each region, with all children target  1 | | | | **3** | | |
| **8** | | Lam 1998 | | AS  SHS | | -Duration=1  -Nº=1  1 | | | -C  0 | | | - sex=1  - age=1  -any other=1  1 | | | 96%  1 | | | Randomly selected classes from randomly selected schools  1 | | | | **4** | | |
| **9** | | Montefort 1998  ISAAC | | AS | | -Duration=0  -Nº=0  0 | | | -C &-B  0 | | | - sex=1  - age=0  -any other=1  0 | | | 88,7%  1 | | | 25 randomly selected schools of Malta and Gozo  1 | | | | **2** | | |
| Nº | | **Author (Year)** | **OR calculated** | | | **1. Smoking well defined**  - Duration “or”  -Nº | | | **2. DERM. diagnosis well defined**  -A diagnosis (1)  - B,C auto diagnosis)0) | | | **3. Adjusted for:**  - Sex  - Age  - Any other | | | **4. Participation rate ≥80%** | | | **5. Clearly defined target population** | | | | **TOTAL**  **(MAX 5)** | | |
| **10** | | Farooqi 1998 | | SHS | | -Duration=0  -Nº=0  0 | | | -B  -C  0 | | | - sex=0  - age=0  -any other=0  0 | | | 36.7%  0 | | | Oxford shire general practice cohort  0 | | | | **0** | | |
| **11** | | Dotterud 1999 | | AS | | -Duration=0  -Nº=0  0 | | | - C  0 | | | - sex=0  - age=0  -any other=0  0 | | | 93.6%  1 | | | 0 | | | | **1** | | |
| **12** | | Dotterud 2001 | | SHS | | -Duration=0  -Nº=0  0 | | | - C  0 | | | - sex=1  - age=1  -any other=1  1 | | | 93.6%  1 | | | 1 | | | | ***3*** | | |
| **13** | | Hjern 2001 | | SHS | | -Duration=0  -Nº=1  1 | | | - C  0 | | | - sex=1  - age=1  -any other=1  1 | | | 80%  1 | | | Simple random sample  1 | | | | **4** | | |
| **14** | | Lee SII 2001  ISAACq. | | SHS | | -Duration=0  -Nº=0  0 | | | - C  0 | | | - sex=1  - age=1  -any other=1  1 | | | 90.8%  1 | | | Random sample of elementary and middle school  1 | | | | **3** | | |
| **15** | | Simpson 2001 | | AS | | -Duration=0  -Nº=0  0 | | | -C  0 | | | - sex=0  - age=0  -any other=0  0 | | | Not specified  0 | | | All pregnant women attending two hospitals  1 | | | | **1** | | |
| **16** | | Linneberg 2003 | | AS | | -Duration=1  -Nº=1  1 | | | - A &  - C  1 | | | - sex=1  - age=1  -any other=1  1 | | | 77.5%  0 | | | 1 | | | | **4** | | |
| **17** | | Montnemery 2003 | | AS | | -Duration=0  -Nº=1  1 | | | - C  0 | | | - sex=0  - age=0  -any other=0  0 | | | 70.1%  0 | | | 1 | | | | **2** | | |
| **18** | | Kramer 2004 | | SHS | | -Duration=0  -Nº=1  - CCR  1 | | | -A  1 | | | - sex=0  - age=0  -any other=0  0 | | | 80%  1 | | | Children registered for admission to school in one year  1 | | | | **4** | | |
| **19** | | Demir 2004 | | SHS | | -Duration=0  -Nº=0  0 | | | - C  0 | | | - sex=0  - age=0  -any other=0  0 | | | 58,4%  0 | | | 1 school  0 | | | | **0** | | |
| Nº | | **Author (Year)** | **OR calculated** | | | **1. Smoking well defined**  - Duration “or”  -Nº | | | **2. DERM. diagnosis well defined**  -A diagnosis (1)  - B,C auto diagnosis)0) | | | **3. Adjusted for:**  - Sex  - Age  - Any other | | | **4. Participation rate ≥80%** | | | **5. Clearly defined target population** | | | | **TOTAL**  **(MAX 5)** | | |
| **20** | | A-Maesano 2004  ISAACq | | AS  SHS | | -Duration=0  -Nº=1  1 | | | - C  0 | | | - sex=1  - age=1  -any other=0  0 | | | 78,8%  0 | | | School children from five different centers  1 | | | | **2** | | |
| **21** | | Miyake 2004  ISAACq | | SHS | | -Duration=0  -Nº=1  1 | | | -C  0 | | | - sex=1  - age=1  -any other=1  1 | | | 61,5%  0 | | | All 18  public junior high schools in Suita City  1 | | | | **2** | | |
| **22** | | Yemaneberhan 2004 | | AS  SHS | | -Duration=0  -Nº=0  0 | | | - C  0 | | | - sex=1  - age=1  -any other=1  1 | | | Not specified  0 | | | 1 | | | | **2** | | |
| **23** | | Lee S-L 2004  ISAAC | | SHS in utero | | -Duration=0  -Nº=0  0 | | | - C  0 | | | - sex=1  - age=1  -any other=1  1 | | | 95%  1 | | | Random selected schools in Hong Kong  1 | | | | **3** | | |
| **24** | | Heudorf 2005  ISAACq | | SHS | | -Duration=0  -Nº=0  0 | | | - C  0 | | | - sex=0  - age=1  -any other=0  0 | | | 82,7%  1 | | | 1 | | | | **2** | | |
| **25** | | Miyake 2005 | | AS  SHS  (women) | | -Duration=0  -Nº=1  1 | | | - C  0 | | | - sex=1  - age= 1  -any other=1  1 | | | 17,2%  0 | | | Women, who become pregnant in one city during 3 years  1 | | | | **3** | | |
| **26** | | Montnemery 2005 | | AS | | -Duration=0  -Nº=1  1 | | | - C  0 | | | - sex=0  - age=0  -any other=0  0 | | | 51.2%  0 | | | 1 | | | | **2** | | |
| **27** | | Obihara 2005  ISAACq | | SHS in Utero | | -Duration=0  -Nº=0  0 | | | -C  0 | | | - sex=1  - age=1  -any other=1  1 | | | 88.0%  1 | | | 1 | | | | **3** | | |
| **28** | | Kurosaka 2006  ISAACq. | | SHS | | -Duration=0  -Nº=0  0 | | | - B  0 | | | - sex=0  - age=1  -any other=0  0 | | | 99.1%  1 | | | 1 | | | | **2** | | |
| Nº | | **Author (Year)** | **OR calculated** | | | **1. Smoking well defined**  - Duration “or”  -Nº | | | **2. DERM. diagnosis well defined**  -A diagnosis (1)  - B,C auto diagnosis)0) | | | **3. Adjusted for:**  - Sex  - Age  - Any other | | | **4. Participation rate ≥80%** | | | **5. Clearly defined target population** | | | | **TOTAL**  **(MAX 5)** | | |
| 29 | | Sakar 2006 | | AS | | -Duration=0  -Nº=0  0 | | | - C  0 | | | - sex=1  - age=1  -any other=1  1 | | | 88.8%  1 | | | Household records of the 9 health centers of Manisa city  1 | | | | **3** | | |
| **30** | | Dotterud 2007 | | AS | | -Duration=0  -Nº=0  0 | | | - A (contact sensitization - patch testing)  1 | | | - sex=1  - age=1  -any other=1  1 | | | 20.0%  0 | | | 1 | | | | **3** | | |
| **31** | | Horak 2007  ISAACq | | SHS  SHS in Utero | | -Duration=0  -Nº=0  0 | | | -B &  - C  0 | | | - sex=1  - age=1  -any other=1  1 | | | 42%  0 | | | Randomly selected 100 kinder gardens (out of 435 in Tyrol(Austria)  1 | | | | **2** | | |
| **32** | | Tanaka 2007 | | SHS | | -Duration=0  -Nº=1  1 | | | - C  0 | | | - sex=1  - age=1  -any other=1  1 | | | 60,3%  0 | | | All schools  1 | | | | **3** | | |
| **33** | | Zuraimi 2008  ISAACq | | SHS | | -Duration=0  -Nº=1  1 | | | - C  0 | | | - sex=0  - age=1  -any other=1  0 | | | 70%  0 | | | Randomly selected child care centers(18%) in Singapore  1 | | | | **2** | | |
| **34** | | Al-Sahab 2008  ISAACq | | SHS | | -Duration=0  -Nº=0  0 | | | - C  0 | | | - sex=1  - age=1  -any other=1  1 | | | 91.8%  1 | | | A random  sample of 55 private and public schools from different regions  in Lebanon  1 | | | | **3** | | |
| **35** | | Ergin 2008 | | SHS | | -Duration=0  -Nº=0  0 | | | - C  0 | | | - sex=0  - age=1  -any other=0  0 | | | 78.9%  0 | | | 1 | | | | **1** | | |
| **36** | | Foliaki 2008  ISAAC | | SHS | | -Duration=0  -Nº=0  0 | | | - C  0 | | | - sex=1  - age=1  -any other=1  1 | | | 78.1%  0 | | | 0 | | | | **1** | | |
| **37** | | Morales Suárez-Varela 2008  ISAACq | | SHS | | -Duration=0  -Nº=1  1 | | | - B  - C  0 | | | - sex=1  - age=1  -any other=1  1 | | | Not specified  0 | | | 1 | | | | **3** | | |
| **38** | | Attwa 2009 | | AS  (men) | | -Duration=0  -Nº=0  0 | | | - A  1 | | | - sex=1  - age=0  -any other=0  0 | | | Not specified  0 | | | 1 | | | | **2** | | |
| Nº | | **Author (Year)** | **OR calculated** | | | **1. Smoking well defined**  - Duration “or”  -Nº | | | **2. DERM. diagnosis well defined**  -A diagnosis (1)  - B,C auto diagnosis)0) | | | **3. Adjusted for:**  - Sex  - Age  - Any other | | | **4. Participation rate ≥80%** | | | **5. Clearly defined target population** | | | | **TOTAL**  **(MAX 5)** | | |
| **39** | | Meding 2009 | | AS | | -Duration=0  -Nº=0  0 | | | C - self reported  0 | | | - sex=1  - age=1  -any other=1  1 | | | 58,1%  0 | | | 1 | | | | **2** | | |
| **40** | | Brescianini 2009  ISAACq | | SHS | | -Duration=0  -Nº=0  0 | | | - C  0 | | | - sex=1  - age=1  -any other=1  1 | | | Not specified  0 | | | Children from 3 schools  0 | | | | **1** | | |
| **41** | | Musharrafieh 2009 ISAACq | | SHS | | -Duration=0  -Nº=0  0 | | | -C  0 | | | - sex=1  - age=1  -any other=1  1 | | | Not specified  0 | | | Random sample of 55 schools and only 13 agreed to participate  1 | | | | **2** | | |
| **42** | | Kabir (2009)  ISAACq | | SHS | | -Duration=0  -Nº=0  0 | | | - C  714)2009)ta)dubl.Keil, but dont it., 0000000000000000000000000000000000000000000000000000000000000000000000000000000000000000  0 | | | - sex=1  - age=1  -any other=0  0 | | | 90%  1 | | | Stratified random sampling of schools  1 | | | | **2** | | |
| **43** | | Lapinska 2009 | | SHS | | -Duration=0  -Nº=0  0 | | | - A  1 | | | - sex=0  - age=0  -any other=0  0 | | | 88.0%  1 | | | 3 randomly selected cities  1 | | | | **3** | | |
| **44** | | Xepapadaki, 2009  ISAAC q | | SHS,  SHS in Utero | | -Duration=1  -Nº=1  1 | | | -C  0 | | | - sex=1  - age=1  -any other=0  0 | | | Not specified  0 | | | 1 | | | | **2** | | |
| **45** | | Wang H-Y 2010  ISAAC | | SHS | | -Duration=0  -Nº=0  0 | | | -B  - C  0 | | | - sex=1  - age=1  -any other=1  1 | | | 97,6%  1 | | | Randomly selected schools from each region  1 | | | | **3** | | |
| **46** | | Rohrl 2010 | | SHS | | -Duration=0  -Nº=0  0 | | | C - self reported  0 | | | - sex=1  - age=0  -any other=1  0 | | | 80,8%  1 | | | 1 | | | | **2** | | |
| **47** | | Thyssen 2010 | |  | | -Duration=0  -Nº=1  1 | | | - C  0 | | | - sex=1  - age=1  -any other=1  1 | | | 44%  0 | | | 1 | | | | 3 | | |
| **48** | | Meding 2010 | | AS | | -Duration=1  -Nº=1  1 | | | - C  0 | | | - sex=1  - age=1  -any other=1  1 | | | 59%  0 | | | Data from a national environmental  health survey  1 | | | | **3** | | |
| Nº | | **Author (Year)** | **OR calculated** | | | **1. Smoking well defined**  - Duration “or”  -Nº | | | **2. DERM. diagnosis well defined**  -A diagnosis (1)  - B,C auto diagnosis)0) | | | **3. Adjusted for:**  - Sex  - Age  - Any other | | | **4. Participation rate ≥80%** | | | **5. Clearly defined target population** | | | | **TOTAL**  **(MAX 5)** | | |
| **49** | | Yang Y-W 2011 | | AS,  SHS | | -Duration=0  -Nº=1  1 | | | - B  0 | | | - sex=0  - age=0  -any other=0  0 | | | 87%  1 | | | the National Health and Nutrition  Examination Survey (NHANES) using a stratified, multistage, cluster  sampling  1 | | | | **3** | | |
| **50** | | Vlaski 2011  ISAAC | | SHS | | -Duration=0  -Nº=0  0 | | | -C  0 | | | - sex=1  - age=0  -any other=1  0 | | | 90.9%  1 | | | 17 randomly selected schools in the region  1 | | | | **2** | | |
| **51** | | Civelek 2011  ISAACq | | SHS  & SHS in Utero | | -Duration=0  -Nº=0  0 | | | - A &  - B  - C  1 | | | - sex=0  - age=1  -any other=0  0 | | | 88.6%  1 | | | group of randomly selected  schoolchildren representative of their peers and residing in each  of 5 cities in Turkey  1 | | | | **3** | | |
| **52** | | Dei-Cas 2011 | | SHS | | -Duration=0  -Nº=0  0 | | | -A  1 | | | - sex=0  - age=1  -any other=0  0 | | | 81%  1 | | | 1 | | | | **3** | | |
| **53** | | Apfelbacher 2011 | | SHS  SHS in Utero | | -Duration=0  -Nº=0  0 | | | - B  0 | | | - sex=1  - age=1  -any other=1  1 | | | 65.2%  0 | | | 1 | | | | **2** | | |
| **54** | | Park 2011 | | As | | -Duration=1  -Nº=0  1 | | | -B  0 | | | - sex=1  - age=1  -any other=1  1 | | | 70%  0 | | | stratified random sampling of census blocks from adult population  0 | | | | **2** | | |
| **55** | | Berglind 2011 | | AS | | -Duration=0  -Nº=0  0 | | | - C  0 | | | - sex=0  - age=0  -any other=1  0 | | | 58.0%  1 | | | Randomly chosen individuals from population register  1 | | | | **2** | | |
| **56** | | Breunig 2012 | | AS  Men only | | -Duration=0  -Nº=0  0 | | | - A  1 | | | - sex=1  - age=1  -any other=1  1 | | | 97.2%  1 | | | Almost all 18-year boys in one city  1 | | | | 4 | | |
| **57** | | Yi 2012 | | SHS,  SHS in utero | | -Duration=0  -Nº=0  0 | | | - B  - C  0 | | | - sex=1  - age=1  -any other=1  1 | | | 76.6%  0 | | | 10 representative cities in Korea  1 | | | | **2** | | |
| **58** | | Ronmark 2012 | | AS | | -Duration=0  -Nº=0  0 | | | - C  0 | | | - sex=1  - age=1  -any other=1  1 | | | 62%  0 | | | 1 | | | | **2** | | |
| Nº | | **Author (Year)** | **OR calculated** | | | **1. Smoking well defined**  - Duration “or”  -Nº | | | **2. DERM. diagnosis well defined**  -A diagnosis (1)  - B,C auto diagnosis)0) | | | **3. Adjusted for:**  - Sex  - Age  - Any other | | | **4. Participation rate ≥80%** | | | **5. Clearly defined target population** | | | | **TOTAL**  **(MAX 5)** | | |
| **59** | | Tanaka K 2012 ISAAC q | | AS  Women only | | -Duration=1  -Nº=1  1 | | | -C  0 | | | - sex=1  - age=1  -any other=1  1 | | | 99.2%  1 | | | 423  obstetric hospitals  1 | | | | **4** | | |
| **60** | | Montefort 2012  ISAACIII | | AS  SHS | | -Duration=1  -Nº=1  1 | | | - C  0 | | | - sex=0  - age=1  -any other=0  0 | | | 80% (5-8year)  90% (13-15year)  1 | | | All state primary schools in Malta and Gozo were randomly  Chosen(44+18 schools)  1 | | | | **3** | | |
| **61** | | Mitchell 2012  ISAACIII | | SHS | | -Duration=0  -Nº=1  1 | | | - C  0 | | | - sex=1  - age=1  -any other=1  1 | | | Not specified  0 | | | Random sample of schools  1 | | | | **3** | | |
|  | |  | |  | | -Duration=  -Nº= | | |  | | | - sex=  - age=  -any other= | | |  | | |  | | | |  | | |

**Quality scoring of food allergies studies**

**CRITERIA:**

**1.**

**FOR SMOKING DEFINITION:**

**Duration** – duration of smoking considered

“or”

**Nº** - number of cigarettes smoked (usually per day/per year)

**2.**

**Determination of food allergy well defined, with one of these:**

A (1) – a physician had diagnosed Food allergy (with at least one of the tests performed: SPT, IgE or open-challenge test.)

B (0) – questionnaire information: a physician had ever diagnosed this condition?

C (0) – self reported food allergies by a questionnaire.

**3.**

Adjusted for: - Sex, Age and Any other.

**4.**

Participation rate ≥ 80%

**5.**

Clearly defined target population

**Abbreviations:**

**AS – active smoking**

**SHS – second hand smoking**

**SHS in Utero – mothers smoking during pregnancy**

SHS + in Utero – second hand smoking + mothers smoking during pregnancy

| Nº | **Author (Year)** | **OR calculated** | | **1. Smoking well defined**  - Duration “or”  -Nº | **2. diagnosis well defined**  -A diagnosis (1)  - B, C (auto diagnosis) (0) | **3. Adjusted for:**  - Sex  - Age  - Any other | **4. Participation rate ≥80%** | **5. Clearly defined target population** | **TOTAL**  **(MAX 5)** |
| --- | --- | --- | --- | --- | --- | --- | --- | --- | --- |
|  | **I. Case-control studies** | | | | | | | | |
| 1 | Metsala J.(Milk) 2010 | SHS in Utero | | -Duration=0  -Nº=0  0 | - A  1 | - sex=0  - age=1  -any other=1  0 | 85.0%  1 | 4 national registers  1 | **3** |
| 2 | Kavaliunas 2011**^A^**  (EuroPrevall) |  | | -Duration=0  -Nº=0  0 | - A (children)  1 | - sex=0  - age=1  -any other=0  0 | 31.3%  0 | 1 | **2** |
|  | Kavaliunas 2011 **^B^**  (EuroPrevall) |  | | -Duration=0  -Nº=0  0 | - A (adult)  1 | - sex=0  - age=0  -any other=0  0 | 22.0%  0 | 1 | **2** |
|  |  |  | | -Duration=  -Nº= |  | - sex=  - age=  -any other= |  |  |  |
|  |  |  | |  |  |  |  |  |  |
|  |  |  | |  |  |  |  |  |  |
|  |  |  | |  |  |  |  |  |  |
|  |  |  | |  |  |  |  |  |  |
|  | **II. Cohort studies** | | | | | | | | |
| Nº | **Author (Year)** | **OR calculated** | | **1. Smoking well defined**  - Duration “or”  -Nº | **2. diagnosis well defined**  -A diagnosis (1)  - B, C (auto diagnosis) (0) | **3. Adjusted for:**  - Sex  - Age  - Any other | **4. Participation rate ≥80%** | **5. Clearly defined target population** | **TOTAL**  **(MAX 5)** |
| 1 | Zeiger RS (1995) | SHS | | -Duration=0  -Nº=0  0 | - A  1 | - sex=0  - age=1  -any other=0  0 | 57%  0 | 0 | **1** |
| 2 | Tariq SM (1998) | SHS | | -Duration=1  -Nº=0  1 | - A?  - C  1 | - sex=0  - age=1  -any other=0  0 | 83,6%  **1** | All infants during certain period  **1** | **4** |
| 3 | Kulig M(1999) | SHS alone &  SHS + SHS In Utero | | -Duration=0  -Nº=1  1 | - A  1 | - sex=0  - age=1  -any other=1  0 | 27.4%  0 | Children from German Multicenter Allergy Study  1 | **3** |
| 4 | NoakesP 2007 | SHS + SHS in utero | | -Duration=0  -Nº=0  0 | - A  1 | - sex=0  - age=1  -any other=0  0 | 67,2 %  0 | 1 | **2** |
| 5 | Lannero E(2007) | SHS  In Utero  SHS+In Utero | | -Duration=1  -Nº=0  1 | - A  1 | - sex=0  - age=1  -any other=1  0 | 75% of all eligible children,  91% at 4-years  0 | 1 | **3** |
|  |  |  | | -Duration=  -Nº= |  | - sex=  - age=  -any other= |  |  |  |
|  |  |  | |  |  |  |  |  |  |
|  |  |  | |  |  |  |  |  |  |
|  | **III. Cross-Sectional Studies** | | | | | | | | |
| Nº | **Author (Year)** | **OR calculated** | | **1. Smoking well defined**  - Duration “or”  -Nº | **2. diagnosis well defined**  -A diagnosis (1)  - B, C (auto diagnosis) (0) | **3. Adjusted for:**  - Sex  - Age  - Any other | **4. Participation rate ≥80%** | **5. Clearly defined target population** | **TOTAL**  **(MAX 5)** |
| 1 | Hjern A (2001) | | SHS | -Duration=0  -Nº=1  1 | - C  0 | - sex=1  - age=1  -any other=1  1 | 80%  1 | Simple random sample  1 | 4 |
| 2 | Dubakiene 2008 | | AS | -Duration=0  -Nº=0  0 | - C  0 | - sex=0  - age=1  -any other=0  0 | 76.1%  0 | 0 | 0 |
|  |  | |  | -Duration=  -Nº= |  | - sex=  - age=  -any other= |  |  |  |
|  |  | |  |  |  |  |  |  |  |
