# Supplementary material for: Active or Passive Exposure to Tobacco Smoking and Allergic Rhinitis, Allergic Dermatitis, and Food Allergy in Adults and Children: A Systematic Review and Meta-Analysis
Source: PLoS Med. 2014 Mar 11;11(3):e1001611. doi: 10.1371/journal.pmed.1001611 (PMC3949681; doi:10.1371/journal.pmed.1001611)
Supplement: Table S2 — Pooled relative risks and 95% confidence intervals of criterion 1 of the quality scale, region of the world, and allergic rhinitis and dermatitis. (DOC) [file pmed.1001611.s002.doc]

### SI 2. Pooled relative risks (RR) and 95% confidence intervals (CI) of criterion 1 of the quality scale, region of the world and allergic rhinitis and dermatitis

**Number of studies RR (95% CI) Random effects Ri* (95%CI) Q test (p value)**

**Active smoking**

Rhinitis Criterion 1 = 0 19 1.04 (0.88-1.23) 0.96 (0.90-0.99) 0.00001

Rhinitis Criterion 1 = 1 15 1.00 (0.88-1.12) 0.88 (0.47-1.00) 0.00001

Dermatitis Criterion 1 = 0 14 1.17 (1.07-1.29) 0.98 (0.59-1.00) 0.00001

Dermatitis Criterion 1 = 1 19 1.25 (1.14-1.36) 0.79 (0.91-1.00) 0.00001

Rhinitis European studies 19 0.97 (0.84-1.13) 0.97 (0.66-1.00) 0.00001

Rhinitis non European studies 15 1.11 (0.94-1.30) 0.85 (0.92-1.00) 0.00001

Dermatitis European studies 21 1.23 (1.15-1.32) 0.97 (0.83-0.99) 0.00001

Dermatitis non European studies 12 1.21 (0.99-1.48) 0.72 (0.66-0.97) 0.0001

#### Secondhand smoke

Rhinitis Criterion 1 = 0 36 1.09 (1.02-1.16) 0.87 (0.90-0.99) 0.00001

Rhinitis Criterion 1 = 1 27 1.11 (1.05-1.18) 0.89 (0.47-1.00) 0.00001

Dermatitis Criterion 1 = 0 40 1.07 (0.99-1.14) 0.87 (0.59-1.00) 0.00001

Dermatitis Criterion 1 = 1 18 1.08 (1.03-1.13) 0.75 (0.91-1.00) 0.00001

Rhinitis European studies 31 1.06 (1.00-1.13) 0.82 (0.66-1.00) 0.00001

Rhinitis non European studies 30 1.15 (1.06-1.23) 0.85 (0.92-1.00) 0.00001

Dermatitis European studies 31 1.02 (0.96-1.09) 0.82 (0.83-0.99) 0.00001

Dermatitis non European studies 26 1.13 (1.06-1.22) 0.79 (0.66-0.97) 0.00001

Criterion1: Assessment of the smoking habit included duration and/or quantity (1) or else (0)

* Proportion of total variance due to between-study variance
