# Supplementary material for: Active or Passive Exposure to Tobacco Smoking and Allergic Rhinitis, Allergic Dermatitis, and Food Allergy in Adults and Children: A Systematic Review and Meta-Analysis
Source: PLoS Med. 2014 Mar 11;11(3):e1001611. doi: 10.1371/journal.pmed.1001611 (PMC3949681; doi:10.1371/journal.pmed.1001611)
Supplement: Table S3 — Results of heterogeneity statistics Ri and I2 for subgroups of active and passive smoking. (DOC) [file pmed.1001611.s003.doc]

Results of heterogeneity statistics Ri and I^2^ for subgroups of active smoking (AS) and secondhand smoke (SHS)

| **Subgroup** | **I^2^** | **Ri** |
| --- | --- | --- |
| Rhinitis AS All studies | 0.94 | 0.95 |
| Rhinitis AS Cohort studies | 0.79 | 0.82 |
| Rhinitis AS Case-control studies | 0.86 | 0.88 |
| Rhinitis AS Cross-sectional studies | 0.95 | 0.95 |
| Rhinitis AS Incidence studies | 0.84 | 0.87 |
| Rhinitis AS Full adjustment | 0.96 | 0.96 |
| Rhinitis AS Incomplete adjustment | 0.91 | 0.91 |
| Rhinitis AS Adults only | 0.81 | 0.82 |
| Rhinitis AS Children/adolescents only | 0.87 | 0.90 |
| Rhinitis AS Children ISAAC method | 0.78 | 0.85 |
| Rhinitis AS Children non-ISAAC method | 0.00 | 0.00 |
| Rhinitis AS Quality score ≥ 3 | 0.85 | 0.86 |
| Rhinitis AS Quality score < 3 | 0.94 | 0.96 |
| Rhinitis SHS All studies | 0.84 | 0.87 |
| Rhinitis SHS Cohort studies | 0.89 | 0.90 |
| Rhinitis SHS Case-control studies | 0.94 | 0.95 |
| Rhinitis SHS Cross-sectional studies | 0.82 | 0.86 |
| Rhinitis SHS Incidence studies | 0.90 | 0.91 |
| Rhinitis SHS Full adjustment | 0.82 | 0.86 |
| Rhinitis SHS Incomplete adjustment | 0.85 | 0.86 |
| Rhinitis SHS Adults only | 0.73 | 0.74 |
| Rhinitis SHS Children/adolescents only | 0.85 | 0.89 |
| Rhinitis SHS Children ISAAC method | 0.78 | 0.84 |
| Rhinitis SHS Children non-ISAAC method | 0.88 | 0.89 |
| Rhinitis SHS Maternal pregnancy smoking | 0.80 | 0.83 |
| Rhinitis SHS Quality score ≥ 3 | 0.79 | 0.86 |
| Rhinitis SHS Quality score < 3 | 0.87 | 0.88 |
| Dermatitis AS All studies | 0.87 | 0.96 |
| Dermatitis AS Cohort studies | undetermined | 0.10 |
| Dermatitis AS Case-control studies | 0.71 | 0.73 |
| Dermatitis AS Cross-sectional studies | 0.88 | 0.97 |
| Dermatitis AS Incidence studies | 0.57 | 0.67 |
| Dermatitis AS Full adjustment | 0.90 | 0.98 |
| Dermatitis AS Incomplete adjustment | 0.76 | 0.77 |
| Dermatitis AS Adults only | 0.84 | 0.96 |
| Dermatitis AS Children/adolescents only | 0.74 | 0.76 |
| Dermatitis AS Quality score ≥ 3 | 0.74 | 0.78 |
| Dermatitis AS Quality score < 3 | 0.89 | 0.98 |
| Dermatitis SHS All studies | 0.83 | 0.84 |
| Dermatitis SHS Cohort studies | 0.78 | 0.89 |
| Dermatitis SHS Case-control studies | 0.27 | 0.34 |
| Dermatitis SHS Cross-sectional studies | 0.78 | 0.81 |
| Dermatitis SHS Incidence studies | 0.75 | 0.86 |
| Dermatitis SHS Full adjustment | 0.77 | 0.81 |
| Dermatitis SHS Incomplete adjustment | 0.80 | 0.84 |
| Dermatitis SHS Adults only | 0.16 | 0.17 |
| Dermatitis SHS Children/adolescents only | 0.83 | 0.85 |
| Dermatitis SHS Children ISAAC method | 0.67 | 0.73 |
| Dermatitis SHS Children non-ISAAC method | 0.87 | 0.89 |
| Dermatitis SHS Maternal pregnancy smoking | 0.79 | 0.80 |
| Dermatitis SHS Quality score ≥ 3 | 0.86 | 0.88 |
| Dermatitis SHS Quality score < 3 | 0.78 | 0.80 |
| Food SHS all | 0.57 | 0.68 |
| Food SHS cohort | 0.00 | 0.01 |
| Food maternal pregnancy | 0.68 | 0.96 |

Note: Ri, the proportion of total variance due to between-study variance, was first proposed in 1999 (Takkouche B, Cadarso-Suarez C, Spiegelman D. Evaluation of old and new tests of heterogeneity in epidemiologic meta-analysis. Am J Epidemiol 1999; 150: 206-15). Later, I^2^ used a slightly different estimate of the within-study variance to compute this proportion (Higgins JPT, Thompson SG. Quantifying heterogeneity in a meta-analysis. Stat Med 2002; 21: 1539-58).
