# Supplementary material for: Correction: Active or Passive Exposure to Tobacco Smoking and Allergic Rhinitis, Allergic Dermatitis, and Food Allergy in Adults and Children: A Systematic Review and Meta-Analysis
Source: PLoS Med. 2016 Feb 2;13(2):e1001939. doi: 10.1371/journal.pmed.1001939 (PMC4737496; doi:10.1371/journal.pmed.1001939)
Supplement: S2 File — (PDF) [file pmed.1001939.s002.pdf]

OPEN ACCESS Freely available online
PLOS | MEDICINE

Active or Passive Exposure to Tobacco Smoking and Allergic Rhinitis, Allergic Dermatitis, and Food Allergy in Adults and Children: A Systematic Review and Meta-Analysis

Jurgita Saulyte[1,2], Carlos Regueira[1,2], Agustín Montes-Martínez[1,2], Polyna Khudyakov[3], Bahi Takkouche[1,2]*

1 Department of Preventive Medicine, University of Santiago de Compostela, Santiago de Compostela, Spain, 2 Centro de Investigación Biomédica en Red de Epidemiología y Salud Pública (CIBER-ESP), Barcelona, Spain, 3 Departments of Epidemiology and Biostatistics, Harvard School of Public Health, Boston, Massachusetts, United States of America

Abstract

Background: Allergic rhinitis, allergic dermatitis, and food allergy are extremely common diseases, especially among children, and are frequently associated to each other and to asthma. Smoking is a potential risk factor for these conditions, but so far, results from individual studies have been conflicting. The objective of this study was to examine the evidence for an association between active smoking (AS) or passive exposure to secondhand smoke and allergic conditions.

Methods and Findings: We retrieved studies published in any language up to June 30th, 2013 by systematically searching Medline, Embase, the five regional bibliographic databases of the World Health Organization, and ISI-Proceedings databases, by manually examining the references of the original articles and reviews retrieved, and by establishing personal contact with clinical researchers. We included cohort, case-control, and cross-sectional studies reporting odds ratio (OR) or relative risk (RR) estimates and confidence intervals of smoking and allergic conditions, first among the general population and then among children. We retrieved 97 studies on allergic rhinitis, 91 on allergic dermatitis, and eight on food allergy published in 139 different articles. When all studies were analyzed together (showing random effects model results and pooled ORs expressed as RR), allergic rhinitis was not associated with active smoking (pooled RR, 1.02 [95% CI 0.92–1.15]), but was associated with passive smoking (pooled RR 1.10 [95% CI 1.06–1.15]). Allergic dermatitis was associated with both active (pooled RR, 1.21 [95% CI 1.14–1.29]) and passive smoking (pooled RR, 1.07 [95% CI 1.03–1.12]). In children and adolescent, allergic rhinitis was associated with active (pooled RR, 1.40 (95% CI 1.24–1.59) and passive smoking (pooled RR, 1.09 [95% CI 1.04–1.14]). Allergic dermatitis was associated with active (pooled RR, 1.36 [95% CI 1.17–1.46]) and passive smoking (pooled RR, 1.06 [95% CI 1.01–1.11]). Food allergy was associated with SHS (1.43 [1.12–1.83]) when cohort studies only were examined, but not when all studies were combined. The findings are limited by the potential for confounding and bias given that most of the individual studies used a cross-sectional design. Furthermore, the studies showed a high degree of heterogeneity and the exposure and outcome measures were assessed by self-report, which may increase the potential for misclassification.

Conclusions: We observed very modest associations between smoking and some allergic diseases among adults. Among children and adolescents, both active and passive exposure to SHS were associated with a modest increased risk for allergic diseases, and passive smoking was associated with an increased risk for food allergy. Additional studies with detailed measurement of exposure and better case definition are needed to further explore the role of smoking in allergic diseases.

Please see later in the article for the Editors' Summary.

Citation: Saulyte J, Regueira C, Montes-Martínez A, Khudyakov P, Takkouche B (2014) Active or Passive Exposure to Tobacco Smoking and Allergic Rhinitis, Allergic Dermatitis, and Food Allergy in Adults and Children: A Systematic Review and
?Meta-Analysis. PLoS Med 11(3): e1001611. doi:10.1371/journal.pmed.1001611

Academic Editor: Thomas E. Novotny, San Diego State University, United States of America

Received March 26, 2013; **Accepted** January 22, 2014; **Published** March 11, 2014

Copyright: © 2014 Saulyte et al. This is an open-access article distributed under the terms of the Creative Commons Attribution License, which permits unrestricted use, distribution, and reproduction in any medium, provided the original author and source are credited.

Funding: Grant PI10/01295 of the Institute Carlos III, Spanish Ministry of Health, Madrid, Spain. The funders had no role in study design, data collection and analysis, decision to publish, or preparation of the manuscript.

Competing Interests: The authors have declared that no competing interests exist.

Abbreviations: AS, active smoking; IgE, immunoglobulin-E; ISAAC, International Study of Asthma and Allergies in Childhood; OR, odds ratio; RR, relative risk; SHS, secondhand smoke; SPT, skin prick test.

* E-mail: bahi.takkouche@usc.es

Introduction

Allergic rhinitis, allergic dermatitis, and food allergy, in addition to asthma, are extremely common diseases worldwide. Indeed, allergic rhinitis affects 10% to 20% of the general population in Europe and the US [1,2] and up to 40% of children [3]. The prevalence of allergy to any food varies between 3% and 35% [4], while that of allergic dermatitis reaches 20% in many countries [5]. These diseases have profound consequences on the patient's quality of life and imply a high cost both to the patient and insurance providers [6,7]. Among infants, these costs reach more than US$4,000 per year per case of food allergy [8].

Recent studies have suggested that these diseases are but one unique set of immunoglobulin-E (IgE)-mediated allergic conditions, linked by the common thread of "atopic march" [9]. This concept postulates that those conditions are a continuous state that starts with dermatitis and food allergy and eventually progresses to asthma and allergic rhinitis. Indeed, these diseases often co-exist in the same patient and can predict the occurrence of each other [10].

Worldwide, the prevalence of allergic diseases has increased substantially in the last few decades [11,12], which may have two explanations. On the one hand, increased clinician awareness, as well as patient and parental awareness, may have led to improved identification and increased case presentation to physicians [12]. On the other hand, it is possible that this increase is due to changing exposure to known and unknown risk factors [13], and among these factors, smoking may play a role. An increased risk of allergic diseases among individuals exposed to tobacco smoke is biologically plausible as smoking is known to facilitate sensitization to perennial indoor allergens, such as those caused by furry animals, as well as to some outdoor allergens such as pollen [14].

Increased risk of food allergy among infants exposed to tobacco smoke is also plausible. Food allergens are likely to be found in house dust. Swallowed foods are also inhaled or aspirated by infants, and thus, may cause sensitization that could be facilitated by exposure to tobacco smoke. The early and simultaneous exposure to tobacco smoke and food allergens may interfere with

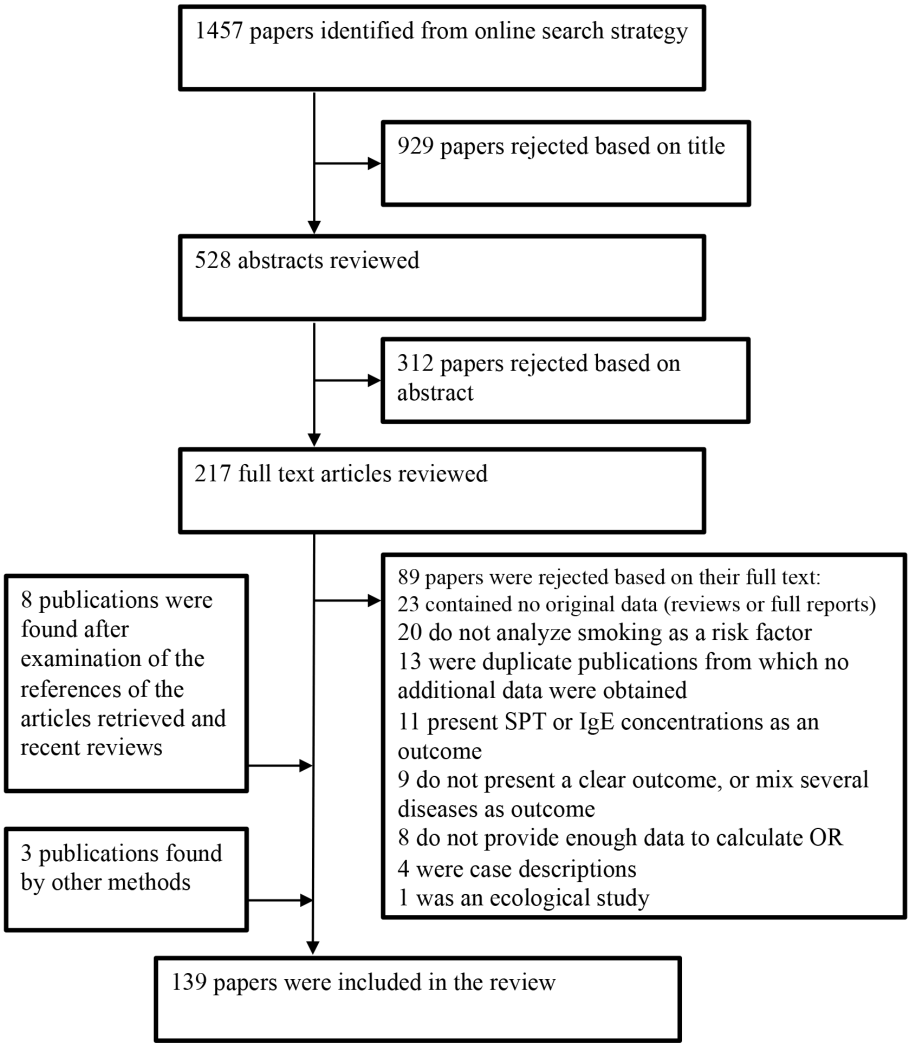

Figure 1. Flow diagram for study selection.
doi:10.1371/journal.pmed.1001611.g001

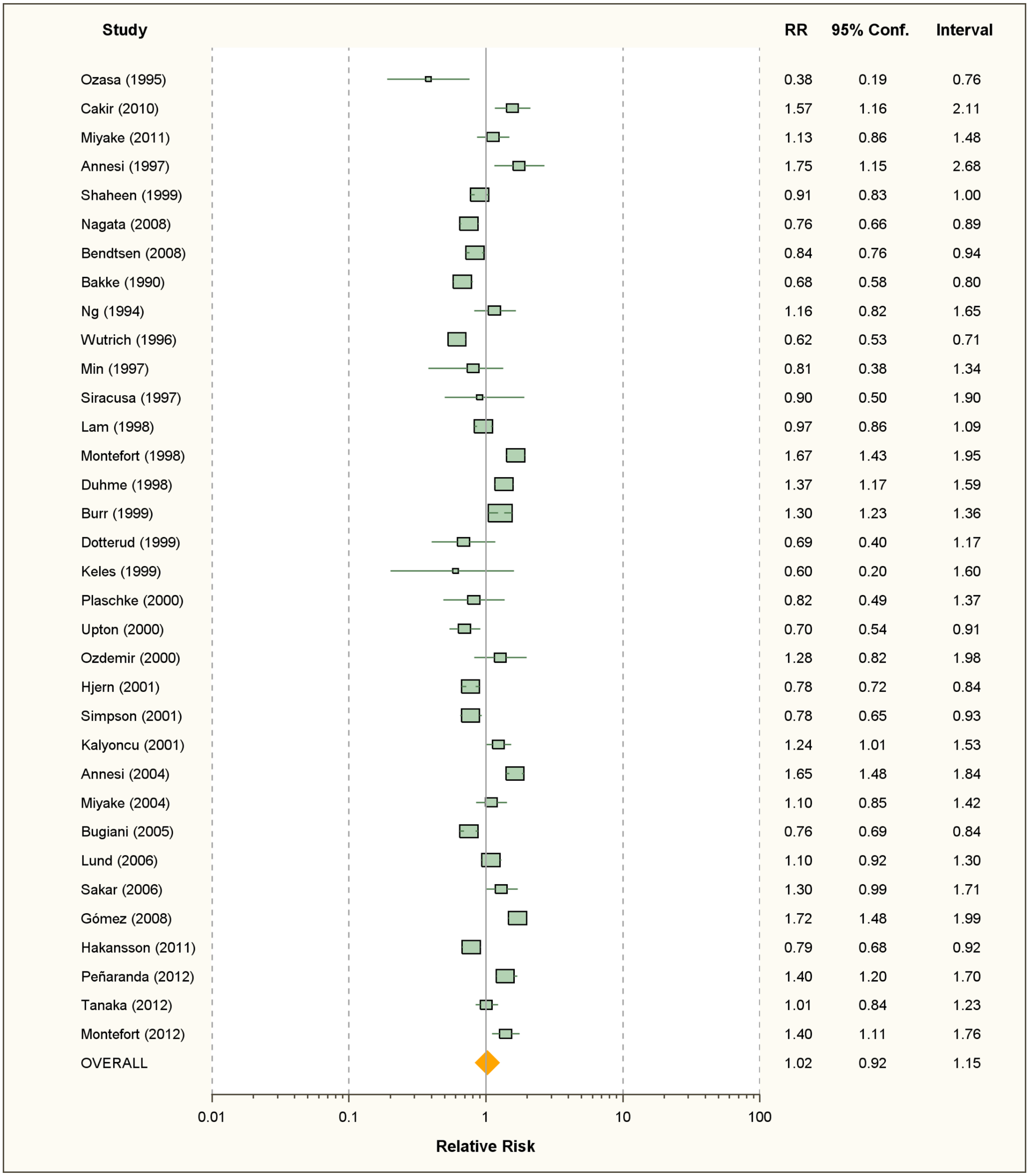

Figure 2. Study-specific and random effects pooled relative risks of active smoking and allergic rhinitis.
doi:10.1371/journal.pmed.1001611.g002

the normal development of immunologic tolerance and thus, facilitate sensitization to food [14].

Furthermore, smoking augments nasal responses to allergen in atopic subjects and increases IgE, immunoglobulin G4 (IgG4), and postallergen histamine levels in nasal lavage fluid [15,16].

Allergic conditions are, in general, more prevalent in children. A potential effect of smoking would have a considerable impact on public health due to the frequency of exposure worldwide. Indeed, children and adolescents are exposed to secondhand smoke in a proportion that varies between 27.6% in Africa and 77.8% in

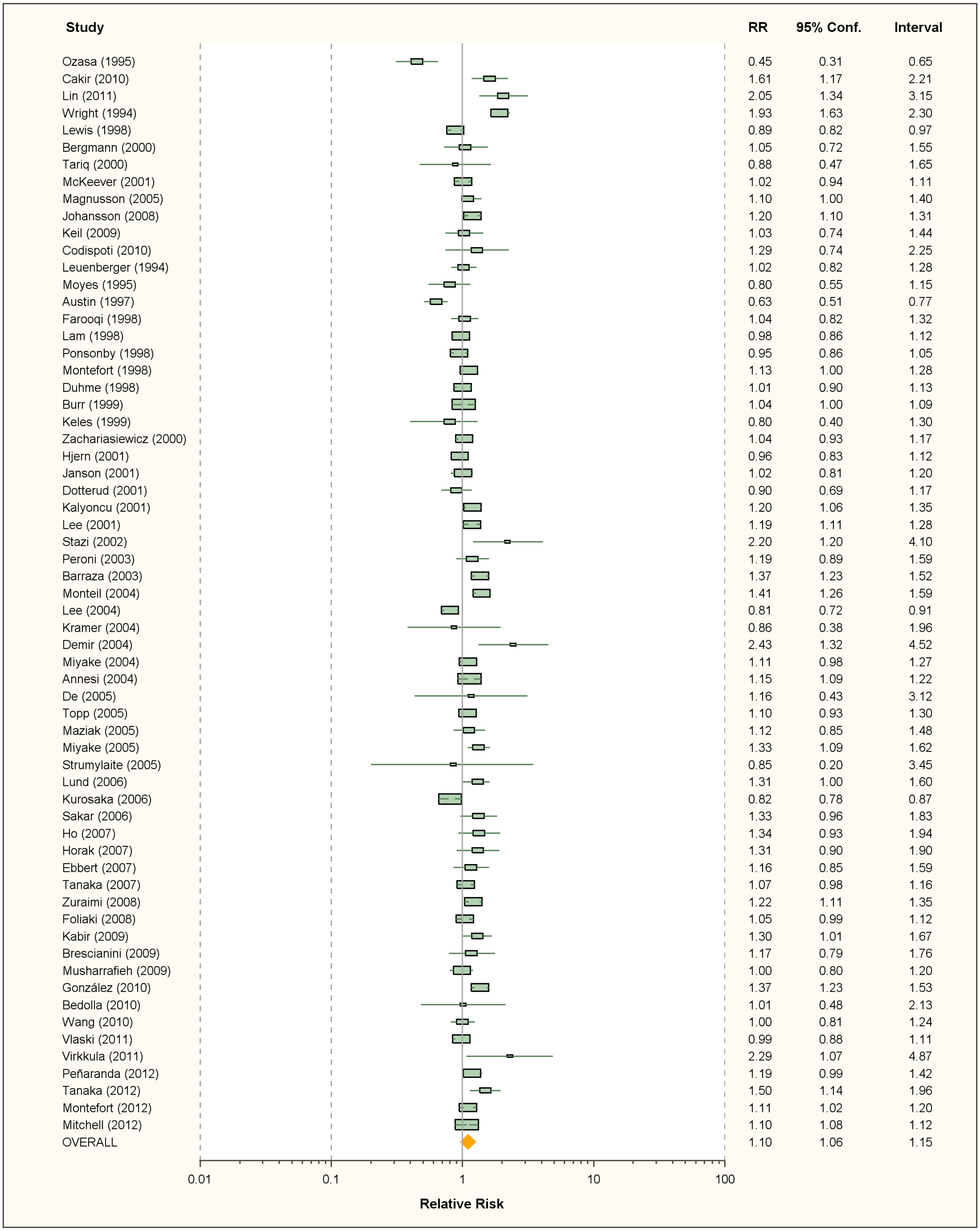

Figure 3. Study-specific and random effects pooled relative risks of passive smoking and allergic rhinitis.
doi:10.1371/journal.pmed.1001611.g003

Table 1. Relative risks and 95% confidence intervals of allergic rhinitis by smoking exposure in case-control and cohort studies.

Source	Country	Population	Follow-up (y)	Complete Follow-up (%)	Active Smoking	Passive Smoking	Cases/Controls or Cohort Size	Variables of Adjustment, Matching, or Restriction
**Case-control studies**								
Ozasa 1995 [39]	Japan	Adults	—	—	0.38 (0.19–0.76)	0.45 (0.31–0.65)	89/89	Age
Cakir 2010 [19]	Turkey	Adolescents	—	—	1.57 (1.16–2.11)	1.61 (1.17–2.21)	436/366	Age, sex, family atopy, pets, income, occupation
Lin 2011 [40]	USA	Adults	—	—	—	2.05 (1.34–3.15)	83/117	Age, sex, education
Miyake 2011 [41]	Japan	Adult women	—	—	1.13 (0.86–1.48)	—	393/767	Sex
**Cohort studies**								
Wright 1994 [42]	USA	Children	6	76.8	—	1.93 (1.63–2.30)	311/747	Not specified
Annesi-Maesano 1997 [43]	France	Adult men	5	49	1.75 (1.15–2.68)	—	126/191	Sex
Lewis 1998 [44]	UK	Children	16	55	—	0.89 (0.82–0.97)	1,646/6,281	Age, sex, social class, low birth weight, gestational age, breast feeding, maternal age, parity
Shaheen 1999 [45]	UK	Young adults	26	51.1	0.91 (0.83–1.00)	—	?/6,420	Age, sex, birth weight, social class, siblings, education, height, body mass index
Bergmann 2000 [46]	Germany	Children	6	75	—	1.05 (0.72–1.55)	178/825	Age, sex, parental atopy, socioeconomic status, breast feeding, aeroallergen and food sensitivity, study center
Tariq 2000 [47]	UK	Children	4	79.3	—	0.88 (0.47–1.65)	65/1218	Age
McKeever 2001 [24]	UK	Children	11	95	—	1.02 (0.94–1.11)	1,113/29,238	Age, sex, family atopy, siblings
Magnusson 2005 [48]	Denmark	Children	18	74	—	1.1 (1.0–1.4)	1,083/7,844	Sex, social class, occupation, maternal age in pregnancy, coffee consumption, parity, breastfeeding
Johansson 2008 [49]	Sweden	Children	3	51.9	—	1.20 (1.10–1.31)	?/8,850	Age, mothers' education, family type
Nagata 2008 [50]	Japan	Adults	10	81.6	0.76 (0.66–0.89)	—	1,000/12,221	Age, sex, marital status, education, body mass index, farming, alcohol
Bendtsen 2008 [21]	Denmark	Adult women	9	87	0.84 (0.76–0.94)	—	1,354/5,870	Age, sex, education, alcohol, parental asthma
Keil 2009 [51]	Germany	Children	10	73	—	1.03 (0.74–1.44)	198/784	Age, sex, birth weight, breast feeding, siblings, pets, parental education, IgE, location
Codispoti 2010 [52]	USA	High risk children	2	?	—	1.29 (0.74–2.25)	116/361	Age, parental allergies

doi:10.1371/journal.pmed.1001611.t001

Table 2. Relative risks and 95% confidence intervals of allergic rhinitis by smoking exposure in cross-sectional studies.

Source	Country	Population	Active Smoking	Passive Smoking	Study Size	Variables of Adjustment, Matching, or Restriction
Bakke 1990 [53]	Norway	Adolescents and adults	0.68 (0.58–0.80)	—	4,270	Age, sex, occupational exposure, residence
Leuenberger 1994 [54]	Switzerland	Adults	—	1.02 (0.82–1.28)	3,494	Not specified
Ng 1994 [55]	Singapore	Adults	1.16 (0.82–1.65)	—	2,868	Age, race, housing, cockroaches, occupation, fumes
Moyes 1995 [56]	New Zealand	Schoolchildren	—	0.80 (0.55–1.15)	5,360	Age
Wutrich 1996 [57]	Switzerland	Adults	0.62 (0.53–0.71)	—	8,344	Age, sex, location
Min 1997 [58]	Korea	Children and adults	0.81 (0.38–1.34)	—	8,853	Age
Siracusa 1997 [59]	Italy	Children and adults	0.9 (0.5–1.9)	—	824	Age, sex, allergens
Austin 1997 [60]	UK	Children	—	0.63 (0.51–0.77)	1,537	Age
Farooqi 1998 [61]	UK	Children	—	1.04 (0.82–1.32)	1,934	Not specified
Lam 1998 [62]	Hong Kong	Schoolchildren	0.97 (0.86–1.09)	0.98 (0.86–1.12)	6,304	Age, sex, residence, housing
Ponsonby 1998 [63]	Australia	Children	—	0.95 (0.86–1.05)	6,378	Age
Montefort 1998 [64]	Malta	Schoolchildren	1.67 (1.43–1.95)	1.13 (1.0–1.28)	4,184	Age, sex, road, pets, parental atopy, blankets
Duhme 1998 [65]	Germany	Schoolchildren	1.37 (1.17–1.59)	1.01 (0.90–1.13)	13,123	Age, sex
Burr 1999 [66]	UK	Schoolchildren	1.30 (1.23–1.36)	1.04 (1.00–1.09)	25,393	Age, sex, location, residence, pets, cooking and heating fuel, housing
Dotterud 1999 [67]	Russia	Adults	0.69 (0.40–1.17)	—	3,368	Not specified
Keles 1999 [68]	Turkey	Adolescents	0.6 (0.2–1.6)	0.8 (0.4–1.3)	386	Age, sex, heating, location
Plaschke 2000 [69]	Sweden	Adults	0.82 (0.49–1.37)	—	1,370	Age, sex, location, pets, allergens
Zacharasiewicz 2000 [70]	Austria	Children	—	1.04 (0.93–1.17)	18,606	Age, sex, family history of hay fever, education
Upton 2000 [71]	UK	Adults	0.70 (0.54–0.91)	—	2,832	Age
Ozdemir 2000 [72]	Turkey	University freshmen	1.28 (0.82–1.98)	—	1,515	Age
Hjern 2001 [73]	Sweden	Children	—	0.96 (0.83–1.12)	4,472	Age, sex, siblings, parental education, residence, single parent household, country of birth of parents, location
Hjern 2001 [73]	Sweden	Adults	0.78 (0.72–0.84)	—	6,909	Age, sex, education, residence, country of birth, location
Janson 2001 [74]	World	Adults	—	1.02 (0.81–1.20)	7,882	Age, sex, allergens, IgE, location
Simpson 2001 [75]	UK	Adults	0.78 (0.65–0.93)	—	5,687	Sex, allergens, pets
Dotterud 2001 [76]	Russia	Schoolchildren	—–	0.90 (0.69–1.17)	1,684	Age, sex, carpets, dampness, pets, heating type
Kalyoncu 2001 [77]	Turkey	University students	1.24 (1.01–1.53)	1.20 (1.06–1.35)	4,639	Age, sex, region, family atopy, pets, elder siblings
Lee 2001 [78]	Korea	Schoolchildren	—	1.19 (1.11–1.28)	38,955	Age, sex, region, body mass index, carpets, pets, location
Stazi 2002 [79]	Italy	Children	—	2.2 (1.2–4.1)	201	Age, sex
Peroni 2003 [80]	Italy	Preschool children	—	1.19 (0.89–1.59)	1,402	Age
Barraza 2003 [81]	Mexico	Schoolchildren	—	1.37 (1.23–1.52)	6,174	Age, school, cockroaches, respiratory problems, use of carpets, humidity, family history of asthma
Monteil 2004 [82]	Trinidad & Tobago	Schoolchildren	—	1.41 (1.26–1.59)	3,170	Age
Lee 2004 [83]	Hong Kong	School children	—	0.81 (0.72–0.91)	4,448	Age, sex, birth weight, siblings, respiratory tract infections, parental atopy, pets, study period

Table 2. Cont.

Source	Country	Population	Active Smoking	Passive Smoking	Study Size	Variables of Adjustment, Matching, or Restriction
Kramer 2004 [84]	Germany	School beginners	—	0.86 (0.38–1.96)	1,220	Age, sex, atopy, nationality
Demir 2004 [85]	Turkey	Schoolchildren	—	2.43 (1.32–4.52)	1,064	Age
Miyake 2004 [86]	Japan	Schoolchildren	—	1.11 (0.98–1.27)	5,539	Age, sex, grade, older siblings, maternal age at child birth, pets, history of other allergic diseases
Annesi-Maesano 2004 [87]	France	Adolescents	1.65 (1.48–1.84)	1.15 (1.09–1.22)	14,578	Age, sex
De 2005 [88]	Ireland	Children	—	1.16 (0.43–3.12)	81	Not specified
Topp 2005 [89]	Germany	Adults	—	1.10 (0.93–1.30)	4,093	Age, sex, social class, location
Maziak 2005 [90]	Syria	Adults	—	1.12 (0.85–1.48)	1,118	Age, sex, familial atopy, socioeconomic status, occupational
Miyake 2005 [91]	Japan	Pregnant women	1.10 (0.85–1.42)	1.33 (1.09–1.62)	1,002	Age, sex, familial atopy, pets, gestation, parity, family income, education, mite antigen level
Bugiani 2005 [92]	Italy	Young adults	0.76 (0.69–0.84)	—	17,666	Not specified
Obihara 2005 [93][a]	South Africa	Children	—	—	861	Age, sex, maternal atopy, breast feeding, siblings, household income, tuberculin test
Strumylaite 2005 [94]	Lithuania	Children	—	0.85 (0.20–3.45)	594	Age
Lund 2006 [95][b]	France	Mature women	1.10 (0.92–1.30)	1.31 (1.0–1.6)	2,197	Age, sex
Kurosaka 2006 [96]	Japan	Schoolchildren	—	0.82 (0.78–0.87)	35,213	Age, sex, pets
Sakar 2006 [97]	Turkey	Adults	1.30 (0.99–1.71)	1.33 (0.96–1.83)	1,336	Age, sex, family atopy
Ho 2007 [98]	Hong Kong	Adults	—	1.34 (0.93–1.94)	200	Age, sex, education, occupational exposures
Horak 2007 [99]	Austria	Preschool children	—	1.31 (0.90–1.90)	1,737	Age, sex, familial atopy, education, family size, pets, breastfeeding, healthy nutrition
Ebbert 2007 [100]	USA	Adults	—	1.16 (0.85–1.59)	1,007	Not specified
Tanaka 2007 [101]	Japan	Children	—	1.07 (0.98–1.16)	23,044	Age, sex, location, familial atopy, siblings, education level
Zuraimi 2008 [102]	Singapore	Preschool children	—	1.22 (1.11–1.35)	4,759	Age, sex, familial atopy, race, socioeconomic status, housing type, breastfeeding, food allergy, respiratory infections, housing conditions, traffic density
Foliaki 2008 [103]	Pacific countries	Children	—	1.05 (0.99–1.12)	17,683	Age, sex, country
Gomez 2008 [104]	Argentina	Adolescents	1.72 (1.48–1.99)	—	3,000	Age
Kabir 2009 [105]	Ireland	Children		1.30 (1.01–1.67)	2,809	Age, sex
Brescianini 2009 [106]	Italy	Schoolchildren	—	1.17 (0.79–1.76)	481	Age, sex, family atopy, body mass index, pets, physical activity, diet, location
Musharrafieh 2009 [107]	Lebanon	Adolescents	—	1.0 (0.8–1.2)	3,115	Age, sex, nationality, regions, school type, traffic
Gonzalez-Diaz 2010 [108]	Mexico	Children and Adolescents	—	1.37 (1.23–1.53)	23,191	Age
Bedolla-Barajas 2010 [109]	Mexico	Schoolchildren	—	1.01 (0.48–2.13)	740	Age
Wang 2010 [110]	Canada	Schoolchildren	—	1.00 (0.81–1.24)	8,334	Age, sex, body mass index, location, birthplace, ethnicity, maternal education, siblings, fuel use, pets, acetaminophen, physical activity

Table 2. Cont.

Source	Country	Population	Active Smoking	Passive Smoking	Study Size	Variables of Adjustment, Matching, or Restriction
Vlaski 2011 [111]	Macedonia	Adolescents	—	0.99 (0.88–1.11)	3,026	Age, sex, diet, type of cooking and heating, pets, maternal education, siblings
Virkkula 2011 [112]	Finland	Children	—	2.29 (1.07–4.87)	38	Age
Hakansson 2011 [113]	Denmark	Adults	0.79 (0.68–0.92)	—	3,471	Age, sex
Chen 2012 [114]	Taiwan	Children	—	—	4,221	Age, sex, parental atopy, parental education
Peñaranda 2012 [115]	Colombia	Children	—	1.19 (0.99–1.42)	3,256	Age, asthma, dermatitis, use of acetaminophen and antibiotics, maternal education, caesarean delivery
Peñaranda 2012 [115]	Colombia	Adolescents	1.4 (1.2–1.7)	—	3,829	Age, asthma, dermatitis, use of acetaminophen, consumption of fast-food, cats
Tanaka 2012 [116]	Japan	Pregnant women	1.01 (0.84–1.23)	1.50 (1.14–1.96)	1,743	Age, sex, region of residence, parental atopy, household income, education
Montefort 2012 [117]	Malta	Children	1.40 (1.11–1.76)	1.11 (1.02–1.20)	7,955	Age
Mitchell 2012 [118]	Multiple countries	Children	—	1.10 (1.08–1.12)	573,061	Age, sex, language, region, gross national income

[a]Only data on maternal smoking during pregnancy are available.
[b]This study used cases of rhinitis at large, not only allergic rhinitis.
doi:10.1371/journal.pmed.1001611.t002

Europe [17] and approximately 14% of all children were exposed to maternal smoking during pregnancy [18].

Several studies have assessed the association between smoking exposure and allergic diseases. In each of the allergic conditions, results were conflicting and alternated between the harmful effects of smoking [14,19,20] and protection [21–23], while some studies could not find evidence of any effect [24–26].

Except for a systematic review and meta-analysis examining the relationship between smoking and asthma in children [27], to our knowledge, there is no comprehensive meta-analysis that examines the evidence for a relationship between smoking and allergic conditions. We, therefore, summarized the scientific evidence and carried out a meta-analysis on exposure to active and passive smoking and the risk of allergic rhinitis, allergic dermatitis, and food allergy among adults and children/adolescents.

Methods

Data Sources and Searches

We searched databases from 1966 to June 30th, 2013, to identify all potentially eligible studies. For Medline, we applied the following algorithm both in medical subject heading and in free text words: ("SEASONAL ALLERGIC RHINITIS" OR "POLLEN ALLERG*" OR "POLLINOSIS" OR "POLLINOSES" OR "HAY FEVER" OR "RHINITIS, ALLERGIC, NONSEASONAL" OR "RHINITIS, ALLERGIC, PERENNIAL" OR "DERMATITIS, ATOPIC" OR ECZEMA OR "FOOD ALLERGIES" OR "HYPERSENSITIVITY, FOOD") AND (SMOKING OR TOBACCO OR CIGARETT*). We used similar strategies to search Embase and the five regional bibliographic databases of the World Health Organization (AIM, LILACS, IMEMR, IMSEAR, WPRIM). We searched meeting abstracts using the ISI Proceedings database from its inception in 1990 to 2013. We also examined the references of every article retrieved and those of recent reviews of allergic rhinitis and smoking [16,28–33] and established personal contact with clinical researchers to trace further publications or reports. We considered including any relevant article, independently of the language of publication.

Study Selection

Studies were included if: (1) they presented original data from cohort, case-control, or cross-sectional studies (ecologic studies were not included); (2) the outcome of interest was clearly defined as allergic rhinitis, allergic dermatitis, or food allergy; (3) one of the exposure factors was smoking, either by the subjects themselves or their relatives; (4) they provided estimates of odds ratio (OR), relative risk (RR), or prevalence odds ratio and their confidence intervals, or enough data to calculate them. If data on the same population were duplicated in more than one study, the most recent study was included in the analysis. When data for different types or levels of exposure were available in the same study, such as passive smoking, active smoking, or maternal smoking during pregnancy, we considered each type of exposure separately. We developed a standard data-recording form in which we recorded authors, year of publication, study location, sample size, outcome, outcome measurement details, effect estimator (OR, RR, other), effect estimate, 95% CIs, adjustment factors used, and study design including if the International Study of Asthma and Allergies in Childhood (ISAAC) methodology was followed. ISAAC is a large international epidemiologic study on risk factors of allergic diseases, the methods of which are widely used. When further clarification was necessary, we attempted to contact the authors. Abstracts were reviewed independently by two authors (BT and JS).

Table 3. Pooled relative risks and 95% confidence intervals of allergic rhinitis and smoking.

Study Type	Number of Studies	RR (95% CI) Fixed Effects	RR (95% CI) Random Effects	Ri[a] (95% CI)	Q test (*p*-Value)
**Active smoking**					
All studies	34	1.06 (1.03–1.08)	1.02 (0.92–1.15)	0.95 (0.90–0.99)	0.00001
Cohort studies	4	0.87 (0.82–0.93)	0.91 (0.77–1.07)	0.82 (0.47–1.00)	0.0024
Case-control studies	3	1.19 (0.99–1.45)	0.97 (0.55–1.70)	0.88 (0.59–1.00)	0.0009
Cross-sectional studies	27	1.09 (1.06–1.12)	1.03 (0.91–1.18)	0.95 (0.91–1.00)	0.00001
Cohort+case-control studies	7	0.90 (0.85–0.96)	0.98 (0.81–1.18)	0.87 (0.66–1.00)	0.00001
Full adjustment	18	1.07 (1.04–1.10)	1.02 (0.88–1.20)	0.96 (0.92–1.00)	0.00001
Incomplete adjustment	16	1.03 (0.98–1.08)	1.02 (0.86–1.22)	0.91 (0.83–0.99)	0.00001
Adults only	21	0.84 (0. 81–0.87)	0.90 (0.82–0.99)	0.82 (0.66–0.97)	0.00001
Children/adolescents only	10	1.35 (1.30–1.39)	1.40 (1.24–1.59)	0.90 (0.77–1.00)	0.00001
Children ISAAC method	8	1.39 (1.34–1.44)	1.50 (1.35–1.66)	0.85 (0.63–1.00)	0.00001
Children non-ISAAC method	2	0.96 (0.86–1.08)	0.96 (0.86–1.08)	0.00 (0.00–1.00)	0.34
Quality score ≥3	15	0.89 (0.86–0.93)	0.95 (0.85–1.06)	0.86 (0.73–0.99)	0.00001
Quality score <3	19	1.19 (1.16–1.23)	1.09 (0.92–1.29)	0.96 (0.91–1.00)	0.00001
Passive Smoking					
All studies	63	1.08 (1.07–1.10)	1.10 (1.06–1.15)	0.87 (0.75–0.99)	0.00001
Cohort studies	9	1.08 (1.03–1.13)	1.14 (0.96–1.34)	0.90 (0.76–1.00)	0.00001
Case-control studies	3	1.13 (0.91–1.39)	1.14 (0.46–2.82)	0.95 (0.84–1.00)	0.00001
Cross-sectional studies	51	1.08 (1.07–1.10)	1.09 (1.05–1.14)	0.86 (0.72–0.99)	0.00001
Cohort+case-control studies	12	1.08 (1.03–1.13)	1.13 (0.96–1.34)	0.91 (0.79–1.00)	0.00001
Full adjustment	37	1.07 (1.06–1.09)	1.07 (1.03–1.12)	0.86 (0.72–1.00)	0.00001
Incomplete adjustment	26	1.17 (1.13–1.20)	1.15 (1.04–1.27)	0.86 (0.74–0.97)	0.00001
Adults only	13	1.17 (1.10–1.24)	1.17 (1.03–1.32)	0.74 (0.50–0.98)	0.00001
Children/adolescents only	50	1.08 (1.07–1.09)	1.09 (1.04–1.14)	0.89 (0.77–0.99)	0.00001
Children ISAAC method	28	1.10 (1.09–1.12)	1.11 (1.07–1.16)	0.84 (0.66–1.00)	0.00001
Children non-ISAAC method	21	0.98 (0.95–1.01)	1.06 (0.95–1.19)	0.89 (0.78–1.00)	0.00001
Maternal pregnancy smoking	11	1.01 (0.96–1.06)	1.07 (0.92–1.28)	0.83 (0.60–1.00)	0.00001
Quality score ≥3	30	1.09 (1.08–1.11)	1.10 (1.04–1.15)	0.86 (0.71–1.00)	0.00001
Quality score <3	33	1.07 (1.04–1.09)	1.10 (1.02–1.19)	0.88 (0.78–0.98)	0.00001

[a]Proportion of total variance due to between-study variance.
doi:10.1371/journal.pmed.1001611.t003

Quality Assessment

Study quality was assessed using a five-point binary scale specifically developed for this study. The scale is based on the Newcastle-Ottawa scale [34] with modifications in view of standard guidelines and our own judgment. The Newcastle-Ottawa scale is a scoring system that assesses every aspect of an observational epidemiologic study from a methodological point of view. For this meta-analysis, we tried to use those elements that were common to all epidemiologic designs and thus shortened the scale considerably. We used the following criteria labelled as "yes" or "no": (1) whether assessment of the smoking habit included duration and/or quantity (yes) or not (no); (2) whether rhinitis diagnosis included clinical features and IgE or skin prick test (SPT) measurements (yes) or was based on clinical examination or questionnaire only (no), whether dermatitis diagnosis included clinically assessed diagnosis (yes) or was based on questionnaire information only (no), whether the diagnosis of food allergy was based on clinical diagnosis with SPT, IgE, or open-challenge test (yes) or was based on questionnaire information only (no); (3) whether results were adjusted for age, sex, and at least one other potential confounder (yes) or not (no); (4) whether participation exceeded 80% of the people initially approached (yes) or not (no); and, finally (5) whether the target population was clearly defined (yes) or, on the contrary, based on convenience sampling of subjects such as patients of a single consultation (no). Throughout this assessment, when the information on a specific item was not provided by the authors, we graded this item as "no." We carried out a pooled analysis on those studies that fulfilled at least three criteria and compared with those that scored fewer than three. As a secondary analysis, we stratified our results on criterion 1 and present the pooled relative risks in Table S2.

Data extraction and quality scoring were performed independently by two reviewers (BT and JS) and the results were merged by consensus. The complete protocol and results for quality scoring are available in Table S1.

Data Synthesis and Analysis

We weighted the study-specific log odds ratios for case control and cross-sectional studies, and log relative risks for cohort studies by the inverse of their variance to compute a pooled relative risk

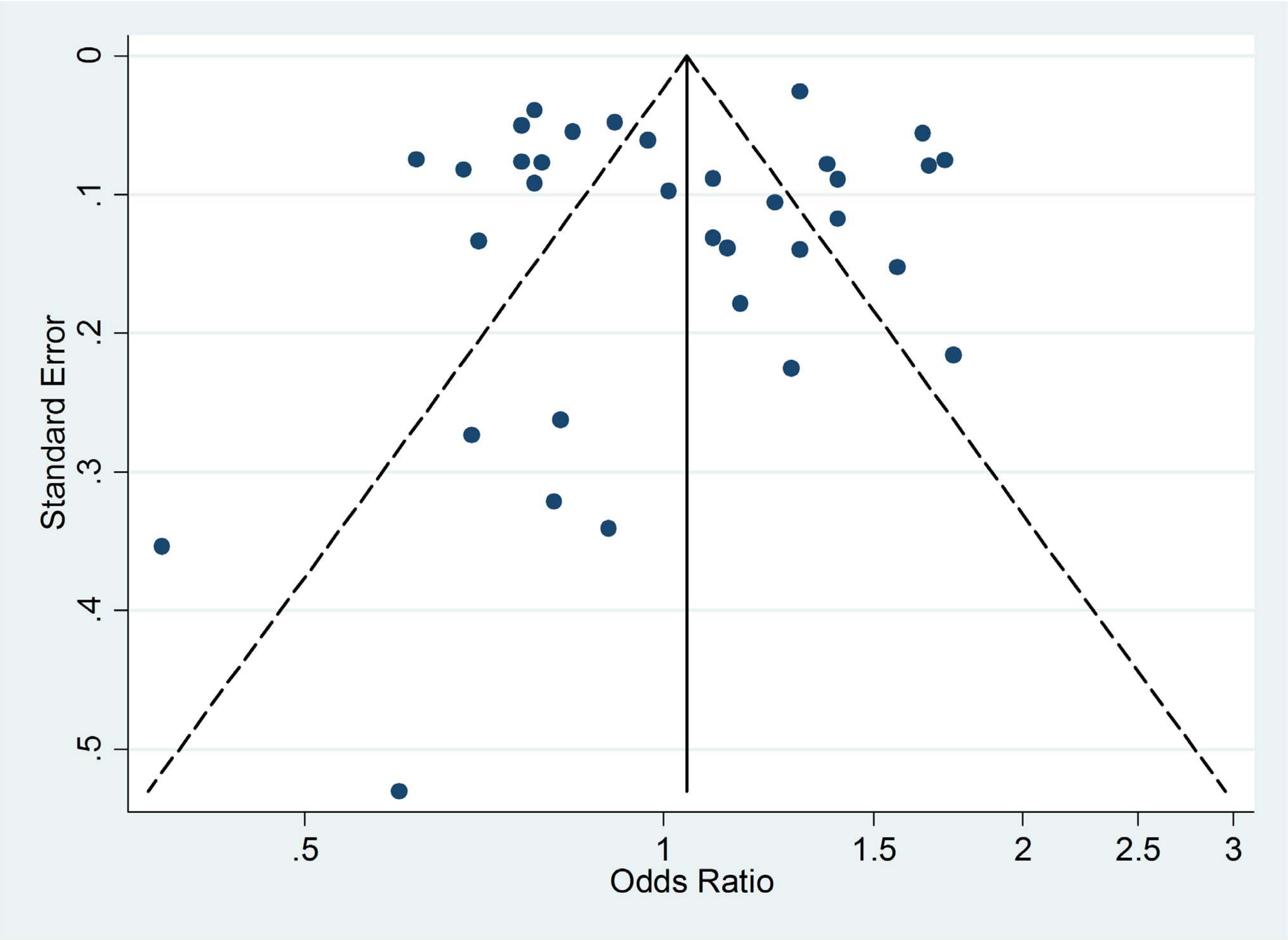

Figure 4. Funnel plot of relative risk versus standard error of relative risk: allergic rhinitis, active smoking.
doi:10.1371/journal.pmed.1001611.g004

and its 95% confidence interval. For each study, we used the estimate of the effect measure that was adjusted for the largest number of confounders. We present both fixed-effects and random effects pooled estimates but use the latter when heterogeneity was present. Odds ratios from case-control studies were assumed to be unbiased estimates of the relative risk [35].

We used a version adapted to small samples of the DerSimonian and Laird Q test to check for heterogeneity [36]. The null hypothesis of this test is the absence of heterogeneity. To quantify this heterogeneity we calculated the proportion of the total variance due to between-study variance (Ri statistic) [36]. Furthermore, we explored the origin of heterogeneity by restricting the analysis to subgroups of studies defined by study characteristics such as study design, type of exposure (active or passive smoking), and age of the participants (children/adolescents or adults).

To check whether the pooled estimates were significantly different between subgroups we carried out a meta-regression with the global effect as dependent variable and the subgroup variable as moderator.

We assessed publication bias, first visually, using funnel plots and then, more formally, using the test proposed by Egger and colleagues [37]. We also used the trim-and-fill method to correct for potential publication bias. All analyses were performed with the software HEpiMA version 2.1.3 [38] and STATA version 12 with its macros metabias, metareg, and metatrim.

The secondary analyses (children and adolescents/adults, ISAAC/other, cohort and case-control studies combined/cross-sectional studies, high quality/low quality) were planned a priori.

Results

We identified 196 studies, published in 139 different articles and carried out in 51 countries, on active or passive smoking and allergic diseases that met our inclusion criteria (Figure 1). The data from one study were obtained from the authors [39]. We found 97 studies on allergic rhinitis [19,21,24,39–118], 91 on allergic dermatitis [19,20,22,24–26,44–46,48,53,60–65,67,73,75,76,78,83–87,91,93,96,97,99,101–103,105–107,110,111,116–165], and eight on food allergies [14,23,26,73,126,136,166–168].

A large majority of the articles retrieved initially were excluded either because they did not provide any effect measure or the outcome was allergy at large. More specifically, of the studies that could have been relevant to our meta-analysis but were finally excluded, eight were discarded because they were an early version of cohort studies updated in subsequent publications [169–176]. Other studies published their results several times [175–181] in which case we chose to include the most complete report. Some studies were excluded because the outcome was not allergic rhinitis, dermatitis, or food allergy but rather SPT or IgE concentrations

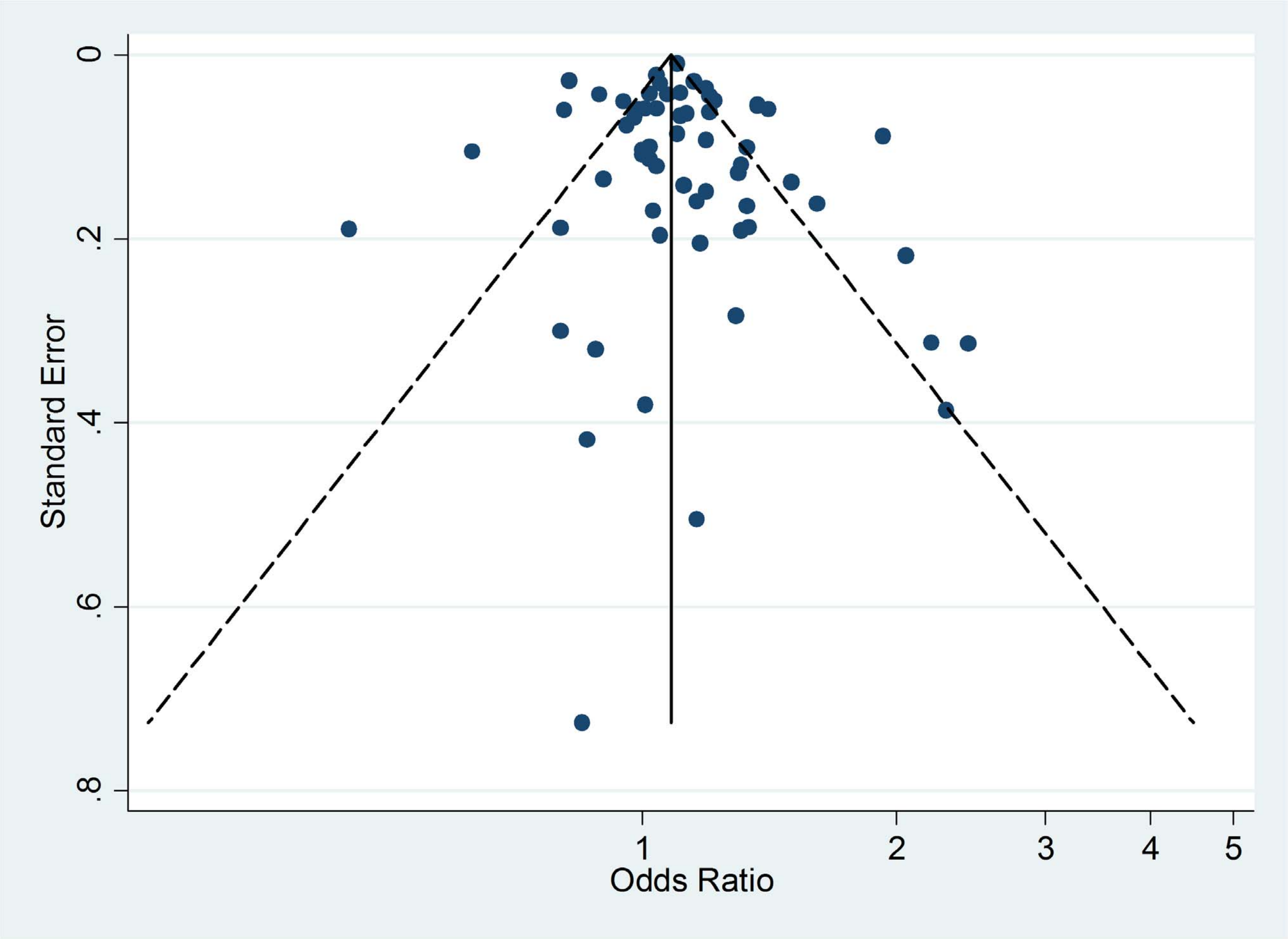

Figure 5. Funnel plots of relative risk versus standard error of relative risk: allergic rhinitis, passive smoking.
doi:10.1371/journal.pmed.1001611.g005

[182–192]. We also excluded nine studies that used either unspecific outcomes such as nasal symptoms [193,194], or a mixture of allergic diseases as a single outcome [195–201]. Eight studies [138,202–208] were excluded as they did not present any effect measure. Finally, one ecologic study was not considered further [209].

Globally, heterogeneity was substantial overall and similarly high after stratification by design, quality features (including adjustment for confounders), and study population. Given the substantial heterogeneity, we focused on the random effects analyses; however, the fixed effects analyses are presented for comparison and only discussed where they differ.

Allergic Rhinitis

Thirty-four studies on active smoking and 63 studies on passive smoking were available (Figures 2 and 3; Tables 1 and 2). The overwhelming majority of the studies assessed diagnosis through questionnaire and only seven studies used SPT or IgE measurements for the case definition [39,42,46,52,57,101,113]. The study by Wright and colleagues [42] measured SPT reactivity but used a definition of physician diagnosed allergic rhinitis that included both SPT-positive and SPT-negative children. More than half of the studies used ISAAC criteria for the definition of allergic rhinitis. Finally, 11 studies assessed maternal smoking during pregnancy [44,45,47–49,60,70,81,93,99,114].

Table 3 shows the results for associations between smoking and allergic rhinitis.

Active Smoking

Using random effects analysis, there was no significant association between active smoking and the risk of allergic rhinitis when all studies are considered (RR = 1.02; 95% CI 0.92–1.15). Using fixed effect analysis for all studies, there was a significant association between active smoking and risk of rhinitis (RR = 1.06, 95% CI 1.03–1.08); however, this may be explained by the considerable amount of heterogeneity due to differences in designs, case, and exposure definitions and adjustment for confounders. It is remarkable that, under the fixed effects model, the result of the cross-sectional subgroup (RR = 1.09; 95% CI 1.06–1.12) is statistically significant and opposed to the result of the cohort studies subgroup (RR = 0.87; 95% CI 0.82–0.93).

When restricting the analysis to the ten studies carried out on children and adolescents, active smoking was associated with an increased pooled relative risk of 1.40 (95% CI 1.24–1.59). In further sub-group analyses, the association was significant in the studies that used the standardized ISAAC protocol (RR = 1.50, 95% CI 1.35–1.66), but not those that used their own protocol (RR = 0.96, 95% CI 0.88–1.08). A reverse association between active smoking and allergic rhinitis was observed in adults only (RR = 0.90, 95% CI 0.82–0.99)

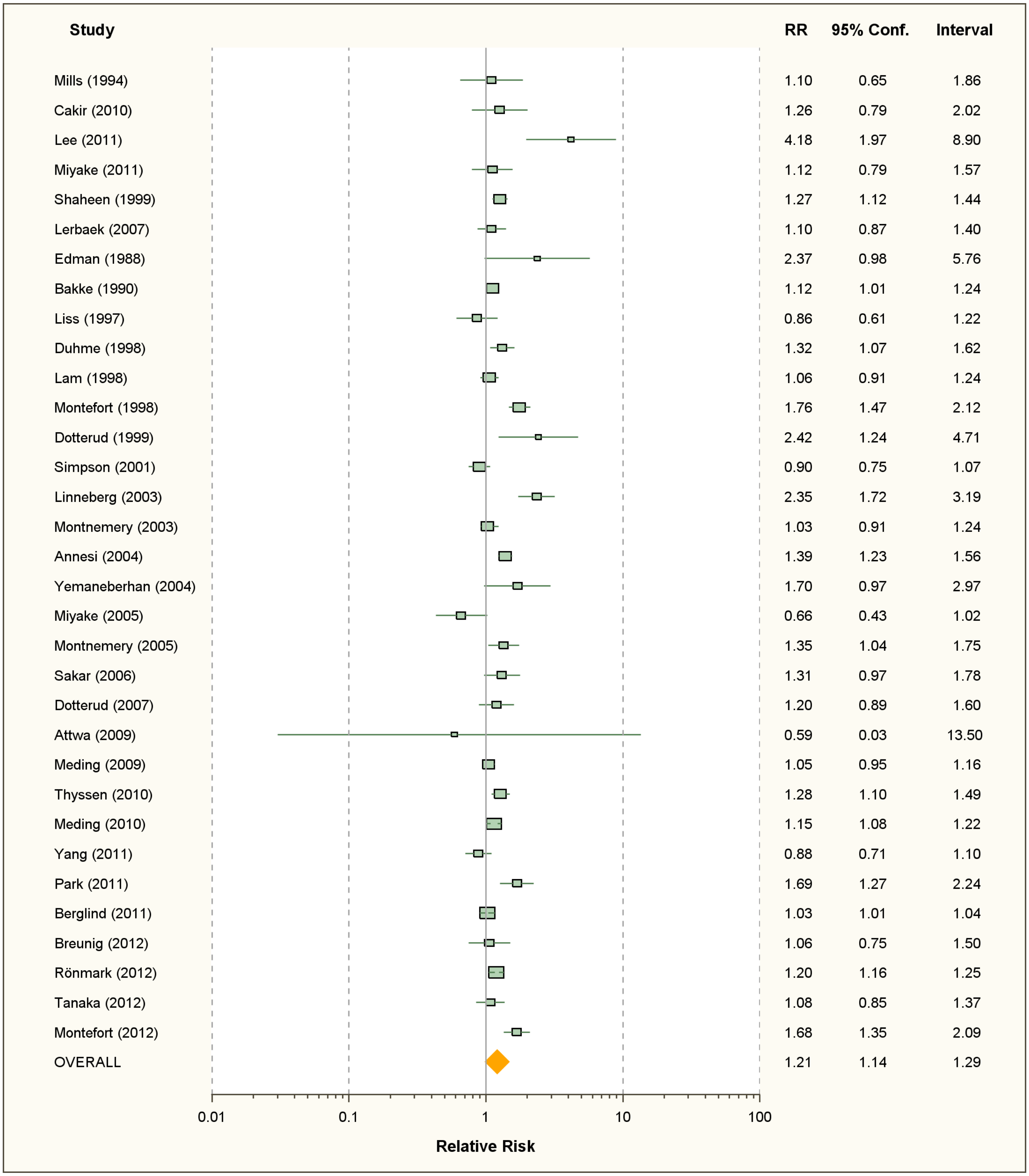

Figure 6. Study-specific and random effects pooled relative risks of active smoking and allergic dermatitis.
doi:10.1371/journal.pmed.1001611.g006

Passive Smoking

Using random effects analysis, there was a significant association passive smoking and allergic rhinitis (RR = 1.10, 95% CI 1.06–1.15). Similar findings were observed in subgroup analyses by adjustment for confounding variables (RR = 1.07; 95% CI 1.03–1.12 for full adjustment, RR = 1.15; 95% CI 1.04–1.27 for incomplete adjustment), quality scores (RR = 1.10; 95% CI 1.04–1.15 for high quality, RR = 1.10; 95% CI 1.02–1.19 for low quality), and for cross-sectional studies (RR = 1.09; 95% CI 1.05–1.14); however, there was no significant association between passive smoking and allergic rhinitis when restricting the analysis to cohort studies (RR = 1.14; 95% CI 0.96–1.34) or case-control studies (RR = 1.14; 95% CI 0.46–2.82).

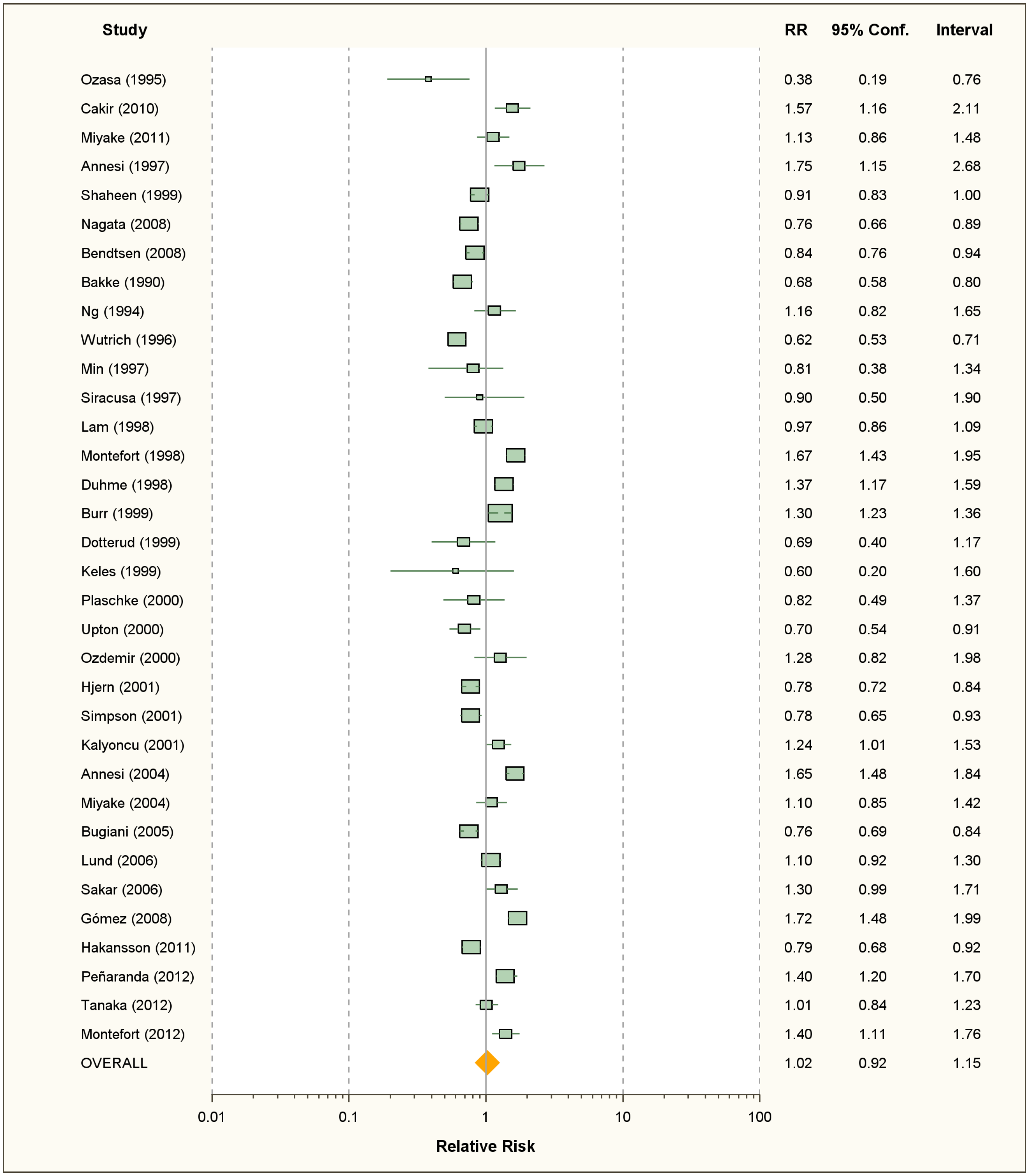

Figure 7. Study-specific and random effects pooled relative risks of passive smoking and allergic dermatitis.
doi:10.1371/journal.pmed.1001611.g007

In subgroup analyses based on age group, a significant association between passive smoking and allergic rhinitis was observed in adults only (RR = 1.17; 95% CI 1.03–1.32) and in children and adolescents (RR = 1.09; 95% CI 1.04–1.14). For maternal pregnancy smoking, there was no evidence for a a significant increase in the risk of allergic rhinitis in the offspring (RR = 1.07; 95% CI 0.92–1.28).

Publication Bias

The funnel plot of active smoking seems to be slightly skewed to the left, which indicates a potential lack of studies that favor a positive association of the disease with smoking (Figure 4). However, the Egger's test of asymmetry yielded a nonsignificant p-value of 0.27 and no hypothetical study was suggested as missing in the trim-and-fill procedure. The funnel plot for passive

Table 4. Relative risks and 95% confidence intervals of dermatitis by smoking exposure in case-control and cohort studies.

Source	Country	Population	Follow-up (y)	Complete Follow-up (%)	Active Smoking	Passive Smoking	Cases/Controls or Cohort Size	Variables of Adjustment, Matching, Restriction
**Case-control studies**								
Mills 1994 [119]	UK	Adults	—	—	1.1 (0.65–1.86)	—	127/127	Not specified
Yang 2000 [120]	Taiwan	School children	—	—	—	0.75 (0.46–1.25)	144/144	Age, sex, parental education, breast feeding, parental eczema
Purvis 2005 [121]	New Zealand	Children	—	—	—	—	87/463	Age
Haileamlak 2005 [122]	Ethiopia	Children	—	—	—	1.07 (0.75–1.54)	306/426	Age
Sebok 2006 [123]	Hungary	Children	—	—	—	1.15 (0.93–1.42)	461/343	Age, sex, residence
Wang 2010 [25]	Taiwan	Children	—	—	—–	1.02 (0.43–2.43)	34/106	Age
Cakir 2010 [19]	Turkey	Adolescents	—	—	1.26 (0.79–2.02)	—	436/366	Age, sex, family atopy, pets, income, occupation
Lee 2011 [20]	Taiwan	Adults	—	—	4.18 (1.97–8.90)	2.22 (1.01–4.84)	83/142	Age, sex
Miyake 2011 [124]	Japan	Adult women	—	—	1.12 (0.79–1.57)	—	188/1,082	Age, sex, residence, siblings, education
**Cohort studies**								
Burr 1989 [125]	UK	Infants	1	93.1	—	2.03 (1.28–3.22)	184/468	Age
Zeiger 1995 [126]	USA	High risk children	7	57	—	7.9 (1.0–61.0)	9/165	Age, sex, maternal ethnicity, parental asthma, food allergy
Olesen 1997 [127]	Denmark	Children	9.5	93	—	—	184/985	Age, sex, mother's age at birth, parity, birth weight, family atopy
Lewis 1998 [30]	UK	Children	16	55	—	1.05 (0.94–1.18)	1,213/6,352	Age, sex, social class, birth weight, gestational age, breast feeding, maternal age, parity
Tariq 1998 [26]	UK	Children	4	83.6	—	0.58 (0.36–0.94)	145/1,218	Age
Shaheen 1999 [45]	UK	Adults	26	51.1	1.27(1.12–1.44)	—	?/6,420	Age, sex, birth weight, social class, siblings, qualification, height, body mass index
Bergmann 2000 [46]	Germany	Infants	6	75	—	1.21 (0.83–1.76)	206/825	Age, sex, parental atopy, socioeconomic status, breast feeding, aeroallergen and food sensitization, study centre
McKeever 2001 [24]	UK	Children	11	95	—	0.89 (0.87–0.92)	8,839/29,238	Age, sex, family atopy, siblings
Bergmann 2002 [128]	Germany	Infants	7	71.5	—	—	?/937	Age, sex, breastfeeding duration, familial atopy, social status, allergic rhinoconjunctivitis, asthma, upper respiratory tract infections, mother's age, parity, IgE.
Kerkhof 2003 [129]	Netherlands	Infants	1	?	—	1.17 (0.72–1.89)	76/304	Sex, age, birth weight, gestation age, mother's age, breastfeeding, siblings, day-care attendance, pets, region, parental education

Table 4. Cont.

Source	Country	Population	Follow-up (y)	Complete Follow-up (%)	Active Smoking	Passive Smoking	Cases/Controls or Cohort Size	Variables of Adjustment, Matching, Restriction
Ludvigsson 2005 [21]	Sweden	Infants	1	66.5	—	0.83 (0.71–0.96)	2,038/8,784	Age, sex, pets, preterm birth, maternal education, parity, parental atopy
Magnusson 2005 [48]	Denmark	Children	18	74	—	1.0 (0.8–1.1)	1,248/7,844	Sex, social class, occupation, maternal age at pregnancy, coffee, parity, breastfeeding
Linneberg 2006 [130]	Denmark	Infants	1.5	67	—	—	3,327/34,793	Age, sex, breast feeding, parental atopy, season of birth, gestation age, head circumference, birth weight, residence, maternal occupation, household income, siblings, day care attendance, pets
Lerbaek 2007 [131]	Denmark	Twin adults	9	82	1.10 (0.87–1.40)	—	244/3,393	Not specified
Noakes 2007 [132]	Australia	Infants	1	67	—	0.80 (0.28–2.24)[a]	41/82	Age
Sariachvili 2007 [133]	Belgium	Infants	1	87	—	1.8 (1.0–3.1)	227/975	Age, sex, parental atopy, pregnancy duration, maternal educational and age, pets, antibiotics use, parity, day care attendance.
Tanaka 2008 [134]	Japan	Children	2	76	—	1.10 (0.86–1.41)	142/763	Age, sex, birth weight, family income, parental atopy, pets, older siblings, maternal age
Böhme 2010 [135]	Sweden	Children	4	61.2	—	1.68 (1.22–2.30)	529/2,505	Age, sex, parental atopy, breastfeeding, pets, parental education.
Jedrychowski 2011 [136]	Poland	Infants	1	100	—	1.46 (0.84–2.55)	183/469	Age

[a]Relative risk for pre- and post- natal smoking of mother.

doi:10.1371/journal.pmed.1001611.t004

Table 5. Relative risks and 95% confidence intervals of allergic dermatitis by smoking exposure in cross-sectional studies.

Source	Country	Population	Active Smoking	Passive Smoking	Study Size	Variables of Adjustment, Matching, or Restriction
Edman 1988 [137]	Sweden	Adults	2.37 (0.98–5.76)	—	425	Not specified
Bakke 1990 [53]	Norway	Adolescents and adults	1.12 (1.01–1.24)	—	4,270	Age, sex, occupational exposure, residence
Volkmer 1995 [138]	Australia	Preschool children	—	0.80 (0.71–0.91)	14,124	Natural gas for cooking, heating and cooling sources
Austin 1997 [60]	UK	Children	—	0.88 (0.74–1.05)	1,537	Age
Liss 1997 [139]	Canada	Adults	0.86 (0.61–1.22)	—	1,326	Not specified
Schäfer 1997 [140]	Germany	Preschool children	—	—	678	Age
Duhme 1998 [65]	Germany	Schoolchildren	1.32 (1.07–1.62)	0.97 (0.85–1.10)	13,123	Age, sex
Lam 1998 [62]	Hong Kong	Schoolchildren	1.06 (0.91–1.24)	0.91 (0.80–1.03)	6,304	Age, sex, residence, housing
Montefort 1998 [64]	Malta	Schoolchildren	1.76 (1.47–2.12)	—	4,184	Age, sex, road, pets, parental atopy, blankets
Farooqi 1998 [61]	UK	Children	—	0.97 (0.75–1.26)*	1,934	Not specified
Dotterud 1999 [67]	Russia	Adults	2.42 (1.24–4.71)	—	3,368	Not specified
Dotterud 2001 [76]	Russia	Schoolchildren	—	0.93 (0.78–1.11)	1,684	Age, sex, carpets, dampness, pets, heating type
Hjern 2001 [73]	Sweden	Children	—	0.88 (0.75–1.03)	4,472	Age, sex, siblings, parental education, residence, single parent household, country of birth of parent, location
Lee 2001 [78]	Korea	Schoolchildren	—	1.09 (0.99–1.20)	38,955	Age, sex, region, BMI, carpets, pets, location
Simpson 2001 [75]	UK	Adults	0.9 (0.75–1.07)	—	5,687	Not specified
Linneberg 2003 [141]	Denmark	Adolescents and adults	2.35 (1.72–3.19)	—	1,112	Age, sex, ear piercing
Montnemery 2003 [142]	Sweden	Adults	1.03 (0.91–1.24)	—	8,469	Not specified
Kramer 2004 [84]	Germany	School beginners	—	1.97 (1.23–3.16)	1,220	Age, sex, atopy, nationality
Demir 2004 [85]	Turkey	Schoolchildren	—	1.30 (0.46–3.83)	621	Age
Annesi-Maesano 2004 [87]	France	Adolescents	1.39 (1.23–1.56)	0.9 (0.9–1.3)	14,578	Age, sex
Miyake 2004 [86]	Japan	Schoolchildren	—	1.04 (0.89–1.22)	5,539	Age, sex, grade, older siblings, maternal age at child birth, pets, parental allergic diseases.
Yemaneberhan 2004 [143]	Ethiopia	Children and adults	1.70 (0.97–2.97)	2.13 (1.31–3.46)	12,876	Age, sex, socioeconomic status, residence, kerosene use
Lee 2004 [83]	Hong Kong	Schoolchildren	—	—	4,448	Age, sex, birth weight, siblings, respiratory tract infections, parental atopy, pets, study period
Heudorf 2005 [144]	Germany	Children	—	2.34 (1.04–5.28)	287	Age
Miyake 2005 [91]	Japan	Pregnant women	0.66 (0.43–1.02)	1.08 (0.81–1.44)	1,002	Age, sex, familial atopy, pets, gestation, parity, family income, education, mite antigen level
Montnemery 2005 [145]	Sweden	Adults	1.35 (1.04–1.75)	—	6,109	Not specified
Obihara 2005 [93]	South Africa	Children	—	—	861	Age, sex, maternal atopy, breast feeding, siblings, household income, tuberculin test
Kurosaka 2006 [96]	Japan	Schoolchildren	—	0.99 (0.93–1.05)	35,242	Age
Sakar 2006 [97]	Turkey	Adults	1.31 (0.97–1.78)	—	1,336	Not specified
Dotterud 2007 [146]	Norway	Adults	1.20 (0.89–1.60)	—	1,236	Age, sex, atopic dermatitis, rhinitis and asthma
Horak 2007 [99]	Austria	Preschool children	—	1.06 (0.76–1.48)	1,737	Age, sex, familial atopy, education, family size, pets, breastfeeding, nutrition

Table 5. Cont.

Source	Country	Population	Active Smoking	Passive Smoking	Study Size	Variables of Adjustment, Matching, or Restriction
Tanaka 2007 [101]	Japan	Children	—	1.08 (1.02–1.14)	23,044	Age, sex, location, familial atopy, siblings, education level
Zuraimi 2008 [102]	Singapore	Preschool children	—	1.02 (0.95–1.09)	4,759	Age, familial atopy, race, socioeconomic status, housing type, breastfeeding, food allergy, respiratory infections, dampness
Al-Sahab 2008 [147]	Lebanon	Adolescents	—	1.46 (1.11–1.94)	2,893	Age, sex, exercise, traffic, asthma, rhinitis
Ergin 2008 [148]	Turkey	Schoolchildren	—	1.30 (0.89–1.91)	1,644	Age
Foliaki 2008 [103]	Pacific countries	Children	—	1.20 (1.11–1.30)	20,876	Age, sex and country
Suárez-Varela 2008 [149]	Spain	Schoolchildren	—	1.03 (0.99–1.06)	59,040	Age, sex, asthma, rhinitis, siblings, mother's education
Attwa 2009 [150]	Egypt	Adult men	3.59 (1.0–13.5)	—	163	Sex
Meding 2009 [151]	Sweden	Adults	1.05 (0.95–1.16)	—	13,452	Age, sex, history of atopy
Brescianini 2009 [106]	Italy	Schoolchildren	—	1.22 (0.77–1.95)	481	Age, sex, family atopy, BMI, pets, physical activity, diet, location
Musharrafieh 2009 [107]	Lebanon	Adolescents	—	1.1 (0.9–1.4)	3,115	Age, sex, nationality, regions, school type, traffic, asthma, rhinitis
Kabir 2009 [105]	Ireland	Children	—	1.24 (0.90–1.70)	2,809	Age, sex
Lipinska 2009 [152]	Poland	Children	—	3.40 (1.19–11.86)	283	Not specified
Xepapadaki 2009 [153]	Greece	Preschool children	—	0.98 (0.79–1.22)	2,374	Age, sex
Wang 2010 [110]	Canada	Schoolchildren	—	1.05 (0.79–1.41)	8,334	Age, sex, BMI, location, birthplace, ethnicity, maternal education, siblings, pets, acetaminophen, physical activity
Röhrl 2010 [154]	Sweden	Adolescents	—	0.85 (0.59–1.23)	6,095	Age, sex, flexural eczema and nickel allergy
Thyssen 2010 [155]	Denmark	Adults	1.28 (1.10–1.49)	—	3,471	Age, sex, alcohol consumption, educational level
Meding 2010 [156]	Sweden	Adults	1.15 (1.08–1.22)	—	25,428	Age, sex, history of atopy
Yang 2011 [157]	USA	Adults	0.88 (0.71–1.10)	1.21 (0.85–1.74)	2,974	Not specified
Vlaski 2011 [111]	Macedonia	Adolescents	—	0.93 (0.80–1.09)	3,026	Age, sex, diet, source of heating, pets, maternal education, siblings
Civelek 2011 [158]	Turkey	Schoolchildren	—	1.35 (1.17–1.56)	6,755	Age
Dei-Cas 2011 [159]	Argentina	Children	—	1.45 (1.02–2.08)	722	Age
Apfelbacher 2011 [160]	Germany	Children and adolescents	—	0.90 (0.77–1.04)	17,270	Age, sex, socioeconomic status, migrant status, siblings, breastfeeding, mother's alcohol consumption, pets, infection, parental atopy.
Park 2011 [161]	Korea	Adults	1.69 (1.27–2.24)	—	1,990	Age, sex, BMI, education, income, alcohol, fish consumption.
Berglind 2011 [162]	Sweden	Adults	1.03 (1.01–1.04)	—	27,793	Neck and shoulder pain, depression, well-being, job strain, low back pain, physical activity at work
Breunig 2012 [163]	Brazil	Male adolescents	1.06 (0.75–1.50)	—	2,201	Age, sex, white race, socioeconomic level, triceps skin fold, acne

Table 5. Cont.

Source	Country	Population	Active Smoking	Passive Smoking	Study Size	Variables of Adjustment, Matching, or Restriction
Yi 2012 [164]	Korea	Children	—	1.30 (1.23–1.38)	6,372	Age, sex, residence, income, parental education, atopy, IgE level, rhinitis
Rönmark 2012 [165]	Sweden	Adults	1.20 (1.16–1.25)	—	18,087	Age, sex, family history of atopy, exposure to gas, dust or fumes at work
Tanaka 2012 [116]	Japan	Pregnant women	1.08 (0.85–1.37)	1.42 (0.99–2.05)	1,743	Age, sex, region of residence; parental atopy, income, education
Montefort 2012 [117]	Malta	Children	1.68 (1.35–2.09)	1.24 (1.13–1.37)	7,955	Age
Mitchell 2012 [118]	Worldwide	Children	—	1.11 (1.09–1.14)	573,061	Age, sex, language, region, gross national income

doi:10.1371/journal.pmed.1001611.t005

smoking (Figure 5) and the corresponding results of the Egger's test did not show any evidence of publication bias ($p = 0.53$), but two new studies were imputed in the trim-and-fill procedure yielding a modified pooled relative risk of 1.10 (95% CI 1.05–1.14).

Allergic Dermatitis

We retrieved 33 studies on active smoking and 58 studies on passive smoking (Figures 6 and 7; Tables 4 and 5). About one-third of the studies used ISAAC criteria for case definition. Nineteen

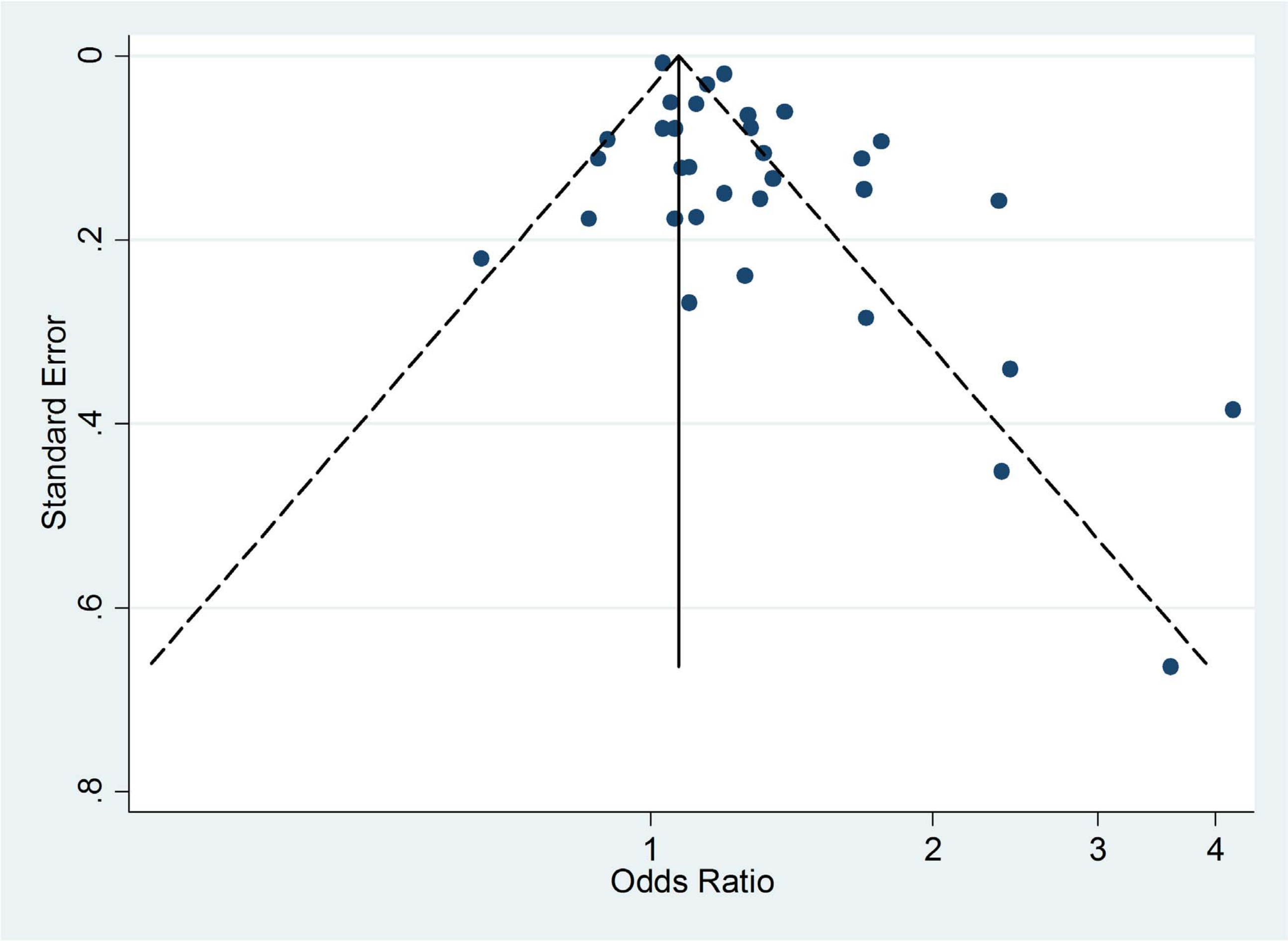

Figure 8. Funnel plots of relative risk versus standard error of relative risk: allergic dermatitis, active smoking.
doi:10.1371/journal.pmed.1001611.g008

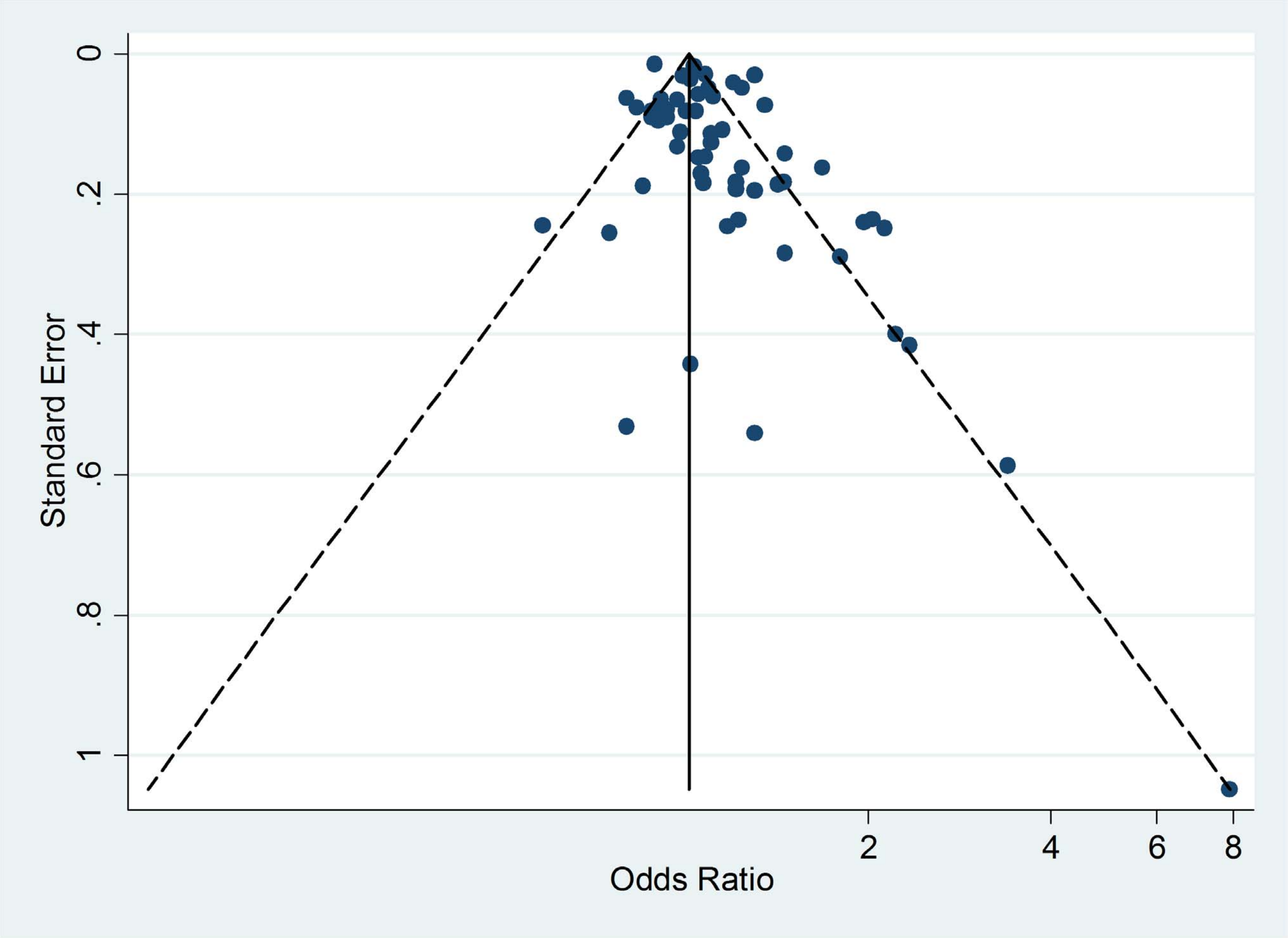

Figure 9. Funnel plot of relative risk versus standard error of relative risk: allergic dermatitis, passive smoking.
doi:10.1371/journal.pmed.1001611.g009

studies assessed maternal smoking during pregnancy [44,45,48, 83,93,99,121,127,128,130,133–136,140,153,158,160,164].

Active Smoking

Using random effects analysis, active smoking was significantly associated with an increased risk of allergic dermatitis overall (RR = 1.21; 95% CI 1.14–1.29) and in both adults (RR = 1.14; 95% CI 1.07–1.22) and in children and adolescents (RR = 1.36; 95% CI 1.17–1.46)

In sub-group analyses, the association between active smoking and allergic dermatitis was similar based on age, adjustment for confounding, quality scores, and for cohort studies and cross-sectional studies, although there was no significant association between active smoking and allergic dermatitis observed in the four case-control studies (RR = 1.47; 95% CI 0.92–2.32).

Passive Smoking

Using random effects analysis, passive smoking was associated with an increased risk of allergic dermatitis in the general population (RR = 1.07; 95% CI 1.03–1.12).

In sub-group analyses, the association between passive smoking and allergic dermatitis was significant when restricted to cross-sectional studies (RR = 1.07; 95% CI 1.02–1.12), but not for cohort (RR = 1.09; 95% CI 0.96–1.23) or case-control studies (RR = 1.10; 95% CI 0.88–1.38). A significant association between passive smoking and allergic dermatitis was observed for those studies with adjustment for confounding variables (RR = 1.08; 95% CI 1.03–1.13) and higher quality scores (RR = 1.11; 95% CI 1.05–1.18), but not those without adjustment (RR = 1.06; 95% CI 0.98–1.14) or low quality scores (RR = 1.03; 95% CI 0.96–1.11).

A significant association was observed in those studies including adults only (RR = 1.26; 95% CI 1.02–1.55) and in those including children and adolescents only (RR = 1.06; 95% CI 1.01–1.11). No significant association was observed between maternal smoking and allergic dermatitis (RR = 1.07; 95% CI 0.96–1.19).

Publication Bias

The Egger's test for asymmetry of the funnel plot of active smoking (Figure 8) yielded a *p*-value of 0.28 and no study was added in the trim-and-fill procedure. No asymmetry was detected for passive smoking (Figure 9) through the Egger's test ($p = 0.33$) but the trim-and-fill procedure suggested that ten potential studies were missing. The modified random effects pooled relative risk was 1.04 (95% CI 1.00–1.08).

Food Allergies

We retrieved only one study for active smoking and six studies for passive smoking, while three studies assessed maternal smoking

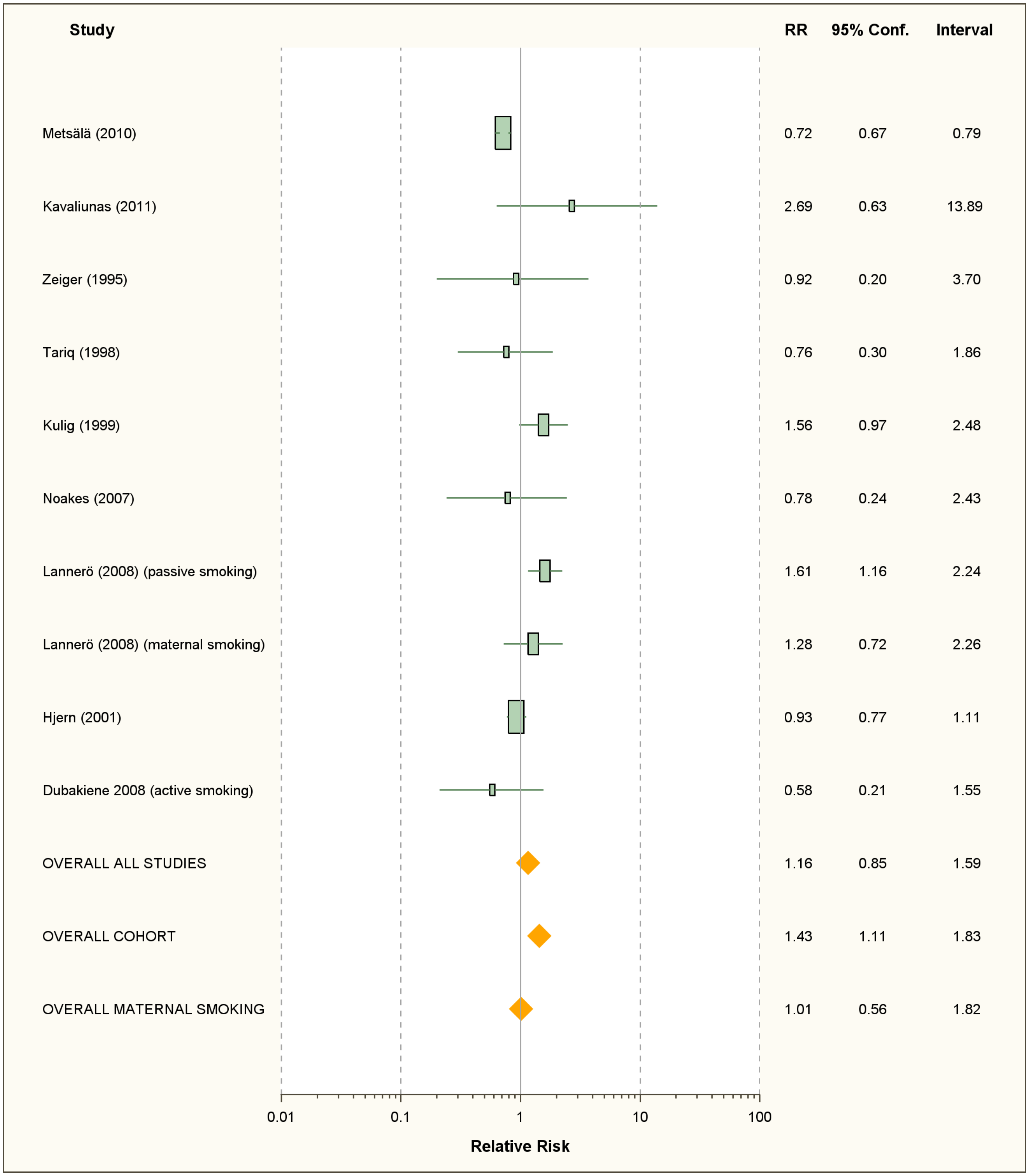

Figure 10. Study-specific and random effects pooled relative risks of passive smoking and food allergies.
doi:10.1371/journal.pmed.1001611.g010

during pregnancy (Figure 10; Table 7). All were carried out in children or infants populations.

Active Smoking

The only available study on active smoking and food allergies did not show any significant association (RR = 0.58; 95% CI 0.21–1.55).

Passive Smoking

Using random effect analysis, including the six studies investigating exposure to secondhand smoke, showed that passive smoking was associated with a nonsignificant increase of the risk of food allergy (RR = 1.16; 95% CI 0.85–1.59). When the only cross-sectional study was excluded and the analysis was based on five

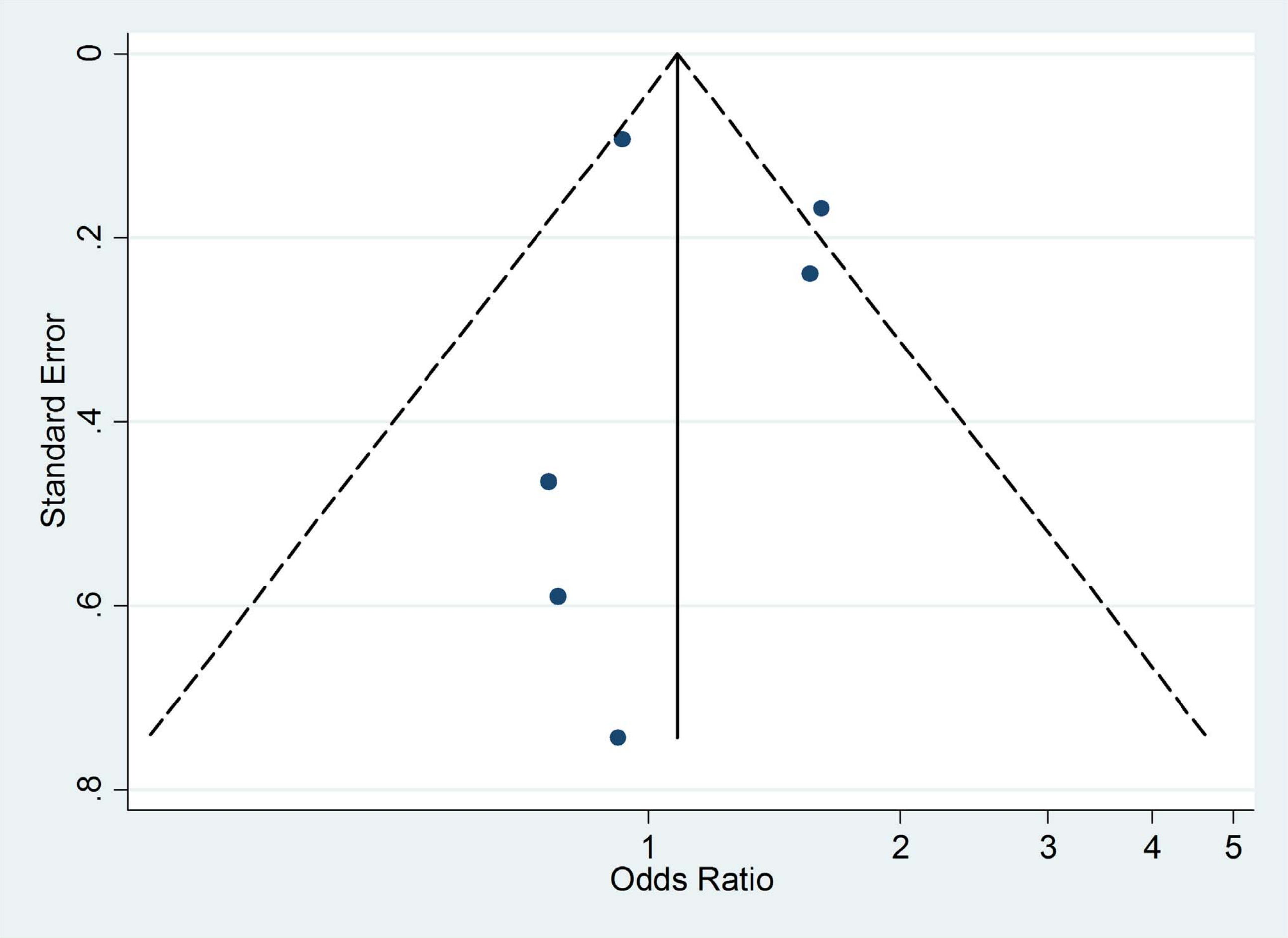

Figure 11. Funnel plot of relative risk versus standard error of relative risk: food allergy, passive smoking.
doi:10.1371/journal.pmed.1001611.g011

cohort studies, passive smoking was significantly associated with an increased risk of food allergy (RR = 1.43; 95% CI 1.12–1.83) (Table 8). As with allergic rhinitis and allergic dermatitis, we could not detect any association with maternal smoking during pregnancy with food allergies (RR = 1.01; 95% CI 0.56–1.82) (Table 8).

Publication Bias

The funnel plot (Figure 11), although not a valuable way to assess publication bias in this case due to the small sample size, did not provide evidence of asymmetry ($p = 0.09$).

Meta-regression

The meta-regression with the pooled log relative risk as a dependent variable and the population variable as a moderator, introduced in the model as a dichotomous variable (adults/pediatric population), yielded the following results for the children and adolescents when compared to the adults: allergic rhinitis and active smoking: RR = 1.55, 95% CI 1.30–1.84; allergic rhinitis and passive smoking: RR = 0.93, 95% CI 0.81–1.06; allergic dermatitis and active smoking: RR = 1.18, 95% CI 1.01–1.39; and allergic dermatitis and passive smoking: RR = 0.83, 95% CI 0.65–1.06. These results suggest that the associations between allergic rhinitis and allergic dermatitis with active smoking are significantly greater among children and adolescents than among adults. Although these meta-regression RRs were not statistically significant at a 95% level for passive smoking, in Tables 3 and 6 we present the results of children and adolescent populations as a subgroup both for active and passive smoking.

Sub-group Analyses in Children and Adolescents

We calculated the random effects pooled relative risks for children cohort studies, then for children cohort studies and case-control studies combined. For cohort studies, passive smoking was not significantly associated with allergic rhinitis (RR = 1.14; 95% CI 0.96–1.34, nine studies), or allergic dermatitis (RR = 1.09; 95% CI 0.96–1.23, 14 studies), but was significantly associated with an increased risk of food allergy (RR = 1.43; 95% CI 1.11–0.83, five studies). For cohort and case-control studies combined, passive smoking was significantly associated with an increased risk for allergic rhinitis: RR = 1.17 (95% CI 1.00–1.38, ten studies), but not for allergic dermatitis: RR = 1.07 (95% CI 0.96–1.19, 18 studies).

Sensitivity Analysis

To further evaluate the possibility that the results obtained for children/adolescents were due to publication bias, we assumed that cross-sectional studies are the kind of studies that are most probably rejected by journals in case of null results and recalculated our pooled estimates under the following extreme assumptions: (1) published cross-sectional studies are only half of

Table 6. Pooled relative risks and 95% confidence intervals of allergic dermatitis and smoking.

Study Type	Number of Studies	RR (95% CI) Fixed Effects	RR (95% CI) Random Effects	Ri[a] (95% CI)	Q test (*p*-Value)
**Active smoking**					
All studies	33	1.05 (1.04–1.06)	1.21 (1.14–1.29)	0.96 (0.91–1.00)	0.00001
Cohort studies	2	1.23 (1.10–1.38)	1.23 (1.09–1.38)	0.10 (0.00–1.00)	0.3003
Case-control studies	4	1.30 (1.03–1.64)	1.47 (0.92–2.32)	0.73 (0.27–1.00)	0.0160
Cross-sectional studies	27	1.05 (1.04–1.06)	1.21 (1.13–1.30)	0.97 (0.92–1.00)	0.00001
Cohort+case-control studies	6	1.24 (1.13–1.38)	1.27 (1.04–1.56)	0.67 (0.11–1.00)	0.0412
Full adjustment	17	1.05 (1.04–1.06)	1.21 (1.12–1.31)	0.98 (0.93–1.00)	0.00001
Incomplete adjustment	16	1.21 (1.14–1.29)	1.25 (1.09–1.45)	0.77 (0.56–0.98)	0.00001
Adults only	23	1.04 (1.03–1.05)	1.14 (1.07–1.22)	0.96 (0.89–1.00)	0.00001
Children/adolescents only	7	1.36 (1.27–1.46)	1.36 (1.17–1.46)	0.76 (0.44–1.00)	0.0008
Quality score ≥3	17	1.18 (1.13–1.23)	1.22 (1.11–1.34)	0.78 (0.55–1.00)	0.00001
Quality score <3	16	1.04 (1.03–1.05)	1.22 (1.11–1.34)	0.98 (0.94–1.00)	0.00001
**Passive Smoking**					
All studies	58	1.04 (1.03–1.06)	1.07 (1.03–1.12)	0.84 (0.71–0.98)	0.00001
Cohort studies	14	0.92 (0.90–0.95)	1.09 (0.96–1.23)	0.89 (0.72–1.00)	0.00001
Case-control studies	5	1.11 (0.94–1.31)	1.10 (0.88–1.38)	0.34 (0.00–1.00)	0.2411
Cross-sectional studies	39	1.08 (1.06–1.09)	1.07 (1.02–1.12)	0.81 (0.63–0.99)	0.00001
Cohort+case-control studies	19	0.93 (0.90–0.95)	1.09 (0.98–1.21)	0.86 (0.66–1.00)	0.00001
Full adjustment	32	1.08 (1.07–1.10)	1.08 (1.03–1.13)	0.81 (0.60–1.00)	0.00001
Incomplete adjustment	26	0.96 (0.94–0.98)	1.06 (0.98–1.14)	0.84 (0.65–1.00)	0.00001
Adults only	4	1.24 (1.03–1.50)	1.26 (1.02–1.55)	0.17 (0.00–1.00)	0.31
Children/adolescents only	53	1.04 (1.03–1.05)	1.06 (1.01–1.11)	0.85 (0.72–0.98)	0.00001
Children ISAAC method	22	1.07 (1.06–1.09)	1.09 (1.04–1.14)	0.73 (0.41–1.00)	0.00001
Children non-ISAAC method	29	0.99 (0.97–1.01)	1.05 (0.97–1.15)	0.89 (0.77–1.00)	0.00001
Maternal pregnancy smoking	19	0.99 (0.95–1.03)	1.07 (0.96–1.19)	0.80 (0.62–0.98)	0.00001
Quality score ≥3	28	1.04 (1.02–1.05)	1.11 (1.05–1.18)	0.88 (0.74–1.00)	0.00001
Quality score <3	30	1.06 (1.03–1.09)	1.03 (0.96–1.11)	0.80 (0.63–0.98)	0.00001

[a]Proportion of total variance due to between-study variance.
doi:10.1371/journal.pmed.1001611.t006

the studies of smoking and allergic rhinitis ever conducted among children, (2) all unpublished studies found an RR of 1, (3) the unpublished studies found the same prevalence of allergic diseases as the average of the published studies. Under these extreme assumptions, the random effects pooled estimates for active smoking still show a significant increase in risk: RR = 1.16 (95% CI 1.08–1.25) for allergic rhinitis and RR = 1.13 (95% CI 1.05–1.21) for allergic dermatitis.

Discussion

The results of our systematic review and meta-analysis suggest that active and passive smoking are associated with a modest increase in risk for some allergic diseases. In the overall population, active smoking was associated with a modest increase in the risk for allergic dermatitis but not allergic rhinitis, while passive smoking was associated with modest increases in the risks for both allergic dermatitis and allergic rhinitis. Among children and adolescents, we observed significant associations between both active and passive smoking and allergic rhinitis and allergic dermatitis, and passive smoking was associated with an increased risk for food allergy

In children and adolescents, while the observed increase in risk for allergic diseases associated with smoking was small, the findings are important given that to the prevalence of active and passive smoking in this population can be high. Worldwide, 14% of adolescents aged 13 to 15 are active smokers with some countries reaching a prevalence of 40%, and nearly 25% of the children who smoke have smoked their first cigarette before the age of 10 years [210]. Furthermore, in the US, more than one-third of children live with at least one adult smoker [211]. In other parts of the world, passive exposure to tobacco among children is even higher as nearly half of children were exposed to tobacco smoke at home [212]. On the basis of the figures above, in countries with high smoking prevalence we estimate that 14% of allergic rhinitis and 13% of allergic dermatitis are attributable to active smoking [213]. Eliminating active smoking in children and adolescents would then prevent one in every seven cases of allergic rhinitis and one in every eight cases of allergic dermatitis.

That age is an important effect modifier for the relation between tobacco exposure and risk of allergic diseases is biologically plausible. The US Surgeon General has suggested that the immaturity of the respiratory, nervous, and immune systems in children may make them vulnerable to health effects of smoking [214]. Furthermore, unlike adults, children have limited options for avoiding exposure to secondhand smoke and are unable to reduce the quantity of products inhaled [214].

Table 7. Study-specific and 95% confidence intervals of food allergies and smoking.

Author	Country	Population	Follow-up (y)	Complete Follow-up (%)	Active Smoking	Passive Smoking	Maternal Pregnancy Smoking	Cases/Controls or Cohort Size or Total Sample Size	Variables of Adjustment, Matching, or Restriction
**Case-control studies**									
Metsälä 2010 [23]	Finland	Infants	—	—	—	—	0.72 (0.67–0.79)	16,237/16,237	Age, multiple pregnancy, gestational age, ponderal index, socioeconomic status, previous deliveries
Kavaliunas 2011 [166]	Lithuania	Children	—	—	—	—	2.69 (0.63–13.89)	42/144	Age
**Cohort studies**									
Zeiger 1995 [126]	USA	Children	7	57	—	0.92 (0.20–3.70)	—	22/165	Age
Tariq 1998 [26]	UK	Children	4	83.6	—	0.76 (0.30–1.86)	—	34/1,280	Age
Kulig 1999 [167]	Germany	Children	3	?	—	1.56 (0.97–2.48)	—	?/328	Age, parental education, study center
Noakes 2007 [136]	Australia	Infants	1	67.2	—	0.78 (0.24–2.43)	—	25/82	Age
Lannerö 2008 [14]	Sweden	Children	4	62	—	1.61 (1.16–2.24)	1.28 (0.72–2.26)	331/2,529	Age, parental atopy, socioeconomic status
**Cross-sectional studies**									
Hjern 2001 [73]	Sweden	Children	—	—	—	0.93 (0.77–1.11)	—	4,472	Age, sex, siblings, parental education, residence, single parent household, country of birth of parents, location
Dubakiene 2008 [168]	Lithuania	Children	—	—	0.58 (0.21–1.55)	—	—	540	Not specified

doi:10.1371/journal.pmed.1001611.t007

Table 8. Pooled relative risks and 95% confidence intervals of food allergies and smoking.

Pooled Results	Passive Smoking All Studies	Passive Smoking Cohort Studies	Maternal Pregnancy Smoking
n studies	6	5	3
RR (95% CI), fixed effects	1.08 (0.93–1.24)	1.43 (1.12–1.83)	0.73 (0.67–0.80)
RR (95% CI), random effects	1.16 (0.85–1.59)	1.43 (1.11–1.83)	1.01 (0.56–1.82)
Ri[a] (95% CI)	0.68 (0.13–1.00)	0.01 (0.00–1.00)	0.96 (0.84–1.00)
Q Test (*p*-value)	0.0386	0.4026	0.0440

[a]Proportion of total variance due to between-study variance.
doi:10.1371/journal.pmed.1001611.t008

Our finding that maternal exposure is not associated with the risk of allergic diseases in the offspring confirms the results from a previous meta-analysis that focused on the risk of allergic sensitization measured through skin prick positivity or IgE concentrations [30]. It is possible that the lack of observed association is due to the existence of bias given that parents of children at high risk of allergy may selectively avoid smoking during pregnancy.

The findings from our meta-analysis are subject to several limitations. The majority of studies were cross-sectional, a design that does not allow for causal inference and can overestimate relative risks given its reliance on prevalence ratios. When restricted to cohort studies our analyses showed that many of the results were no longer significant, especially for the subgroup analysis in children and adolescents. There is then some evidence that the findings may be impacted by study design.

Residual confounding (confounding remaining after adjustment) may explain some of our findings. For some of our analyses, we were unable to detect meaningful differences in the results between studies that had incomplete adjustment for confounders and those with more complete adjustment for confounders and our findings were broadly similar when restricting the analyses to studies with higher quality scores. However, there are likely to be other factors, such as genetic factors that were not controlled for and may play a role in the relationship between smoking and allergic diseases. Although publication bias cannot be ruled out, its magnitude is likely to be low as shown by the robustness of our sensitivity analysis.

Several studies assessed allergic diseases through self-report only, which can lead to misclassification of allergic and non-allergic conditions. Similarly, the findings are limited by measurement error in the smoking exposure given that a majority of studies assessed exposure to smoking in a qualitative fashion and often on a yes/no basis instead of using a quantitative assessment. Misclassification and measurement error in SHS assessment may result from a respondent's lack of knowledge about current or past exposure, biased recall, whether intentional or unintentional, and the difficulty in characterizing an exposure in complex indoor environments [215]. A standard set of items to identify passive smoking in distinct settings is needed [216]. If misclassification exists, it is probable that the outcome misclassification is not differential in regard to smoking and, similarly, measurement error in smoking assessment is not differential in regard to diagnosis. In this case, the results would be biased towards the null value, which means that the association with smoking observed in our meta-analysis is underestimated.

In our subgroup analyses, we were unable to identify any factors that accounted for study heterogeneity. Given the high heterogeneity estimates, we focused our interpretation on the random effects estimates. The random effects model gives increased weights to the effect of small studies, which may introduce bias in the estimation. It is worth noting that for some of the analyses, the fixed effects and random effects estimates differ substantially; this may be due to differences in case or exposure definition and in adjustment for potential confounders.

Our subgroup analyses found stronger evidence for associations between smoking and allergic diseases in children and adolescents than adults. Furthermore, our meta-regression suggested that the association between active smoking and allergic disorders is larger in children and adolescents than in adults, which advocates for a transient effect through life. This finding is in accordance with the "atopic march" concept that suggests that the sequence of sensitization that starts in childhood may show a tendency to spontaneous remission later in life [217]. It is then plausible that sensitization to tobacco is mitigated by increasing age. Further research is needed to verify whether the association between smoking and risk of allergy in adults is similar for those who started smoking as an adult and those who started smoking during childhood or adolescents.

Future studies should minimize measurement error in the exposure and misclassification bias in the outcome. These studies should avoid cross-sectional designs, use extensive validated questionnaires in order to assess smoking in a quantitative fashion, and should be based on an optimal diagnosis of allergic diseases.

Supporting Information

Table S1 Quality scoring of allergic rhinitis, dermatitis, and food allergies studies.
(DOC)

Table S2 Pooled relative risks and 95% confidence intervals of criterion 1 of the quality scale, region of the world, and allergic rhinitis and dermatitis.
(DOC)

Table S3 Results of heterogeneity statistics Ri and I2 for subgroups of active and passive smoking.
(DOC)

Acknowledgments

We would like to thank Kotaro Ozasa for providing data of his study.

Author Contributions

Conceived and designed the experiments: JS CR AMM BT PK. Performed the experiments: JS CR AMM BT PK. Analyzed the data: JS BT.

Contributed reagents/materials/analysis tools: JS BT PK. Wrote the first draft of the manuscript: JS BT. Contributed to the writing of the manuscript: JS CR AMM BT. ICMJE criteria for authorship read and met: JS CR AMM PK BT. Agree with manuscript results and conclusions: JS CR AMM PK BT.

References

1. Ozdoganoglu T, Songu M (2012) The burden of allergic rhinitis and asthma. Ther Adv Respir Dis 6: 11–23.
2. Bauchau V, Durham SR (2004) Prevalence and rate of diagnosis of allergic rhinitis in Europe. Eur Respir J 24: 758–764.
3. Berger WE (2004) Allergic rhinitis in children: diagnosis and management strategies. Paediatr Drugs 6: 233–250.
4. Rona RJ, Keil T, Summers C, Gislason D, Zuidmeer L, et al. (2007) The prevalence of food allergy: a meta-analysis. J Allergy Clin Immunol 120: 638–646.
5. DaVeiga SP (2012) Epidemiology of atopic dermatitis: a review. Allergy Asthma Proc 33: 227–234
6. Meltzer EO, Blaiss MS, Derebery MJ, Mahr TA, Gordon BR, et al. (2009) Burden of allergic rhinitis: results from the Pediatric Allergies in America survey. J Allergy Clin Immunol 124: S43–S70
7. Nathan RA (2007) The burden of allergic rhinitis. Allergy Asthma Proc 28: 3–9.
8. Alanne S, Maskunitty A, Nermes M, Laitinen K, Pekurinen M (2012). Costs of allergic diseases from birth to two years in Finland. Public Health 126: 866–872
9. Spergel JM (2010) From atopic dermatitis to asthma: the atopic march. Ann Allergy Asthma Immunol 105: 99–106
10. Tan RA, Corren J (2011) The relationship of rhinitis and asthma, sinusitis, food allergy, and eczema. Immunol Allergy Clin North Am 31: 481–491.
11. Asher MI, Montefort S, Björkstén B, Lai CK, Strachan DP, et al. (2006) Worldwide time trends in the prevalence of symptoms of asthma, allergic rhinoconjunctivitis, and eczema in childhood: ISAAC Phases One and Three repeat multicountry cross-sectional surveys. Lancet 368: 733–743.
12. Ghouri N, Hippisley-Cox J, Newton J, Sheikh A (2008) Trends in the epidemiology and prescribing of medication for allergic rhinitis in England. J R Soc Med 101: 466–72.
13. Mosges R, Klimek L (2007) Today's allergic rhinitis patients are different: new factors that may play a role. Allergy 62: 969–975.
14. Lannerö E, Wickman M, van Hage M, Bergström A, Pershagen G, et al. (2008) Exposure to environmental tobacco smoke and sensitisation in children. Thorax 63: 172–176.
15. Diaz-Sanchez D, Rumold R, Gong H Jr (2006) Challenge with environmental tobacco smoke exacerbates allergic airway disease in human beings. J Allergy Clin Immunol 118: 441–446.
16. Peden D, Reed CE (2010) Environmental and occupational allergies. J Allergy Clin Immunol 125(2 Suppl 2): S150–S160.
17. Warren CW, Jones NR, Peruga A, Chauvin J, Baptiste JP, et al. (2008) Global youth tobacco surveillance, 2000–2007. MMWR Surveill Summ 57: 1–28.
18. Murin S, Rafii R, Bilello K (2011) Smoking and smoking cessation in pregnancy. Clin Chest Med 32: 75–91.
19. Cakir E, Ersu R, Uyan ZS, Oktem S, Varol N, et al. (2010) The prevalence and risk factors of asthma and allergic diseases among working adolescents. Asian Pac J Allergy Immunol 28: 122–129.
20. Lee CH, Chuang HY, Hong CH, Huang SK, Chang YC, et al. (2011) Lifetime exposure to cigarette smoking and the development of adult-onset atopic dermatitis. Br J Dermatol 164: 483–489.
21. Bendtsen P, Grønbaek M, Kjaer SK, Munk C, Linneberg A, et al. (2008) Alcohol consumption and the risk of self-reported perennial and seasonal allergic rhinitis in young adult women in a population-based cohort study. Clin Exp Allergy 38: 1179–1185.
22. Ludvigsson JF, Mostrom M, Ludvigsson J, Duchen K (2005) Exclusive breastfeeding and risk of atopic dermatitis in some 8300 infants. Pediatr Allergy Immunol 16: 201–208.
23. Metsälä J, Lundqvist A, Kaila M, Gissler M, Klaukka T, et al. (2010) Maternal and perinatal characteristics and the risk of cow's milk allergy in infants up to 2 years of age: a case-control study nested in the Finnish population. Am J Epidemiol 171: 1310–1316.
24. McKeever TM, Lewis SA, Smith C, Collins J, Heatlie H, et al. (2001). Siblings, multiple births, and the incidence of allergic disease: a birth cohort study using the West Midlands general practice research database. Thorax 56: 758–762.
25. Wang IJ, Guo YL, Lin TJ, Chen PC, Wu YN (2010) GSTM1, GSTP1, prenatal smoke exposure, and atopic dermatitis. Ann Allergy Asthma Immunol 105: 124–129.
26. Tariq SM, Matthews SM, Hakim EA, Stevens M, Arshad SH, et al. (1998) The prevalence of and risk factors for atopy in early childhood: a whole population birth cohort study. J Allergy Clin Immunol 101: 587–593.
27. Burke H, Leonardi-Bee J, Hashim A, Pine-Abata H, Chen Y, et al. (2012) Prenatal and passive smoke exposure and incidence of asthma and wheeze: systematic review and meta-analysis. Pediatrics 129: 735–744.
28. Baena-Cagnani CE, Gómez RM, Baena-Cagnani R, Canonica GW (2009) Impact of environmental tobacco smoke and active tobacco smoking on the development and outcomes of asthma and rhinitis. Curr Opin Allergy Clin Immunol 9: 136–140.
29. Pattenden S, Antova T, Neuberger M, Nikiforov B, De Sario M, et al. (2006) Parental smoking and children's respiratory health: independent effects of prenatal and postnatal exposure. Tob Control 15: 294–301.
30. Strachan DP, Cook DG (1998) Health effects of passive smoking. 5. Parental smoking and allergic sensitisation in children. Thorax 53: 117–123.
31. Freiman A, Bird G, Metelitsa AI, Barankin B, Lauzon GJ (2004) Cutaneous effects of smoking. J Cutan Med Surg 8: 415–423.
32. Just-Sarobé M (2008) Smoking and the skin. Actas Dermosifiliogr 99: 173–184.
33. National Institute for Public Health and the Environment (2010) Risk factors for food allergy. Bilthoven (The Netherlands): RIVM Report 340007001.
34. Wells G, Shea B, O'Connell D, Peterson J, Welch V, et al. (2012) The Newcastle-Ottawa scale (NOS) for assessing the quality of nonrandomised studies in meta-analyses. Ottawa Health Research Institute website. Available: http://www.ohri.ca/programs/clinical_epidemiology/oxford.asp. Accessed 16 August 2012.
35. Rothman KJ, Greenland S, Lash TL (2008) Measure of effect and measures of association. Modern epidemiology. 3rd edition. Philadelphia: Lippincott, Williams and Wilkins. P61.
36. Takkouche B, Cadarso-Suarez C, Spiegelman D (1999) Evaluation of old and new tests of heterogeneity in epidemiologic meta-analysis. Am J Epidemiol 150: 206–215.
37. Egger M, Davey Smith G, Schneider M, Minder C (1997) Bias in meta-analysis detected by a simple, graphical test. Br Med J 315: 629–634.
38. Costa-Bouzas J, Takkouche B, Cadarso-Suarez C, Spiegelman D (2001) HEpiMA: software for the identification of heterogeneity in meta-analysis. Comput Methods Programs Biomed 64: 101–107.
39. Ozasa K, Takenaka H, Takagi N, Aoike A, Kawai K (1995) A case-control study of risk factors for Japanese cedar pollinosis. Jpn J Hyg 50: 622–630.
40. Lin SY, Reh DD, Clipp S, Irani L, Navas-Acien A (2011) Allergic rhinitis and secondhand tobacco smoke: a population-based study. Am J Rhinol Allergy 25: e66–e71.
41. Miyake Y, Tanaka K, Arakawa M (2011) Case-control study of IL13 polymorphisms, smoking, and rhinoconjunctivitis in Japanese women: the Kyushu Okinawa Maternal and Child Health Study. BMC Med Genet 12: 143.
42. Wright AL, Holberg CJ, Halonen M, Martinez FD, Morgan W, et al. (1994) Epidemiology of physician-diagnosed allergic rhinitis in childhood. Pediatrics 94: 895–901.
43. Annesi-Maesano I, Oryszczyn M-P, Neukirch F, Kauffmann F (1997) Relationship of upper airway disease to tobacco smoking and allergic markers: a cohort study of men followed up for 5 years. Int Arch Allergy Immunol 114: 193–201.
44. Lewis SA, Britton JR (1998) Consistent effects of high socioeconomic status and low birth order, and the modifying effect of maternal smoking on the risk of allergic disease during childhood. Respir Med 92: 1237–1244.
45. Shaheen SO, Sterne JAC, Montgomery SM, Azima H (1999) Birth weight, body mass index and asthma in young adults. Thorax 54: 396–402.
46. Bergmann RL, Edenharter G, Bergmann KE, Lau S, Wahn U (2000) Socioeconomic status is a risk factor for allergy in parents but not in their children. Clin Exp Allergy 30: 1740–1745.
47. Tariq SM, Hakim EA, Matthews SM, Arshad SH (2000) Influence of smoking on asthmatic symptoms and allergen sensitisation in early childhood. Postgrad Med J 76: 694–699.
48. Magnusson LL, Olesen AB, Wennborg H, Olsen J (2005) Wheezing, asthma, hayfever, and atopic eczema in childhood following exposure to tobacco smoke in fetal life. Clin Exp Allergy 35: 1550–1556.
49. Johansson A, Ludvigsson J, Hermansson G (2008) Adverse health effects related to tobacco smoke exposure in a cohort of three-year olds. Acta Pædiatr 97: 354–357.
50. Nagata C, Nakamura K, Fujii K, Kawachi T, Takatsuka N, et al. (2008) Smoking and risk of cedar pollinosis in Japanese men and women. Int Arch Allergy Immunol 147: 117–124.
51. Keil T, Lau S, Roll S, Gruber C, Nickel R, et al. (2009) Maternal smoking increases risk of allergic sensitization and wheezing only in children with allergic predisposition: longitudinal analysis from birth to 10 years. Allergy 64: 445–451.
52. Codispoti CD, Levin L, LeMasters GK, Ryan P, Reponen T, et al. (2010) Breast-feeding, aeroallergen sensitization, and environmental exposures during infancy are determinants of childhood allergic rinitis. J Allergy Clin Immunol 125: 1054–1060.
53. Bakke P, Gulsvik A, Eide GE (1990) Hay fever, eczema and urticaria in southwest Norway. Lifetime prevalences and association with sex, age, smoking habits, occupational airborne exposures and respiratory symptoms. Allergy 45: 515–522.
54. Leuenberger P, Schwartz J, Ackermann-Liebrich U, Blaser K, Bolognini G, et al. (1994) Passive smoking exposure in adults and chronic respiratory

symptoms (SAPALDIA Study) Swiss Study on Air Pollution and Lung Diseases in Adults, SAPALDIA Team. Am J Respir Crit Care Med 150: 1222–1228.
55. Ng TP, Tan WC (1994) Epidemiology of allergic rhinitis and its associated risk factors in Singapore. Int J Epidemiol 23: 553–558.
56. Moyes CD, Waldon J, Ramadas D, Crane J, Pearce N (1995) Respiratory symptoms and environmental factors in schoolchildren in the Bay of Plenty. N Z Med J 108: 358–361.
57. Wüthrich B, Schindler C, Medici TC, Zellweger JP, Leuenberger P (1996) IgE levels, atopy markers and hay fever in relation to age, sex and smoking status in a normal adult Swiss population. SAPALDIA (Swiss Study on Air Pollution and Lung Diseases in Adults) Team. Int Arch Allergy Immunol 111: 396–402.
58. Min Y-G, Jung H-W, Kim HS, Park SK, Yoo KY (1997) Prevalence and risk factors for perennial allergic rhinitis in Korea: results of a nationwide survey. Clin Otolaryngol 22: 139–144.
59. Siracusa A, Marabini A, Sensi L, Bacoccoli R, Ripandelli A, et al. (1997) Prevalence of asthma and rhinitis in Perugia, Italy. Monaldi Arch Chest Dis 52: 434–439.
60. Austin JB, Russell G (1997) Wheeze, cough, atopy, and indoor environment in the Scottish Highlands. Arch Dis Child 76: 22–26.
61. Farooqi IS, Hopkin JM (1998) Early childhood infection and atopic disorder. Thorax 53: 927–32.
62. Lam TH, Chung SF, Betson CL, Wong CM, Hedley AJ (1998) Respiratory symptoms due to active and passive smoking in junior secondary school students in Hong Kong. Int J Epidemiol 27: 41–48.
63. Ponsonby AL, Couper D, Dwyer T, Carmichael A (1998) Cross sectional study of the relation between sibling number and asthma, hay fever, and eczema. Arch Dis Child 79: 328–333.
64. Montefort S, Lenicker HM, Caruna S, Agius Muscat H (1998) Asthma, rhinitis and eczema in Maltese 13–15 year-old schoolchildren – prevalence, severity and associated factors [ISAAC]. International Study of Asthma and Allergies in Childhood. Clin Exp Allergy 28: 1089–1099.
65. Duhme H, Weiland SK, Rudolph P, Wienke A, Kramer A, et al. (1998) Asthma and allergies among children in West and East Germany: a comparison between Münster and Greifswald using the ISAAC phase I protocol. International Study of Asthma and Allergies in Childhood. Eur Respir J 11: 840–847.
66. Burr ML, Anderson HR, Austin JB, Harkins LS, Kaur B, et al. (1999) Respiratory symptoms and home environment in children: a national survey. Thorax 54: 27–32.
67. Dotterud LK, Falk ES (1999) Atopic disease among adults in northern Russia, an area with heavy air pollution. Acta Derm Venereol 79: 448–450.
68. Keleş N, Ilicali C, Değer K (1999) The effects of different levels of air pollution on atopy and symptoms of allergic rhinitis. Am J Rhinol 13: 185–190.
69. Plaschke PP, Janson C, Norrman E, Björnsson E, Ellbjär S, et al. (2000) Onset and remission of allergic rhinitis and asthma and the relationship with atopic sensitization and smoking. Am J Respir Crit Care Med 162: 920–924.
70. Zacharasiewicz A, Zidek T, Haidinger G, Waldhor T, Vutuc C (2000) Symptoms suggestive of atopic rhinitis in children aged 6–9 years and the indoor environment. Allergy 55: 945–950.
71. Upton MN, McConnachie A, McSharry C, Hart CL, Smith GD, et al. (2000) Intergenerational 20 year trends in the prevalence of asthma and hay fever in adults: the Midspan family study surveys of parents and offspring. BMJ 321: 88–92.
72. Ozdemir N, Uçgun I, Metintas S, Kolsuz M, Metintas M (2000) The prevalence of asthma and allergy among university freshmen in Eskisehir, Turkey. Respir Med 94: 536–541.
73. Hjern A, Hedberg A, Haglund B, Rosén M (2001) Does tobacco smoke prevent atopic disorders? A study of two generations of Swedish residents. Clin Exp Allergy 31: 908–914.
74. Janson C, Chinn S, Jarvis D, Zock JP, Torén K, et al. (2001) Effect of passive smoking on respiratory symptoms, bronchial responsiveness, lung function, and total serum IgE in the European Community Respiratory Health Survey: a cross-sectional study. Lancet 358: 2103–2109.
75. Simpson BM, Custovic A, Simpson A, Hallam CL, Walsh D, et al. (2001) NAC Manchester asthma and allergy study (NACMAAS): risk factors for asthma and allergic disorders in adults. Clin Exp Allergy 31: 391–399.
76. Dotterud LK, Odland JO, Falk ES (2001) Atopic diseases among schoolchildren in Nikel, Russia, an Arctic area with heavy air pollution. Acta Derm Venereol 81: 198–201.
77. Kalyoncu AF, Demir AU, Ozcakar B, Bozkurt B, Artvinli M (2001) Asthma and allergy in Turkish university students: Two cross-sectional surveys 5 years apart. Allergol Immunopathol (Madr) 29: 264–271.
78. Lee SI, Shin MH, Lee HB, Lee JS, Son BK, et al. (2001) Prevalences of symptoms of asthma and other allergic diseases in Korean children: a nationwide questionnaire survey. J Korean Med Sci 16: 155–164.
79. Stazi MA, Sampogna F, Montagano G, Grandolfo ME, Couilliot MF, et al. (2002) Early life factors related to clinical manifestations of atopic disease but not to skin-prick test positivity in young children. Pediatr Allergy Immunol 13: 105–112.
80. Peroni DG, Piacentini GL, Alfonsi L, Zerman L, Di Blasiw P, et al. (2003) Rhinitis in pre-school children: prevalence, association with allergic diseases and risk factors. Clin Exp Allergy 33: 1349–1354.
81. Barraza Villarreal A, Sanín Aguirre LH, Téllez Rojo MM, Lacasaña Navarro M, Romieu I (2003) Risk factors for asthma in school children from Ciudad Juárez, Chihuahua. J Asthma 40: 413–423.
82. Monteil MA, Joseph G, Chang Kit C, Wheeler G, Antoine RM (2004) Smoking at home is strongly associated with symptoms of asthma and rhinitis in children of primary school age in Trinidad and Tobago. Rev Panam Salud Publica 16: 193–198.
83. Lee SL, Wong W, Lau YL (2004) Increasing prevalence of allergic rhinitis but not asthma among children in Hong Kong from 1995 to 2001 (Phase 3 International Study of Asthma and Allergies in Childhood). Pediatr Allergy Immunol 15: 72–78.
84. Krämer U, Lemmen CH, Behrendt H, Link E, Schäfer T, et al. (2004) The effect of environmental tobacco smoke on eczema and allergic sensitization in children. Br J Dermatol 150: 111–118.
85. Demir AU, Karakaya G, Bozkurt B, Sekerel BE, Kalyoncu AF (2004) Asthma and allergic diseases in schoolchildren: third cross-sectional survey in the same primary school in Ankara, Turkey. Pediatr Allergy Immunol 15: 531–538.
86. Miyake Y, Yura A, Iki M (2004) Cross-sectional study of allergic disorders in relation to familial factors in Japanese adolescents. Acta Paediatr 93: 380–385.
87. Annesi-Maesano I, Oryszczyn MP, Raherison C, Kopferschmitt C, Pauli G, et al. (2004) Increased prevalence of asthma and allied diseases among active adolescent tobacco smokers after controlling for passive smoking exposure. A cause for concern? Clin Exp Allergy 34: 1017–1023.
88. De S, Fenton JE, Jones AS, Clarke RW (2005) Passive smoking, allergic rhinitis and nasal obstruction in children. J Laryngol Otol 119: 955–957.
89. Topp R, Thefeld W, Wichmann HE, Heinrich J (2005) The effect of environmental tobacco smoke exposure on allergic sensitization and allergic rhinitis in adults. Indoor Air 15: 222–227.
90. Maziak W, Kenneth D, Ward KD, Rastam S, Mzayek F, et al. (2005) Extent of exposure to environmental tobacco smoke (ETS) and its dose-response relation to respiratory health among adults. Respir Res 6: 13.
91. Miyake Y, Miyamoto S, Ohya Y, Sasaki S, Matsunaga I, et al. (2005) Association of active and passive smoking with allergic disorders in pregnant Japanese women: baseline data from the Osaka Maternal and Child Health Study. Ann Allergy Asthma Immunol 94: 644–651.
92. Bugiani M, Carosso A, Migliore E, Piccioni P, Corsico A, et al. (2005) ISAYA (ECRHS Italy) Study Group. Allergic rhinitis and asthma comorbidity in a survey of young adults in Italy. Allergy 60: 165–70.
93. Obihara CC, Marais BJ, Gie RP, Potter P, Bateman ED, et al. (2005) The association of prolonged breastfeeding and allergic disease in poor urban children. Eur Respir J 25: 970–977.
94. Strumylaite L, Kregzdyte R, Vaitkaitiene E (2005) Pasyvus rukymas ir vaiku kvepavimo sutrikimai [Passive smoking and respiratory health of children]. Medicina (Kaunas) 41: 348–354.
95. Lund VJ, Preziosi P, Hercberg S, Hamoir M, Dubreuil C, et al. (2006) Yearly incidence of rhinitis, nasal bleeding, and other nasal symptoms in mature women. Rhinology 44: 26–31.
96. Kurosaka F, Nakatani Y, Terada T, Tanaka A, Ikeuchi H, et al. (2006) Current cat ownership may be associated with the lower prevalence of atopic dermatitis, allergic rhinitis, and Japanese cedar pollinosis in schoolchildren in Himeji, Japan. Pediatr Allergy Immunol 17: 22–28.
97. Sakar A, Yorgancioglu A, Dinc G, Yuksel H, Celik P, et al. (2006) The prevalence of asthma and allergic symptoms in Manisa, Turkey (A western city from a country bridging Asia and Europe). Asian Pac J Allergy Immunol 24: 17–25.
98. Ho SY, Lam TH, Chung SF, Lam TP (2007) Cross-sectional and prospective associations between passive smoking and respiratory symptoms at the workplace. Ann Epidemiol 17: 126–131.
99. Horak E, Morassa B, Ulmerb H (2007) Association between environmental tobacco smoke exposure and wheezing disorders in Austrian preschool children. Swiss Med Wkly 137: 608–613.
100. Ebbert JO, Croghan IT, Schroeder DR, Murawski J, Hurt RD (2007) Association between respiratory tract diseases and secondhand smoke exposure among never smoking flight attendants: a cross-sectional survey. Environ Health 6: 28.
101. Tanaka K, Miyake Y, Arakawa M, Sasaki S, Ohya Y (2007) Prevalence of asthma and wheeze in relation to passive smoking in Japanese children. Ann Epidemiol 17: 1004–1010.
102. Zuraimi MS, Tham KW, Chew FT, Ooi PL, David K (2008) Home exposures to environmental tobacco smoke and allergic symptoms among young children in Singapore. Int Arch Allergy Immunol 146: 57–65.
103. Foliaki S, Annesi-Maesano I, Tuuau-Potoi N, Waqatakirewa L, Cheng S, et al. (2008) Risk factors for symptoms of childhood asthma, allergic rhinoconjunctivitis and eczema in the Pacific: an ISAAC Phase III study. Int J Tuberc Lung Dis 12: 799–780.
104. Gómez R, Teijeiro A, Zernotti M, Canonica G, Mimessi G, et al. (2008) Smoking is a risk factor for having rhinitis in adolescents. Allergy 63: 419.
105. Kabir Z, Manning PJ, Holohan J, Keogan S, Goodman PG, et al. (2009) Second-hand smoke exposure in cars and respiratory health effects in children. Eur Respir J 34: 629–633.
106. Brescianini S, Brunetto B, Iacovacci P, D'Ippolito C, Alberti G, et al. (2009) Prevalence of self-perceived allergic diseases and risk factors in Italian adolescents. Pediatr Allergy Immunol 20: 578–84.

107. Musharrafieh U, Al-Sahab B, Zaitoun F, El-Hajj MA, Ramadan F, et al. (2009) Prevalence of asthma, allergic rhinitis and eczema among Lebanese adolescents. J Asthma 46: 382–87.
108. González-Díaz SN, Del Río-Navarro BE, Pietropaolo-Cienfuegos DR, Escalante-Domínguez AJ, García-Almaraz RG, et al. (2010) Factors associated with allergic rhinitis in children and adolescents from northern Mexico: International Study of Asthma and Allergies in Childhood Phase IIIB. Allergy Asthma Proc 31: 53–62.
109. Bedolla-Barajas M, Cuevas-Ríos G, García-Barboza E, Barrera-Zepeda AT, Morales-Romero J (2010) Prevalencia y factores asociados a la rinitis alérgica en escolares de Ciudad Guzmán, Mexico. [Prevalence and factors associated to allergic rhinitis among schoolchildren of Ciudad Guzmán, Mexico] Rev Invest Clin 62: 244–251.
110. Wang HY, Pizzichini MM, Becker AB, Duncan JM, Ferguson AC, et al. (2010) Disparate geographic prevalences of asthma, allergic rhinoconjunctivitis and atopic eczema among adolescents in five Canadian cities. Pediatr Allergy Immunol 21: 867–877.
111. Vlaski E, Stavric K, Seckova L, Kimovska M, Isjanovska R (2011) Do household tobacco smoking habits influence asthma, rhinitis and eczema among 13–14 year-old adolescents? Allergol Immunopathol 39: 39–44.
112. Virkkula P, Liukkonen K, Suomalainen AK, Aronen ET, Kirjavainen T, et al. (2011) Parental smoking, nasal resistance and rhinitis in children. Acta Paediatr 100: 1234–1238.
113. Håkansson K, von Buchwald C, Thomsen SF, Thyssen JP, Backer V, et al. (2011) Nonallergic rhinitis and its association with smoking and lower airway disease: a general population study. Am J Rhinol Allergy 25: 25–29.
114. Chen BY, Chan CC, Han YY, Wu HP, Guo YL (2012) The risk factors and quality of life in children with allergic rhinitis in relation to seasonal attack patterns. Paediatr Perinat Epidemiol 26: 146–155.
115. Peñaranda A, Aristizabal G, Garcia E, Vasquez C, Rodriguez-Martinez CE, et al. (2012) Allergic rhinitis and associated factors in schoolchildren from Bogota, Colombia. Rhinology 50: 122–128.
116. Tanaka K, Miyake Y, Arakawa M (2012) Smoking and prevalence of allergic disorders in Japanese pregnant women: baseline data from the Kyushu Okinawa Maternal and Child Health Study. Environ Health 11: 15.
117. Montefort S, Ellul P, Montefort M, Caruana S, Grech V, et al. (2012) The effect of cigarette smoking on allergic conditions in Maltese children (ISAAC). Pediatr Allergy Immunol 23: 472–478.
118. Mitchell EA, Beasley R, Keil U, Montefort S, Odhiambo J, et al. (2012) The association between tobacco and the risk of asthma, rhinoconjunctivitis and eczema in children and adolescents: analyses from Phase Three of the ISAAC programme. Thorax 67: 941–949.
119. Mills CM, Srivastava ED, Harvey IM, Swift GL, Newcombe RG, et al. (1994) Cigarette smoking is not a risk factor in atopic dermatitis. Int J Dermatol 33: 33–34.
120. Yang CY, Cheng MF, Hsieh YL (2000) Effects of indoor environmental factors on risk for atopic eczema in a subtropical area. J Toxicol Environ Health A 61: 245–253.
121. Purvis DJ, Thompson JM, Clark PM, Robinson E, Black PN, et al. (2005) Risk factors for atopic dermatitis in New Zealand children at 3.5 years of age. Br J Dermatol 152: 742–749.
122. Haileamlak A, Dagoye D, Williams H, Venn AJ, Hubbard R, et al. (2005) Early life risk factors for atopic dermatitis in Ethiopian children. J Allergy Clin Immunol 115: 370–376.
123. Sebõk B, Schneider I, Harangi F (2006) Primary Care Paediatricians in Baranya County. Familiar and environmental factors influencing atopic dermatitis in the childhood. J Eur Acad Dermatol Venereol 20: 418–422.
124. Miyake Y, Tanaka K, Arakawa M (2011) IL13 genetic polymorphisms, smoking, and eczema in women: a case-control study in Japan. BMC Med Genet 12: 142.
125. Burr ML, Miskelly FG, Butland BK, Merrett TG, Vaughan-Williams E (1989) Environmental factors and symptoms in infants at high risk of allergy. J Epidemiol Community Health 43: 125–132.
126. Zeiger RS, Heller S (1995) The development and prediction of atopy in high-risk children: follow-up at age seven years in a prospective randomized study of combined maternal and infant food allergen avoidance. J Allergy Clin Immunol 95: 1179–1190.
127. Olesen AB, Ellingsen AR, Olesen H, Juul S, Thestrup-Pedersen K (1997) Atopic dermatitis and birth factors: historical follow up by record linkage. BMJ 314: 1003–1008.
128. Bergmann RL, Diepgen TL, Kuss O, Bergmann KE, Kujat J, et al. (2002) Breastfeeding duration is a risk factor for atopic eczema. Clin Exp Allergy 32: 205–209.
129. Kerkhof M, Koopman LP, van Strien RT, Wijga A, Smit HA, et al. (2003) Risk factors for atopic dermatitis in infants at high risk of allergy: the PIAMA study. Clin Exp Allergy 33: 1336–1341.
130. Linneberg A, Simonsen JB, Petersen J, Stensballe LG, Benn CS (2006) Differential effects of risk factors on infant wheeze and atopic dermatitis emphasize a different etiology. J Allergy Clin Immunol 117: 184–189.
131. Lerbaek A, Kyvik KO, Ravn H, Menné T, Agner T (2007) Incidence of hand eczema in a population-based twin cohort: genetic and environmental risk factors. Br J Dermatol 157: 552–557.
132. Noakes P, Taylor A, Hale J, Breckler L, Richmond P, et al. (2007) The effects of maternal smoking on early mucosal immunity and sensitization at 12 months of age. Pediatr Allergy Immunol 18: 118–127.
133. Sariachvili M, Droste J, Dom S, Wieringa M, Vellinga A, et al. (2007) Is breast feeding a risk factor for eczema during the first year of life? Pediatr Allergy Immunol 18: 410–417.
134. Tanaka K, Miyake Y, Sasaki S, Ohya Y, Hirota Y, et al. (2008) Maternal smoking and environmental tobacco smoke exposure and the risk of allergic diseases in Japanese infants: the Osaka Maternal and Child Health Study. J Asthma 45: 833–838.
135. Böhme M, Kull I, Bergström A, Wickman M, Nordvall L, et al. (2010) Parental smoking increases the risk for eczema with sensitization in 4-year-old children. J Allergy Clin Immunol 125: 941–943.
136. Jedrychowski W, Perera F, Maugeri U, Mrozek-Budzyn D, Miller RL, et al. (2011) Effects of prenatal and perinatal exposure to fine air pollutants and maternal fish consumption on the occurrence of infantile eczema. Int Arch Allergy Immunol 155: 275–281.
137. Edman B (1988) Palmar eczema: a pathogenetic role for acetylsalicylic acid, contraceptives and smoking? Acta Derm Venereol 68: 402–407.
138. Volkmer RE, Ruffin RE, Wigg NR, Davies N (1995) The prevalence of respiratory symptoms in South Australian preschool children. II. Factors associated with indoor air quality. J Paediatr Child Health 31: 116–120.
139. Liss GM, Sussman GL, Deal K, Brown S, Cividino M, et al. (1997) Latex allergy: epidemiological study of 1351 hospital workers. Occup Environ Med 54: 335–342.
140. Schäfer T, Dirschedl P, Kunz B, Ring J, Uberla K (1997) Maternal smoking during pregnancy and lactation increases the risk for atopic eczema in the offspring. J Am Acad Dermatol 36: 550–556.
141. Linneberg A, Nielsen NH, Menné T, Madsen F, Jørgensen T (2003) Smoking might be a risk factor for contact allergy. J Allergy Clin Immunol 111: 980–984.
142. Montnemery P, Nihlén U, Göran Löfdahl C, Nyberg P, Svensson A (2003) Prevalence of self-reported eczema in relation to living environment, socio-economic status and respiratory symptoms assessed in a questionnaire study. BMC Dermatol 3: 4.
143. Yemaneberhan H, Flohr C, Lewis SA, Bekele Z, Parry E, et al. (2004) Prevalence and associated factors of atopic dermatitis symptoms in rural and urban Ethiopia. Clin Exp Allergy 34: 779–785.
144. Heudorf U, Schümann M, Angerer J, Exner M (2005) Dermal and bronchial symptoms in children: are they caused by PAH containing parquet glue or by passive smoking? Int Arch Occup Environ Health 78: 655–662.
145. Montnemery P, Nihlén U, Löfdahl CG, Nyberg P, Svensson A (2005) Prevalence of hand eczema in an adult Swedish population and the relationship to risk occupation and smoking. Acta Derm Venereol 85: 429–432.
146. Dotterud LK, Smith-Sivertsen T (2007) Allergic contact sensitization in the general adult population: a population-based study from Northern Norway. Contact Dermatitis 56: 10–15.
147. Al-Sahab B, Atoui M, Musharrafieh U, Zaitoun F, Ramadan F, et al. (2008) Epidemiology of eczema among Lebanese adolescents. Int J Public Health 53: 260–267.
148. Ergin S, Ozşahin A, Erdoğan BS, Aktan S, Zencir M (2008) Epidemiology of atopic dermatitis in primary schoolchildren in Turkey. Pediatr Dermatol 25: 399–401.
149. Suárez-Varela M, García-Marcos L, Kogan MD, Llopis González A, Martínez Gimeno A, et al. (2008) Parents' smoking habit and prevalence of atopic eczema in 6–7 and 13–14 year-old schoolchildren in Spain. ISAAC phase III. Allergol Immunopathol (Madr) 36: 336–442.
150. Attwa E, el-Laithy N (2009) Contact dermatitis in car repair workers. J Eur Acad Dermatol Venereol 23: 138–145.
151. Meding B, Alderling M, Albin M, Brisman J, Wrangsjö K (2009) Does tobacco smoking influence the occurrence of hand eczema? Br J Dermatol 160: 514–518.
152. Lipińska KI, Elgalal A, Kuna P (2009) Epidemiologia atopowego zapalenia skory w populacji ogolnej mieszkancow wojewodztwa lodzkiego [Epidemiology of atopic dermatitis in general population of Lodz province's citizens]. Pneumonol Alergol Pol 77: 145–151.
153. Xepapadaki P, Manios Y, Liarigkovinos T, Grammatikaki E, Douladiris N, et al. (2009) Association of passive exposure of pregnant women to environmental tobacco smoke with asthma symptoms in children. Pediatr Allergy Immunol 20: 423–429.
154. Röhrl K, Stenberg B (2010) Lifestyle factors and hand eczema in a Swedish adolescent population. Contact Dermatitis 62: 170–176.
155. Thyssen JP, Linneberg A, Menné T, Nielsen NH, Johansen JD (2010) The effect of tobacco smoking and alcohol consumption on the prevalence of self-reported hand eczema: a cross-sectional population-based study. Br J Dermatol 162: 619–626.
156. Meding B, Alderling M, Wrangsjö K (2010) Tobacco smoking and hand eczema: a population-based study. Br J Dermatol 163: 752–756.
157. Yang YW, Chen YH, Huang YH (2011) Cigarette smoking may modify the risk of depression in eczema among adults: a preliminary study using NHANES 2005–2006. J Eur Acad Dermatol Venereol 25: 1048–1053.
158. Civelek E, Sahiner UM, Yüksel H, Boz AB, Orhan F, et al. (2011) Prevalence, burden, and risk factors of atopic eczema in schoolchildren aged 10–11 years: a national multicenter study. J Investig Allergol Clin Immunol 21: 270–277.

159. Dei-Cas P, Acuña MK, Dei-Cas I (2011) Atopic dermatitis in children: a comparative survey among 2 age groups. Rev Chil Pediatr 82: 410–418.
160. Apfelbacher CJ, Diepgen TL, Schmitt J (2011) Determinants of eczema: population-based cross-sectional study in Germany. Allergy 66: 206–213.
161. Park H, Kim K (2011) Association of blood mercury concentrations with atopic dermatitis in adults: a population-based study in Korea. Environ Res 111: 573–578.
162. Berglind IA, Alderling M, Meding B (2011) Life-style factors and hand eczema. Br J Dermatol 165: 568–575.
163. Breunig JA, de Almeida HL Jr, Duquia RP, Souza PR, Staub HL (2012) Scalp seborrheic dermatitis: prevalence and associated factors in male adolescents. Int J Dermatol 51: 46–49.
164. Yi O, Kwon HJ, Kim H, Ha M, Hong SJ, et al. (2012) Effect of environmental tobacco smoke on atopic dermatitis among children in Korea. Environ Res 113: 40–45.
165. Rönmark EP, Ekerljung L, Lötvall J, Wennergren G, Rönmark E, et al. (2012) Eczema among adults: prevalence, risk factors and relation to airway diseases. Results from a large-scale population survey in Sweden. Br J Dermatol 166: 1301–1308.
166. Kavaliūnas A (2011) Padidėjusio jautrumo maisto produktams ir alergijos maistui paplitimas tarp Vilniaus miesto gyventojų [The prevalence of adverse reactions to food and food allergy among Vilnius city (Lithuania) inhabitants] [dissertation]. Vilnius: Institute of Public Health, Vilnius University.
167. Kulig M, Luck W, Lau S, Niggemann B, Bergmann R, et al. (1999) Effect of pre- and postnatal tobacco smoke exposure on specific sensitization to food and inhalant allergens during the first 3 years of life. Multicenter Allergy Study Group, Germany. Allergy 54: 220–228.
168. Dubakienė R, Šurkienė G, Stukas R, Pirmaitytė-Vilesko J, Kavaliūnas A (2008) Food allergies among 5th–9th grade schoolchildren in Vilnius (Lithuania). Ekologija 54: 1–4.
169. Taylor B, Wadsworth J, Golding J, Butler N (1983) Breast feeding, eczema, asthma, and hayfever. J Epidemiol Community Health 37: 95–99.
170. Butland BK, Strachan DP, Lewis S, Bynner J, Butler N, et al. (1997) Investigation into the increase in hay fever and eczema at age 16 observed between the 1958 and 1970 British birth cohorts. BMJ 315: 717–721.
171. Arshad SH, Stevens M, Hide DW (1993) The effect of genetic and environmental factors on the prevalence of allergic disorders at the age of two years. Clin Exp Allergy 23: 504–511.
172. Biagini JM, LeMasters GK, Ryan PH, Levin L, Reponen T, et al. (2006) Environmental risk factors of rhinitis in early infancy. Pediatr Allergy Immunol 17: 278–284.
173. Reh DD, Lin SY, Clipp SL, Irani L, Alberg AJ, et al. (2009) Secondhand tobacco smoke exposure and chronic rhinosinusitis: a population-based case-control study. Am J Rhinol Allergy 23: 562–567.
174. Merrett TG, Burr ML, Butland BK, Merrett J, Miskelly FG, et al. (1988) Infant feeding and allergy: 12-month prospective study of 500 babies born into allergic families. Ann Allergy 61: 13–20.
175. Dei-Cas I, Dei-Cas P, Acuña K (2009) Atopic dermatitis and risk factors in poor children from Great Buenos Aires, Argentina. Clin Exp Dermatol 34: 299–303.
176. Wang IJ, Hsieh WS, Wu KY, Guo YL, Hwang YH, et al. (2008) Effect of gestational smoke exposure on atopic dermatitis in the offspring. Pediatr Allergy Immunol 19: 580–586.
177. Dotterud LK, Odland JØ, Falk ES (2004) Atopic dermatitis and respiratory symptoms in Russian and northern Norwegian school children: a comparison study in two arctic areas and the impact of environmental factors. J Eur Acad Dermatol Venereol 18: 131–136.
178. Dotterud LK, Odland JO, Falk ES (2000) Atopic diseases among adults in the two geographically related arctic areas Nikel, Russia and Sør-Varanger, Norway: possible effects of indoor and outdoor air pollution. J Eur Acad Dermatol Venereol 14: 107–111.
179. Kulig M, Luck W, Wahn U (1999) The association between pre- and postnatal tobacco smoke exposure and allergic sensitization during early childhood. Multicentre Allergy Study Group, Germany. Hum Exp Toxicol 18: 241–244.
180. Miyake Y, Ohya Y, Tanaka K, Yokoyama T, Sasaki S, et al. (2007) Home environment and suspected atopic eczema in Japanese infants: the Osaka Maternal and Child Health Study. Pediatr Allergy Immunol 18: 425–432.
181. Thyssen JP, Johansen JD, Menné T, Nielsen NH, Linneberg A (2010) Effect of tobacco smoking and alcohol consumption on the prevalence of nickel sensitization and contact sensitization. Acta Derm Venereol 90: 27–33.
182. Barbee RA, Halonen M, Kaltenborn WT, Burrows B (1991) A longitudinal study of respiratory symptoms in a community population sample. Correlations with smoking, allergen skin-test reactivity, and serum IgE. Chest 99: 20–26.
183. Thomsen SF, Ulrik CS, Porsbjerg C, Backer V (2006) Early life exposures and risk of atopy among Danish children. Allergy Asthma Proc 27: 110–114.
184. Larsson ML, Magnuson A, Montgomery SM (2005) Parental smoking and allergic sensitization in offspring defined by skin prick testing. Pediatr Allergy Immunol 16: 449–452.
185. Liptay S, Bauer CP, Grübl A, Franz R, Emmrich P (1991) Atopieentwicklung in der fruhen Kindheit–Pradisponierende Faktoren [Development of atopic disease in early childhood–predisposing factors]. Monatsschr Kinderheilkd 139: 130–135.
186. Wittig HJ, McLaughlin ET, Leifer KL, Belloit JD (1978) Risk factors for the development of allergic disease: analysis of 2,190 patient records. Ann Allergy 41: 84–88.
187. Linneberg A, Nielsen NH, Madsen F, Frølund L, Dirksen A, et al. (2001) Smoking and the development of allergic sensitization to aeroallergens in adults: a prospective population-based study. The Copenhagen Allergy Study. Allergy 56: 328–332.
188. Zetterström O, Osterman K, Machado L, Johansson SG (1981) Another smoking hazard: raised serum IgE concentration and increased risk of occupational allergy. BMJ 283: 1215–1217.
189. Bråbäck L, Kjellman NI, Sandin A, Björkstén B (2001) Atopy among schoolchildren in northern and southern Sweden in relation to pet ownership and early life events. Pediatr Allergy Immunol 12: 4–10.
190. Raherison C, Pénard-Morand C, Moreau D, Caillaud D, Charpin D, et al. (2008) Smoking exposure and allergic sensitization in children according to maternal allergies. Ann Allergy Asthma Immunol 100: 351–357.
191. Bakos N, Schöll I, Szalai K, Kundi M, Untersmayr E, et al. (2006) Risk assessment in elderly for sensitization to food and respiratory allergens. Immunol Lett 107: 15–21.
192. Harris-Roberts J, Robinson E, Waterhouse JC, Billings CG, Proctor AR, et al. (2009) Sensitization to wheat flour and enzymes and associated respiratory symptoms in British bakers. Am J Ind Med 52: 133–140.
193. Tsunoda K, Ohta Y, Shinogami M, Soda Y (1995) Does passive smoking affect the incidence of nasal allergies? Am J Public Health 85: 1019–1020.
194. Jeebhay MF, Robins TG, Miller ME, Bateman E, Smuts M, et al. (2008) Occupational allergy and asthma among salt water fish processing workers. Am J Ind Med 51: 899–910.
195. Angioni AM, Fanciulli G, Corchiat C (1989) Frequency of and risk factors for allergy in primary school children: results of a population Survey. Paediatr Perinat Epidemiol 3: 248–255.
196. Frank P, Morris J, Hazell M, Linehan M, Frank T (2006) Smoking, respiratory symptoms and likely asthma in young people: evidence from postal questionnaire surveys in the Wythenshawe Community Asthma Project (WYCAP). BMC Pulm Med 6: 10.
197. Guedes HTV, Souza LSF (2009) Exposure to maternal smoking in the first year of life interferes in breast-feeding protective effect against the onset of respiratory allergy from birth to 5 yr. Pediatr Allergy Immunol 20: 30–34.
198. Staikūniene J, Sakalauskas R (2003) Ziedadulkiu sukelto alerginio rinito ir bronchu astmos imunologines savybes bei rizikos veiksniai [The immunological parameters and risk factors for pollen-induced allergic rhinitis and asthma]. Medicina (Kaunas) 39: 244–253.
199. Dubakienė R, Vaicekauskaitė D, Židanavičiūtė J, Joneliūnienė I, Drąsutienė G, et al. (2006) Human ecology studies: the role of environmental factors in pregnancy. Ekologija 4: 18–21.
200. Woods RK, Abramson M, Raven JM, Bailey M, Weiner JM, et al. (1998) Reported food intolerance and respiratory symptoms in young adults. Eur Respir J 11: 151–155.
201. Huang SW (2007) Follow-up of children with rhinitis and cough associated with milk allergy. Pediatr Allergy Immunol 18: 81–85.
202. Pegas PN, Alves CA, Scotto MG, Evtyugina MG, Pio CA, et al. (2011) Factores de risco e prevalencia de asma e rinite em criancas em idade escolar em Lisboa [Risk factors and prevalence of asthma and rhinitis among schoolchildren in Lisbon]. Rev Port Pneumol 17: 109–116.
203. Gustafsson D, Andersson K, Fagerlund I, Kjellman NI (1996) Significance of indoor environment for the development of allergic symptoms in children followed up to 18 months of age. Allergy 51: 789–795.
204. Hagendorens MM, Bridts CH, Lauwers K, van Nuijs S, Ebo DG, et al. (2005) Perinatal risk factors for sensitization, atopic dermatitis and wheezing during the first year of life (PIPO study). Clin Exp Allergy 35: 733–740.
205. Lucas A, Brooke OG, Cole TJ, Morley R, Bamford MF (1990) Food and drug reactions, wheezing, and eczema in preterm infants. Arch Dis Child 65: 411–415.
206. Vessey MP, Painter R, Powell J (2000) Skin disorders in relation to oral contraception and other factors, including age, social class, smoking and body mass index. Findings in a large cohort study. Br J Dermatol 143: 815–820.
207. Girolomoni G, Abeni D, Masini C, Sera F, Ayala F, et al. (2003) The epidemiology of atopic dermatitis in Italian schoolchildren. Allergy 58: 420–425.
208. Dubakiene R, Rudzeviciene O, Butiene I, Sezaite I, Petronyte M, et al. (2012) Studies on early allergic sensitization in the Lithuanian birth cohort. Sci World J 2012: 909524.
209. Mitchell EA, Stewart AW, ISAAC Phase One Study Group (2001) International Study of Asthma and Allergy in Childhood. The ecological relationship of tobacco smoking to the prevalence of symptoms of asthma and other atopic diseases in children: the International Study of Asthma and Allergies in Childhood (ISAAC). Eur J Epidemiol 17: 667–673.
210. The Global Youth Tobacco Survey Collaborative Group (2002) Tobacco use among youth: a cross country comparison. Tob Control 11: 252–270.
211. King K, Martynenko M, Bergman MH, Liu YH, Winickoff JP, et al. (2009) Family composition and children's exposure to adult smokers in their homes. Pediatrics 123: e559–64.
212. Centers for Disease Control and Prevention (2007) Exposure to secondhand smoke among students aged 13–15 years-worldwide, 2000–2007. MMWR 56: 497–500.

213. Rothman KJ (1986) Modern epidemiology. Boston: Little, Brown and Co. p.39.
214. US Department of Health and Human Services (2007) Children and secondhand smoke exposure. Excerpts from The Health Consequences of Involuntary Exposure to Tobacco Smoke: A Report of the Surgeon General. Atlanta: US Department of Health and Human Services, Centers for Disease Control and Prevention, Coordinating Center for Health Promotion, National Center for Chronic Disease Prevention and Health Promotion, Office on Smoking and Health.
215. US Department of Health and Human Services (1986) The Health Consequences of Involuntary Smoking. A Report of the Surgeon General. Rockville (Maryland): US Department of Health and Human Services, Public Health Service, Centers for Disease Control, Center for Health Promotion and Education, Office on Smoking and Health.
216. Pérez-Ríos M, Schiaffino A, López MJ, Nebot M, Galán I, et al. (2013) Questionnaire-based second-hand smoke assessment in adults. Eur J Public Health 23: 763–767
217. Wahn U (2000) What drives the allergic march? Allergy 55: 591–599.

Editors' Summary

Background. The immune system protects the human body from viruses, bacteria, and other pathogens. Whenever a pathogen enters the body, immune system cells called T lymphocytes recognize specific molecules on its surface and release chemical messengers that recruit and activate other types of immune cells, which then attack the pathogen. Sometimes, however, the immune system responds to harmless materials (for example, pollen; scientists call these materials allergens) and triggers an allergic disease such as allergic rhinitis (inflammation of the inside of the nose; hay fever is a type of allergic rhinitis), allergic dermatitis (also known as eczema, a disease characterized by dry, itchy patches on the skin), and food allergy. Recent studies suggest that all these allergic (atopic) diseases are part of a continuous state called the "atopic march" in which individuals develop allergic diseases in a specific sequence that starts with allergic dermatitis during infancy, and progresses to food allergy, allergic rhinitis, and finally asthma (inflammation of the airways).

Why Was This Study Done? Allergic diseases are extremely common, particularly in children. Allergic rhinitis alone affects 10%–30% of the world's population and up to 40% of children in some countries. Moreover, allergic diseases are becoming increasingly common. Allergic diseases affect the quality of life of patients and are financially costly to both patients and health systems. It is important, therefore, to identify the factors that cause or potentiate their development. One potential risk factor for allergic diseases is active or passive exposure to tobacco smoke. In some countries up to 80% of children are exposed to second-hand smoke so, from a public health point of view, it would be useful to know whether exposure to tobacco smoke is associated with the development of allergic diseases. Here, the researchers undertake a systematic review (a study that uses predefined criteria to identify all the research on a given topic) and a meta-analysis (a statistical approach for combining the results of several studies) to investigate this issue.

What Did the Researchers Do and Find? The researchers identified 196 observational studies (investigations that observe outcomes in populations without trying to affect these outcomes in any way) that examined the association between smoke exposure and allergic rhinitis, allergic dermatitis, or food allergy. When all studies were analyzed together, allergic rhinitis was not associated with active smoking but was slightly associated with exposure to second-hand smoke. Specifically, compared to people not exposed to second-hand smoke, the pooled relative risk (RR) of allergic rhinitis among people exposed to second-hand smoke was 1.10 (an RR of greater than 1 indicates an increased risk of disease development in an exposed population compared to an unexposed population). Allergic dermatitis was associated with both active smoking (RR = 1.21) and exposure to second-hand smoke (RR = 1.07). In the populations of children and adolescents included in the studies, allergic rhinitis was associated with both active smoking and exposure to second-hand smoke (RRs of 1.40 and 1.09, respectively), as was allergic dermatitis (RRs of 1.36 and 1.06, respectively). Finally food allergy was associated with exposure to second-hand smoke (RR = 1.43) when cohort studies (a specific type of observational study) only were examined but not when all the studies were combined.

What Do These Findings Mean? These findings provide limited evidence for a weak association between smoke exposure and allergic disease in adults but suggest that both active and passive smoking are associated with a modestly increased risk of allergic diseases in children and adolescents. The accuracy of these findings may be affected by the use of questionnaires to assess smoke exposure and allergic disease development in most of the studies in the meta-analysis and by the possibility that individuals exposed to smoke may have shared other characteristics that were actually responsible for their increased risk of allergic diseases. To shed more light on the role of smoking in allergic diseases, additional studies are needed that accurately measure exposure and outcomes. However, the present findings suggest that, in countries where many people smoke, 14% and 13% of allergic rhinitis and allergic dermatitis, respectively, among children may be attributable to active smoking. Thus, the elimination of active smoking among children and adolescents could prevent one in seven cases of allergic rhinitis and one in eight cases of allergic dermatitis in such countries.

Additional Information. Please access these websites via the online version of this summary at http://dx.doi.org/10.1371/journal.pmed.1001611.

- The UK National Health Service Choices website provides information about allergic rhinitis, hay fever (including personal stories), allergic dermatitis (including personal stories), and food allergy (including personal stories)
- The US National Institute of Allergy and Infectious Disease provides information about allergic diseases
- The UK not-for-profit organization Allergy UK provides information about all aspects of allergic diseases and a description of the atopic march
- MedlinePlus encyclopedia has pages on allergic rhinitis and allergic dermatitis (in English and Spanish)
- MedlinePlus provides links to further resources about allergies, eczema, and food allergy (in English and Spanish)
